# Bi-allelic *LETM1* variants perturb mitochondrial ion homeostasis leading to a clinical spectrum with predominant nervous system involvement

**DOI:** 10.1016/j.ajhg.2022.07.007

**Published:** 2022-09-01

**Authors:** Rauan Kaiyrzhanov, Sami E.M. Mohammed, Reza Maroofian, Ralf A. Husain, Alessia Catania, Alessandra Torraco, Ahmad Alahmad, Marina Dutra-Clarke, Sabine Grønborg, Annapurna Sudarsanam, Julie Vogt, Filippo Arrigoni, Julia Baptista, Shahzad Haider, René G. Feichtinger, Paolo Bernardi, Alessandra Zulian, Mirjana Gusic, Stephanie Efthymiou, Renkui Bai, Farah Bibi, Alejandro Horga, Julian A. Martinez-Agosto, Amanda Lam, Andreea Manole, Diego-Perez Rodriguez, Romina Durigon, Angela Pyle, Buthaina Albash, Carlo Dionisi-Vici, David Murphy, Diego Martinelli, Enrico Bugiardini, Katrina Allis, Costanza Lamperti, Siegfried Reipert, Lotte Risom, Lucia Laugwitz, Michela Di Nottia, Robert McFarland, Laura Vilarinho, Michael Hanna, Holger Prokisch, Johannes A. Mayr, Enrico Silvio Bertini, Daniele Ghezzi, Elsebet Østergaard, Saskia B. Wortmann, Rosalba Carrozzo, Tobias B. Haack, Robert W. Taylor, Antonella Spinazzola, Karin Nowikovsky, Henry Houlden

**Affiliations:** 1 Department of Neuromuscular Diseases, University College London, Queen Square, Institute of Neurology, London WC1N 3BG, UK; 2 Department of Biomedical Sciences, Institute of Physiology, Pathophysiology and Biophysics, University of Veterinary Medicine Vienna, Vienna 1210, Austria; 3 Department of Neuropediatrics, Jena University Hospital, Jena 07747, Germany; 4 Center for Rare Diseases, Jena University Hospital, Jena 07747, Germany; 5 Unit of Medical Genetics and Neurogenetics, Fondazione IRCCS Istituto Neurologico Carlo Besta, Milan 20126, Italy; 6 Unit of Muscular and Neurodegenerative Disorders, Laboratory of Molecular Medicine, Bambino Gesù Children’s Hospital, IRCCS, Rome 00146, Italy; 7 Wellcome Centre for Mitochondrial Research, Translational and Clinical Research Institute, Faculty of Medical Sciences, Newcastle University, Newcastle Upon Tyne NE2 4HH, UK; 8 Kuwait Medical Genetics Centre, Al-Sabah Medical Area 80901, Kuwait; 9 Division of Medical Genetics, Department of Pediatrics, David Geffen School of Medicine, the University of California at Los Angeles, Los Angeles, CA 90095, USA; 10 Center for Rare Diseases, Department of Pediatrics and Department of Genetics, Copenhagen University Hospital Rigshospitalet, Blegdamsvej 9, Copenhagen 2100, Denmark; 11 West Midlands Regional Genetics Service, Birmingham Women’s and Children’s Hospital, Birmingham B15 2TG, UK; 12 Paediatric Radiology and Neuroradiology Department, V. Buzzi Children’s Hospital, Milan 20154, Italy; 13 Peninsula Medical School, Faculty of Health, University of Plymouth, Plymouth PL4 8AA, UK; 14 Paediatrics Wah Medical College NUMS, Wah Cantonment, Punjab 44000, Pakistan; 15 University Children’s Hospital, Salzburger Landeskliniken (SALK) and Paracelsus Medical University (PMU), Salzburg 5020, Austria; 16 Department of Biomedical Sciences, University of Padova, Via Ugo Bassi 58/B, Padova 35131, Italy; 17 Institute of Neurogenomics, Helmholtz Zentrum München, Neuherberg 85764, Germany; 18 DZHK (German Centre for Cardiovascular Research), Partner Site Munich Heart Alliance, Munich 81675, Germany; 19 Institute of Human Genetics, Technical University of Munich, Munich 81675, Germany; 20 GeneDx Inc, Gaithersburg, MD 20877, USA; 21 Institute of Biochemistry and Biotechnology, Pir Mehar Ali Shah Arid Agriculture University, Rawalpindi 44000, Pakistan; 22 Neuromuscular Diseases Unit, Department of Neurology, Hospital Clinico San Carlos and San Carlos Health Research Institute (IdISSC), Madrid 28040, Spain; 23 Department of Human Genetics, Division of Medical Genetics, Department of Pediatrics, David Geffen School of Medicine, University of California, Los Angeles, Los Angeles, CA 90095, USA; 24 Neurometabolic Unit, National Hospital for Neurology and Neurosurgery, London, UK; 25 Department of Chemical Pathology, Great Ormond Street Hospital, WC1N 3BG London, UK; 26 Department of Clinical Movement Neurosciences, Royal Free Campus, University College of London, Queen Square Institute of Neurology, London WC1N 3BG, UK; 27 Division of Metabolism, Bambino Gesù Children’s Hospital, IRCCS, Rome 00146, Italy; 28 Department of Clinical and Movement Neurosciences, UCL Queen Square Institute of Neurology, University College London, London WC1N 3BG, UK; 29 Core Facility of Cell Imaging and Ultrastructure Research, University of Vienna, Djerassiplatz 1, 1030 Wien, Austria; 30 Department of Genetics, Copenhagen University Hospital Rigshospitalet Blegdamsvej, Copenhagen 2100, Denmark; 31 Institute of Medical Genetics and Applied Genomics, University of Tuebingen, 72076 Tübingen, Germany; 32 Department of Neuropediatrics, Developmental Neurology and Social Pediatrics, University of Tübingen, Tübingen 72076, Germany; 33 NHS Highly Specialised Service for Rare Mitochondrial Disorders, Newcastle upon Tyne Hospitals NHS Foundation Trust, Newcastle upon Tyne NE1 4LP, UK; 34 Unit of Neonatal Screening, Metabolism and Genetics, Department of Human Genetics, National Institute of Health Dr Ricardo Jorge, Porto 4000-055, Portugal; 35 Department of Pathophysiology and Transplantation, University of Milan, Milan 20122, Italy; 36 Institute for Clinical Medicine, University of Copenhagen, Copenhagen 2200, Denmark; 37 Radboud Center for Mitochondrial Medicine, Department of Pediatrics, Amalia Children’s Hospital, Radboudumc, Nijmegen 6525 EZ, the Netherlands; 38 Centre for Rare Diseases, University of Tuebingen, Tübingen 72076, Germany; 39 Department of Internal Medicine I, ASCTR and Comprehensive Cancer Center, Medical University of Vienna, Vienna 1090, Austria

**Keywords:** mitochondria, LETM1, mitochondrial diseases, neurodegeneration, Wolf-Hirschhorn syndrome, genetics, neurology, oxidative phosphorylation, potassium transport, volume homeostasis

## Abstract

Leucine zipper-EF-hand containing transmembrane protein 1 (*LETM1*) encodes an inner mitochondrial membrane protein with an osmoregulatory function controlling mitochondrial volume and ion homeostasis. The putative association of *LETM1* with a human disease was initially suggested in Wolf-Hirschhorn syndrome, a disorder that results from *de novo* monoallelic deletion of chromosome 4p16.3, a region encompassing *LETM1.* Utilizing exome sequencing and international gene-matching efforts, we have identified 18 affected individuals from 11 unrelated families harboring ultra-rare bi-allelic missense and loss-of-function *LETM1* variants and clinical presentations highly suggestive of mitochondrial disease. These manifested as a spectrum of predominantly infantile-onset (14/18, 78%) and variably progressive neurological, metabolic, and dysmorphic symptoms, plus multiple organ dysfunction associated with neurodegeneration. The common features included respiratory chain complex deficiencies (100%), global developmental delay (94%), optic atrophy (83%), sensorineural hearing loss (78%), and cerebellar ataxia (78%) followed by epilepsy (67%), spasticity (53%), and myopathy (50%). Other features included bilateral cataracts (42%), cardiomyopathy (36%), and diabetes (27%). To better understand the pathogenic mechanism of the identified *LETM1* variants, we performed biochemical and morphological studies on mitochondrial K^+^/H^+^ exchange activity, proteins, and shape in proband-derived fibroblasts and muscles and in *Saccharomyces cerevisiae*, which is an important model organism for mitochondrial osmotic regulation. Our results demonstrate that bi-allelic *LETM1* variants are associated with defective mitochondrial K^+^ efflux, swollen mitochondrial matrix structures, and loss of important mitochondrial oxidative phosphorylation protein components, thus highlighting the implication of perturbed mitochondrial osmoregulation caused by *LETM1* variants in neurological and mitochondrial pathologies.

## Graphical abstract

**Figure:**
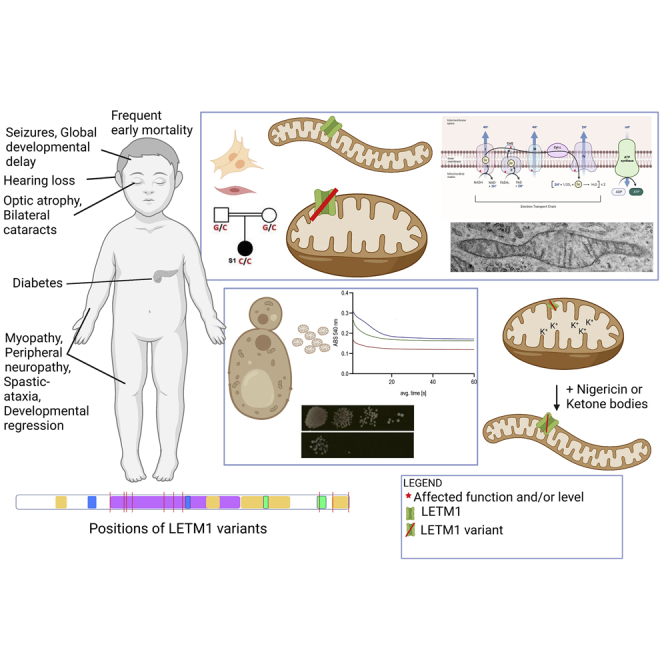

## Teaser

Kaiyrzhanov et al. describe 18 affected individuals with bi-allelic variants in the leucine zipper-EF-hand containing transmembrane protein 1 gene presenting with clinical features suggestive of a mitochondrial disease. Functional studies showed defective mitochondrial K^+^ efflux, swollen mitochondrial matrix structures, and loss of mitochondrial oxidative phosphorylation protein components.

## Introduction

Leucine zipper-EF-hand containing transmembrane protein 1 (*LETM1*) (MIM: 604407) is a ubiquitously expressed and phylogenetically highly conserved nuclear gene. LETM1, also named SLC55A1, is part of the mitochondrial transporter protein SLC55 family that belongs to the SLC solute carrier superfamily,^1^ is the founder of the LETM1 superfamily, and is listed as one of the EF-hand Ca^2+^-binding proteins of the MitoCarta library.^2^^,^^3^ The proteins of the LETM1 superfamily contain leucine zipper and several coiled-coil domains.^2^^,^^4^ LETM1 is an inner mitochondrial membrane protein with an osmoregulatory function that controls cation homeostasis, preventing their equilibration with the H^+^ electrochemical gradient. While first identified to function as an electroneutral mitochondrial K^+^-H^+^ exchanger (KHE), LETM1 has also been connected to the regulation of the uptake or extrusion of Ca^2+^.^2^^,^^5^, ^6^, ^7^, ^8^, ^9^, ^10^

The pathological hallmark of *LETM1* depletion is mitochondrial matrix swelling, fragmentation, and loss of cristae structure, consistently found in all studied organisms,^5^ whereas *LETM1* overexpression causes mitochondrial elongation, cristae swelling, and matrix condensation due to imbalance in osmotic homeostasis.^11^ Silencing *LETM1* homologs in yeast, *Fusarium graminearum,* and *Toxoplasma gondii* results in lethality or loss of virulence. *Drosophila melanogaster* with tissue-specific depleted *LETM1* displays compromised tissue growth and locomotor behavior, as well as impaired evoked synaptic release of neurotransmitters.^12^ The homozygous deletion of *LETM1* leads to developmental and embryonic lethality in flies, worms, and mice.^9^^,^^11^^,^^12^

Consistent with the vital role of mitochondrial osmoregulation, matrix swelling and cation imbalance due to *LETM1* inactivation have wide-reaching and pleiotropic effects on mitochondrial biogenesis and bioenergetics, perturbing glucose and pyruvate utilization, tryptophan and mtDNA metabolism, and outer mitochondrial membrane integrity and causing necrotic cell death.^9^^,^^12^, ^13^, ^14^, ^15^, ^16^, ^17^

The importance of *LETM1* in neuronal function and pathology was initially suggested in Wolf-Hirschhorn syndrome (WHS [MIM: 194190]).^4^ This genetic syndrome results from *de novo* monoallelic deletion of several genes on the short arm of chromosome 4. Depending on the length of the deletion, WHS might present with a combination of congenital malformations, specific facial dysmorphism, growth and cognitive impairment, microcephaly, hypotonia, and epilepsy.^13^ *LETM1* is localized in WHS critical region 2 (WHSCR2), less than 80 kb from WHS critical region 1 (WHSCR1), and is deleted in almost all individuals with the full WHS phenotype. *LETM1* is proposed to be associated with epilepsy and neuromuscular features of WHS.^18^^,^^19^ Analysis of WHS fibroblasts linked *LETM1* haploinsufficiency with mitochondrial defects. One study reports elevated intracellular Ca^2+^, decreased Ca^2+^ sensitivity of the mitochondrial permeability transition pore (PTP), and increased superoxide and hyperpolarization of the inner membrane;^20^ another study reports mtDNA aggregation, pyruvate dehydrogenase (PDH) deficiency, and a preferential shift from pyruvate oxidation to ketone body utilization.^14^ How the cation transport properties of LETM1 and the broad effects of its dysfunction on other mitochondrial and cellular functions mechanistically contribute to the WHS disease phenotypes is not well understood and is complicated by the multigenic cause for WHS. Other implications of LETM1 impairment in genetic diseases include temporal lobe epilepsy,^21^ diabetes,^22^ and obesity.^15^

Here, we describe 18 affected individuals from 11 unrelated families presenting with clinical features suggestive of a mitochondrial disease largely involving the CNS in which exome sequencing (ES) identified novel and ultra-rare bi-allelic segregating *LETM1* variants.

To functionally characterize the bi-allelic *LETM1* variants, we explored cellular growth and mitochondrial respiratory chain, morphology, osmotic regulation, and KHE activity in proband-derived fibroblasts, muscle samples, and yeast carrying the variants of interest.

## Subjects and methods

### Study subjects

Using the GeneMatcher platform^23^ and data sharing with collaborators around the world, 11 families with bi-allelic *LETM1* variants were identified. The affected individual from family 8 was recruited from the report by Catania et al.^24^ describing a person with a combined pituitary hormone deficiency, ocular involvement, myopathy, ataxia, and mitochondrial impairment carrying variants in several putative disease-causing genes, including rare bi-allelic variants in *OTX2* (orthodenticle homeobox 2 [MIM: 600037]) and *LETM1* as well as rare heterozygous variants in *AFG3L2* (AFG3 like matrix AAA peptidase subunit 2 [MIM: 604581]) and *POLG* (DNA polymerase gamma, catalytic subunit [MIM: 174763]). Clinical details of the cohort were obtained by the follow-up of the living affected individuals and retrospective analysis of the available clinical records for deceased individuals. Parents and legal guardians of all affected individuals gave their consent for the publication of clinical and genetic information according to the Declaration of Helsinki, and the study was approved by The Research Ethics Committee Institute of Neurology University College London (IoN UCL) (07/Q0512/26) and the local Ethics Committees of each participating center. Consent has been obtained from families 1, 5, and 8 to publish medical photographs and video examinations. Brain magnetic resonance imaging (MRI) scans were obtained from 6 affected individuals and were reviewed by an experienced pediatric neuroradiologist (FA).

### Exome sequencing and data analysis

Proband only or trio ES in 11 families was carried out in DNA extracted from blood-derived leukocytes in 9 different centers following slightly different protocols (see Table 1). ES data analysis and variant filtering and prioritization were performed using in-house implemented pipelines of the local genetic centers (Table 1). Sanger sequencing was performed to confirm co-segregation in all available family members.

**Table 1:** Summary of the *LETM1* variants identified in the present cohort and 2 non-pathogenic variants

F ID	Center	Method	gDNA Change (chr4 hg 19)	Variant type	nt change	aa change	gnomAD V3.1.2 and V2.1.1	Other databases	CADD	GERP	SIFT	PolyPhen
1	Queen Square Genomics	proband only ES^25^, ^26^, ^27^	g.1834673A>T	missense, splice region	c.878T>A	p.Ile293Asn	0	0	28.8	4.61	D	PD
1	Queen Square Genomics	proband only ES^25^, ^26^, ^27^	g.1816277T-	frameshift	c.2094del	p.Asp699Metfs^∗^13	0	1 het allele (UKBB)	–	–	–	–
2	Copenhagen University Hospital	proband only ES^28^	g.1816151C>G	stop_loss	c.2220G>C	p.^∗^740Tyrext26	0	0	–	–	–	–
7	Queen Square Genomics	proband only ES^26^^,^^27^										
3	Wellcome Center for Mitochondrial Research	proband only ES^29^	g.1836692CTT-	inframe deletion	c.754_756del	p.Lys252del	0	0	–	–	–	–
4	Wellcome Center for Mitochondrial Research	proband only ES^29^	g.1834670C>T	missense	c.881G>A	p.Arg294Gln	4 het alleles (V2.1.1); 2 het alleles (V3.1.2.)	2 het alleles (UKBB); 3 het alleles (GeneDx); 2 het alleles (TOPMed)	26.3	4.61	D	PD
8	Fondazione IRCCS Istituto Neurologico Carlo Besta, Milan	proband only ES^24^										
5	Institute of Medical Genetics and Applied Genomics, University of Tuebingen, Germany	proband only ES^30^	g.1834479C>T	missense	c.1072G>A	p.Asp358Asn	0	0	23.5	4.61	D	B
6	GeneDX	trio ES^31^, ^32^, ^33^	g.1827313-C-T^a^	missense	c.1178G>A	p.Arg393His	13 het alleles (V2.2.1.1); 2 het alleles (V3.1.2)	AF 0.0002 (1K GP); 1 het allele (UKBB); 7 het alleles (GeneDx); 7 het alleles (TOPMed)	26.6	5.06	D	PD
9	Exeter Genomics Laboratory	trio ES^34^	g.1827352C>G	missense	c.1139G>C	p.Arg380Pro	0	1 het allele (UKBB)	27.4	5.06	D	PD
10	Institute of Human Genetics, Technical University of Munich	proband only ES^35^	g.1814582G>C	splice defect	c.2071−9C>G	p.Val691fs^∗^4	0	0	–	–	–	–
11	Bambino Gesù Children’s Hospital, IRCCS	proband only ES^36^, ^37^, ^38^	g.1834653G>A	missense	c.898C>T	p.Pro300Ser	0	2 het alleles (GeneDx)	25.8	4.61	D	PD
Non-pathogenic variant 1			g.1834638T>G	missense	c.913A>C	p.Ile305Leu	1 het allele (V2.1.1); 3 het alleles, 1 hom allele (V3.1.2)	4 het alleles (TOPMed)	27.6	4.61	D	PD
Non-pathogenic variant 2			g.1818625T>A	missense	c.1760A>G	p.Lys587Arg	2,756 het alleles, 39 hom alleles (V2.1.1); 2,354 het alleles, 34 hom alleles (V3.1.2)	43,024.9 het alleles, 1 hom allele; AF 0.002, 4 hom carriers (UKBB); 4,367 het alleles and 82 hom alleles (TOPMed)	25.3	5.04	D	PD

*LETM1* isoform is GenBank: NM_012318.3. F, family; ES, exome sequencing; gDNA, genomic DNA; nt, nucleotide; aa, amino acid; D, deleterious; PD, probably damaging; AF, allele frequency; het, heterozygous; hom, homozygous. Other databases: Queen Square Genomics database (23K exomes), ESP, Iranome, 1K GP (1000 Genomes global minor allele frequency), UKBB (UK Biobank), GeneDx database, Middle Eastern database, TOPMed.

a A homozygous *LETM1* variant due to maternal uniparental disomy.

### Skin biopsy and primary fibroblast culture and muscle biopsy

Individuals F1:S1, F1:S2 and parents (mother-F1:M, father-F1:F), F2:S1, F5:S1, F10:S1, F11:S1, and F11:S2 provided each one skin biopsy, and affected individuals F11:S1, F11:S2, and F5:S1 provided also each one muscle biopsy. Details on fibroblast cell lines establishment and muscle sample preparations are described in the supplemental material and methods.

### Western blotting analysis

Immunoblotting analysis was performed using standard protocols as described previously;^39^ detailed descriptions of sample preparation, quantification, and western blotting are in the supplemental material and methods. A list of antibodies used for this study is given in supplemental data.

### Cell imaging

Confocal microscopy was performed for fibroblasts from F1, F2, F5, F10, and F11 and respective control subjects following established protocols for life and immune staining described in Durigon et al.,^14^ Wilfinger et al.,^40^ and supplemental material and methods. Transmission electron microscopy is described in the supplemental material and methods.

### mtDNA copy number

DNA was extracted from muscle or fibroblasts by proteinase K treatment. The mtDNA content was determined by quantitative real-time PCR using two independent mitochondrial and four independent nuclear DNA sequences as previously described.^41^

### Immunohistochemistry

FFPE muscle tissue was cut with a microtome in 4 μm slides. Immunohistochemistry was performed as described previously in Kusikova et al.^39^ with some modifications (a detailed description of the method is given in the supplemental material and methods). All antibodies used in this experiment are listed in supplemental material and methods.

### Plasmid and *LETM1* single-nucleotide variants

Full-length human *LETM1* cDNA fused to C-terminal Hemagglutinin (*HA*)-tag and subcloned into the multi-copy plasmid pVT-103U^42^ served as a template to introduce the *LETM1* variants by site-directed mutagenesis. Amino acid replacements and deletions were performed with non-overlapping back-to-back annealing mutagenic primers, using the Q5 site-directed mutagenesis kit (NEB #E0552S) with NEB 5-alpha competent *E. coli* cells (NEB #C2987). All primers were from Microsynth and all the identified variants were confirmed by DNA sanger sequencing. To distinguish the phenotypes of disease-associated *LETM1* variants and non-pathogenic variants, two non-disease-associated *LETM1* (GenBank: NM_012,318.3) missense variants (rare *LETM1* variants but with homozygotes in gnomAD v3.1.1), c.913A>C (p.Ile305Leu) and c.1760A>G (p.Lys587Arg), were included in this study. A list of variants studied in yeast and primers used for site-directed mutagenesis is given in supplemental data.

### Yeast transformation

W303 (ATCC 201239) *Saccharomyces cerevisiae* strain *mdm38/letm1Δ* (lacking the open reading frame *YOL027c*, which encodes the yeast LETM1 homolog)^42^ was transformed with the multi-copy vector pVT-103U, either empty or containing wild-type human *LETM1*^42^ or *LETM1* variants using the lithium acetate/single-stranded carrier DNA/polyethylene glycol method^43^ and grown on selective media (SD-URA) to ensure the retention of the plasmids. Yeast growth media were described in Zotova et al.^44^

### Mitochondrial isolation and KOAc-induced swelling assay

Mitochondria were isolated from yeast cells logarithmically grown in SD-URA by homogenization and differential centrifugation method as described in Nowikovsky et al.^42^ and immediately used for KOAc-induced swelling assays. The protocols of Nowikovsky et al.^42^ were adapted to smaller volumes. In brief, isolated yeast mitochondria suspended in breaking buffer (0.6 M sorbitol, 20 mM Tris-HCl [pH 7.4]) were de-energized with antimycin A (2.5 μM) for 10 min at room temperature (25°C), washed, and resuspended in breaking buffer at a concentration of 200 μg/20 μL. As Mg^2+^ is a brake to the KHE,^45^ mitochondria were depleted from Mg^2+^ with A23187 (0.5 μM) and EDTA (10 mM) and transferred onto 96-well plates for measurement (200 μg/well). When indicated, quinine (200 μM) served as a control to inhibit KHE-mediated swelling. The 96-well plates were placed in the Thermo Scientific Varioskan LUX Multimode Microplate Reader. The swelling was initiated by injection of KOAc media (55 mM KOAc, 5 mM TES, 0.1 mM EDTA) to a final volume of 200 μL/well and the optical density changes at OD_540_ were immediately recorded at 25°C. Each measurement was performed in 3 independent replicates. Raw swelling data were fitted into a curve showing changes in absorbance versus time to quantify the swelling rate.

## Results

### Clinical findings

**Figure 1:**
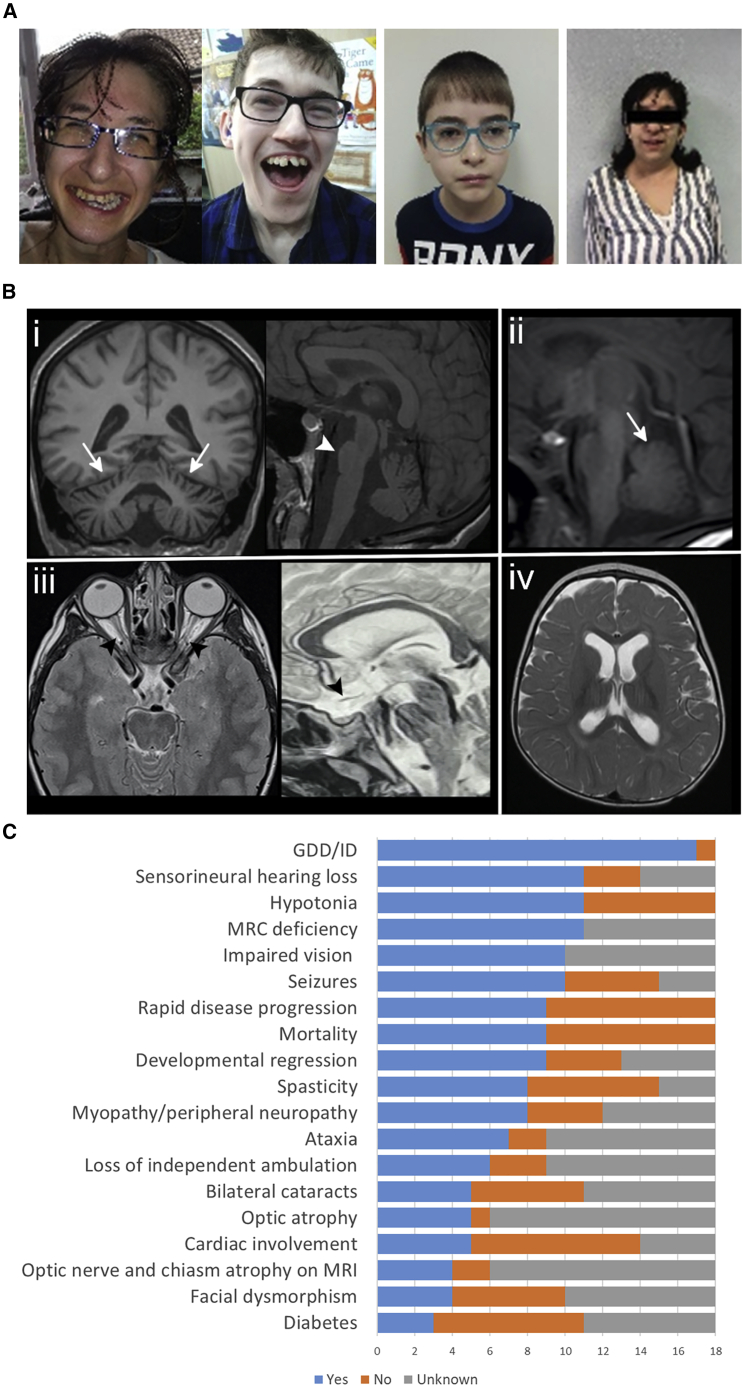
Clinical features and neuroimaging findings of the individuals with bi-allelic *LETM1* variants (A) From left to right, facial photos of the affected individuals F1:S1, F1:S2, F5:S1, and F8:S1. All persons wear glasses due to bilateral optic atrophy. All persons have prominent noses. F1:S1 and F1:S2 show long thin faces, low-set ears, and teeth abnormalities. (B) In (i) (F1:S1), severe cerebellar atrophy (arrows) and pontine hypoplasia (arrowheads) are shown, while in (ii) (F6:S1), only mild vermian hypoplasia is noted. In (iii) arrowheads point at the severe optic nerve and chiasm atrophy in 2 different individuals (F5:S1 and F7:S2). Mild ventricular dilatation is present in (iv) (F3:S1). (C) Clinical features of the affected individuals with bi-allelic *LETM1* variants. GDD, global developmental delay; ID, intellectual disability; MRI, magnetic resonance imaging; MRC, mitochondrial respiratory chain.

**Figure 2:**
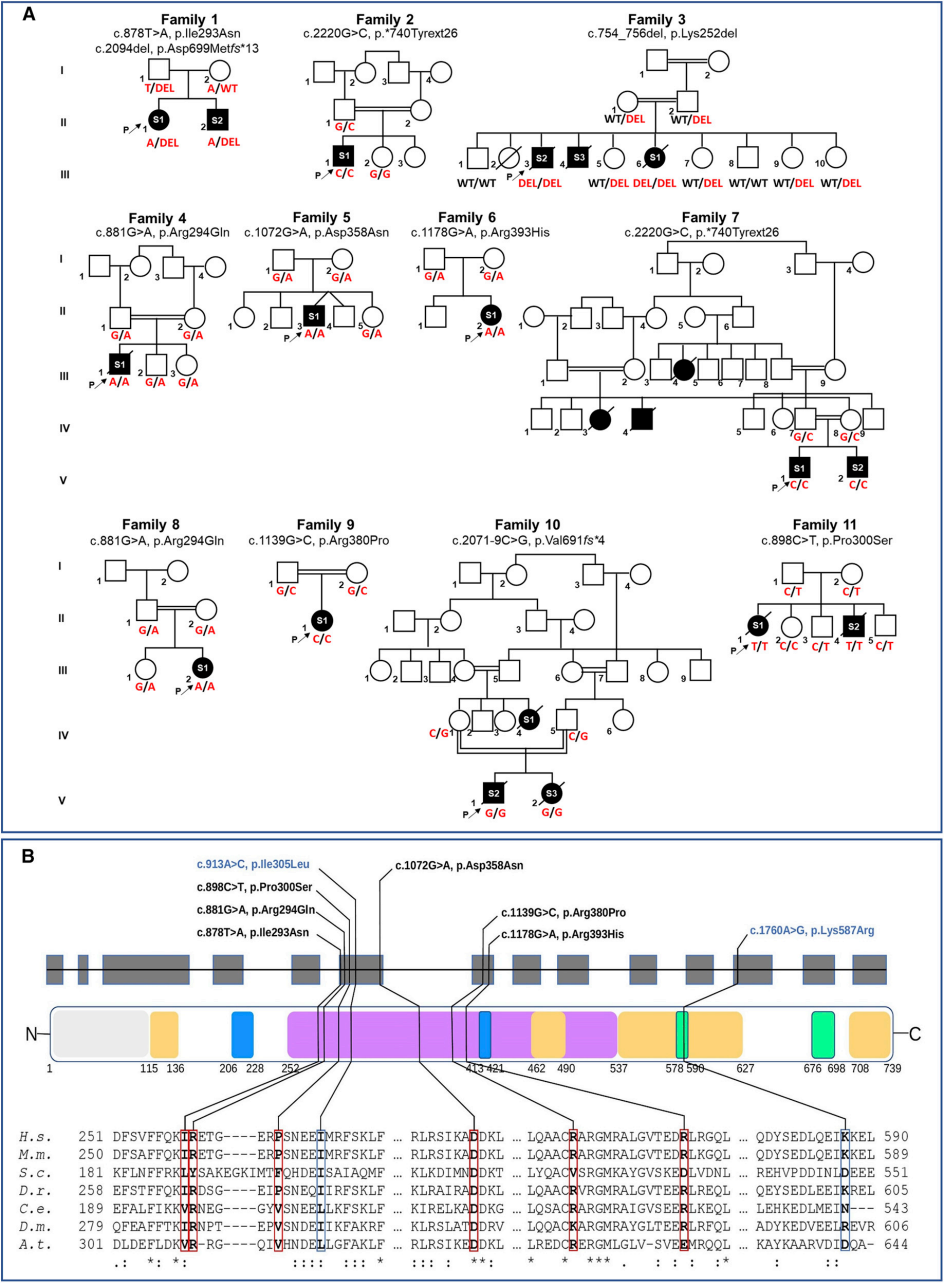
Pedigrees with the segregations of the *LETM1* variants and LETM1 protein architecture with a partial sequence alignment of the variants (A) Family trees of the individuals with bi-allelic *LETM1* variants. Square, male; circle, female; black symbols, affected individuals; white symbols, unaffected individuals. (B) Schematic representation of the human *LETM1* organization in introns, shown as a line, and exons, shown as boxes, and of LETM1 domains as indicated by the residue numbers and the color code: coiled-coil motifs, light yellow; transmembrane helices, blue; LETM/ribosomal-binding like domain, lavender; and putative EF-hands, green. All identified missense variants in the affected individuals (black) and non-pathogenic variants (blue) are mapped according to their positions. The amino acid sequence of human LETM1 was aligned with LETM1 orthologs using Clustal Omega and alignments with LETM1 from other species are shown for all segments that contain missense variants, indicated in bold red letter. Residue conservation is shown below the alignment as fully conserved (^∗^), highly conserved (:), or partially conserved (.). UniProt accession numbers for *H.s*. (*H. sapiens*), *M.m*. (*M. musculus*), *S.c.* (*S. cerevisiae*), *D.r*. (*D. rerio*), *C.e*. (*C. elegans*), *D.m*. (*D. melanogaster*), and *A.t.* (*A. thaliana*) LETM1 used in this alignment are O95202, Q9Z2I0, Q08179, Q1LY46, Q9XVM0, P91927, and F4J9G6, respectively.

The summary of the core phenotypic features of 18 affected individuals from 11 independent families with bi-allelic *LETM1* variants is provided in Table 2, Figure 1C, and Table S1. Detailed clinical history is provided in the supplemental note (case reports). Video recordings are available for affected individuals from family 1 (Videos S1, S2, S3, and S4). The cohort comprises 10 males and 8 females, 9 of whom are currently alive with a median age of 15 years (range 1–39) at the latest available follow-up (Figure 2A). Half of the persons (9/18) succumbed to their rapidly progressing disease at an early age, ranging between 2 months and 8 years old. The ethnic composition of the cohort is diverse, including families of Pakistani, Caucasus, Middle Eastern, European, and Mexican origin, with 67% of the individuals (12/18) being from consanguineous unions. Only limited clinical data were obtainable from 6 deceased persons belonging to families 3 and 10.

**Table 2:** Clinical features of affected individuals with bi-allelic *LETM1* variants

Family ID	F1		F2	F3			F4	F5	F6	F7		F8	F9	F10			F11	
Subject ID	S1	S2	S1	S1	S2	S3	S1	S1	S1	S1	S2	S1	S1	S1	S2	S3	S1	S2

**Epidemiology and medical history**																		

Sex	F	M	M	F	M	M	M	M	F	M	M	F	F	F	M	F	F	M
Current age/death age	35 y	25 y	24 y	D 1 y	D 2.7 y	D 1 y	D 8 y	11 y	17 m	15 y	8 y	39 y	1 y	D 10 m	D 2 m	D 2 m	D 6 y	D 4.5 m
Age at onset	1 y	1.5 y	2.5 y	4 m	6 m	4 m	4 m	7 m	birth	1.5 y	2 y	10 m	birth	4 m	1 m	birth	birth	birth
Type of progression	S	S	S	R	R	R	MD	MD	S	MD	MD	S	R	R	R	R	R	R
GDD/ID	+	+	+	+	+	+	+	–	+	+	+	+	+	+	+	+	+	+
Regression in development	+	+	+	N/D	–	N/D	–	–	–	+	+	+	+	N/D	N/D	N/D	+	+
Loss of ambulation (age)	+, 12 y	+, 6 y	N/D	N/D	N/D	N/D	+, 2.5 y	–	N/D	+, 5 y	+, 5 y	+, 2 y	N/D	N/D	N/D	N/D	N/D	N/D

**Main clinical features**																		

Age at last examination	35 y	25 y	24 y	>1 y	>1 y	>1 y	N/D	11 y	2 m	15 y	8 y	37	1	N/D	N/D	N/D	5 y	N/D
Small weight and height	+	+	+	N/D	N/D	N/D	N/D	+	–	+	+	+	+	N/D	N/D	N/D	–	–
Facial dysmorphism	+	+	–	N/D	N/D	N/D	–	–	+	–	–	+	–	N/D	N/D	N/D	N/D	N/D
Optic atrophy/impaired vision	+	+	+	N/D	+	N/D	+	+	N/D	+	+	+	N/D	N/D	N/D	N/D	+	N/D
Cataract	–	–	+	N/D	N/D	N/D	+	–	N/D	–	–	+	–	N/D	N/D	N/D	+	+
Sensorineural deafness	+	+	+	N/D	+	N/D	+	+	–	–	–	+	+	+	N/D	N/D	+	+
Hypotonia	–	–	–	+	+	+	+	+	–	–	–	+	+	–	+	+	+	+
Spasticity/hypertonia	+	+	+	N/D	N/D	N/D	–	–	–	+	+	–	–	+	+	+	–	–
Cerebellar ataxia	+	+	N/D	N/D	N/D	N/D	–	–	N/A	+	+	+	N/A	+	N/D	N/D	+	N/D
Myopathy	–	–	–	N/D	+	N/D	+	+	N/D	–	–	+	–	N/D	N/D	N/D	+	+
Hyperkinetic movement disorders	+	+	+	N/D	N/D	N/D	N/D	–	–	–	–	–	–	+	N/D	N/D	–	–
Peripheral neuropathy	+	+	N/D	N/D	N/D	N/D	–	–	N/D	–	–	–	–	N/D	N/D	N/D	+	N/D
Impaired speech/language abilities	+	+	+	N/D	N/D	N/D	–	–	–	+	+	+	N/A	N/D	N/D	N/D	N/D	N/D
Impaired/spastic/ataxic gait	+	+	+	N/A	N/A	–	–	–	N/A	+	+	+	N/A	N/D	N/D	N/D	N/D	N/D
Seizures	+	+	+	N/D	N/D	N/D	–	–	–	+	+	–	+	+	+	+	+	–
Cardiac involvement	–	–	–	N/D	N/D	+	+	–	–	–	–	–	–	N/D	+	N/D	+	+
Diabetes	+	+	–	N/D	N/D	N/D	N/D	+	–	–	–	–	–	N/D	N/D	N/D	–	–
Lactic acidosis	–	–	–	N/D	+	N/D	+	+	N/D	N/D	N/D	N/D	–	+	+	+	+	+
Raised urinary 3-MGA	–	–	+	N/D	–	N/D	–	–	–	N/D	N/D	+	–	+	+	+	N/D	N/D

**Investigations**																		

MRC deficiencies	CI, II, III, IV	CI, IV	CII	N/D	CI, II, III, IV	N/D	N/D	CI, III, IV	N/D	N/D	N/D	CI, III, IV, V	CIV	CI, IV	N/D	CI	CI, IV	CI, IV
Muscle histochemistry	+	+	N/D	N/D	+	N/D	N/D	+	N/D	N/D	N/D	+	+	N/D	N/D	N/D	+	–
Brain MRI findings	CA, PA	N/D	UR	VM	UR	N/D	BA	ONA, CHA	CVH	N/D	ONA, CHA	BA, CA	UR	N/D	N/D	N/D	CVH, BSH, VM, DM	N/D

Abbreviations: F, female; M, male; y, year; m, months; D, deceased; +, yes; −, no; N/D, no data; S, slow; MD, moderate; R, rapid; N/A, not applicable; GDD, global developmental delay; ID, intellectual disability; MCR, mitochondrial respiratory complex; C, complex; UR, unremarkable; 3-MGA, 3-methylglutaconic aciduria; CA, cerebellar atrophy; PA, pontine atrophy; VM, ventriculomegaly; BA, brain atrophy, ONA, optic nerve atrophy; CHA, chiasmal atrophy; CVH, cerebellar vermis hypoplasia; BSH, brain stem hypoplasia; DM, delayed myelination.

Video S1. F1:S1 at the age of 31 years old She is non-verbal and wears glasses and hearing aids. Teeth abnormalities could be seen. Her gait is ataxic and with support only.

Video S2. F1:S1 at age 35 years old Progression in the gait impairment could be seen.

Video S3. F1:S2 sitting on an armchair (at 21 years old) He wears glasses and hearing aids. He is able to understand some questions with a delay and obeys commands. Hearing is impaired. His speech is spastic-dysarthric. He has teeth abnormalities and bilateral clonus of the ankles.

Video S4. F1:S2 at age 25 years old He has jerky movements in the outstretched arms.

The cohort members had unremarkable prenatal histories with full-term birth in 14/15 persons (93%). Admission to the special care baby unit was necessary in 5/15 affected individuals (33%) due to respiratory, cardiac, and feeding issues during the neonatal period. Most of the persons (14/18, 78%) had an infantile-onset disease manifestation, and 4/18 (22%) presented first symptoms between the ages of 1.5 and 2 years. The common presenting symptoms were global developmental delay, cognitive and motor regression, failure to thrive, central hypotonia, respiratory distress, and feeding difficulties. The disease progressed rapidly in 9/18 (50%), moderately fast in 4/18 (22%), and slowly in 5/18 (28%) affected individuals. Developmental regression was later present in 9/13 (69%) affected individuals with loss of independent ambulation by a mean age of 5.4 ± 3.2 years (range 2–12).

On the most recent follow-up, the affected individuals displayed clinical features suggestive of a mitochondrial disorder. Impaired vision (10/10, 100%) with a mean onset age of 5.2 ± 3.1 years, which was confirmed to be due to optic atrophy in 5/6 (83%), and bilateral sensorineural hearing loss (11/14, 78%) diagnosed at a mean age of 2.6 ± 1.9 years (range from congenital up to 6 years) with hearing aids fitted in 7/10 (70%) persons were the common neurosensory abnormalities. While cognitive delay and intellectual disability (7/8, 87.5%%) and impaired speech acquisition (6/9, 67%) were among the common neurodevelopmental symptoms, more than half of the individuals displayed neuromuscular features including spasticity (8/15, 53%), hypotonia (11/18, 61%), muscular wasting (7/10, 70%), and cerebellar ataxia (7/9, 78%). Other frequent neurological symptoms were nystagmus (7/13, 54%), myopathy (6/12, 50%), hyperkinetic movement disorders (4/12, 33%), and spastic-ataxic gait (3/9, 33%) combined with brisk deep tendon reflexes (4/10, 40%), upgoing plantar response (4/9, 44%), and peripheral neuropathy (3/9, 33%).

Ten of the fifteen affected individuals (67%) developed epileptic seizures by a median age of 5 years (range 0.5–14). The seizure type ranged from infantile spasms and myoclonic jerks to absences, focal, and generalized tonic-clonic seizures. Individuals with younger age of seizure onset had frequent episodes spanning from hourly clusters of spasms at peak to seizures once per day. Two affected siblings from family 1 with seizure onset after ages 9 and 14 years, respectively, had seizures recurring either in clusters 2–3 times every 2–3 months (F1:S2) or once in 2 years (F1:S1). Pharmacoresistance and epileptic encephalopathy were confirmed in one person from family 9. Electroencephalograms, available from 4 individuals, showed background slowing (F5:S1), excessive sharp transients (F6:S1), single 3–4 Hz potentials and short trains (F2:S1), and continuous spike-and-slow wave activity, with bursts of faster activity observed during sleep, consistent with epileptic encephalopathy (F9:S1).

Other features consistent with a mitochondrial phenotype included bilateral cataracts (5/11, 45%) cardiomyopathy (5/14, 36%) with pericardial effusion (3/11, 27%), and diabetes (3/11, 27%). Craniofacial abnormalities included occipitofrontal circumference below third percentile in 2/6 persons (33%) and facial dysmorphism (4/10, 40%) with a long thin face, prominent nose, low-set ears, micrognathia, high arched palate, and teeth abnormalities (Figure 1A).

**Figure 3:**
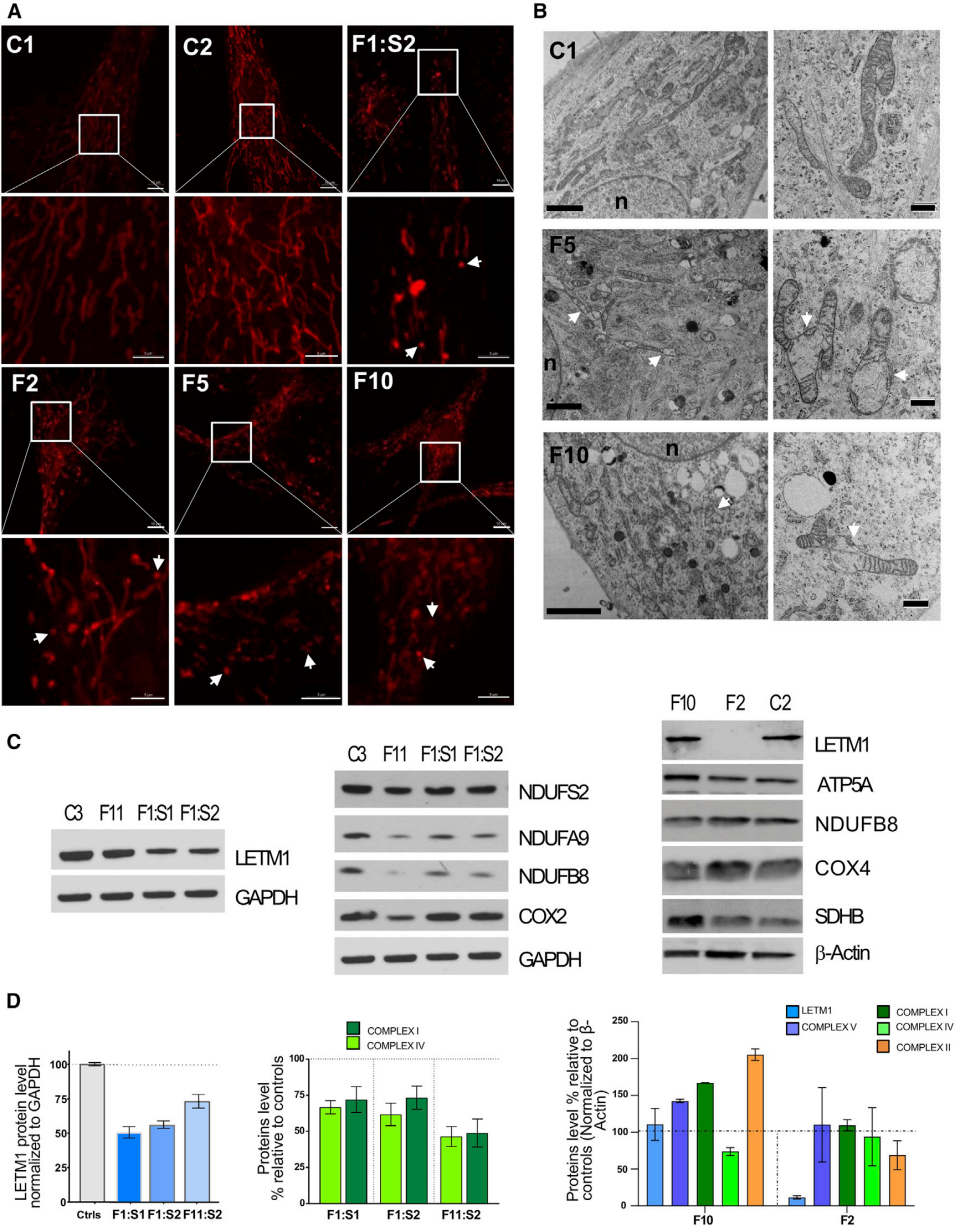
Effects of *LETM1* variants on mitochondrial morphology and proliferation in fibroblasts (A) *LETM1* variants perturb the mitochondrial network. Confocal images of fibroblasts stained with Mitotracker Red. Shown is a representative overview of the cells (bars 5 μm, except F10 10 μm) and details magnified from the box (bars 5 μm). C1 and C2, healthy donors; F1:S2, c.878T>A (p.Ile293Asn) and c.2094del (p.Asp699Metfs^∗^13); F2, c.2220G>C (p.^∗^740Tyrext26); F5, c.1072G>A (p.Asp358Asn); F10, c.2071−9C>G (p.Val691fs4^∗^). Arrow indicates representative fragmented mitochondria. For statistics, see Figure S1C. (B) *LETM1* variants cause swollen mitochondria and loss of cristae. The ultrastructure of control (C1) and affected individual (F5 and F10) fibroblasts was investigated by transmission electron microscopy and images show overviews (left panels, bars 2 μm) and details (right panels, bar 500 nm). Arrow indicates swollen mitochondria. (C and D) Variants differently affect LETM1 stability and OXPHOS proteins in fibroblasts samples. Total lysates of fibroblasts were analyzed by immunoblotting using the indicated antibodies, GAPDH, or β-actin as loading control: C2 and C3, healthy donors; F1:S1 and F1:S2, c.[878T>A; 2094del], p.[Ile293Asn; Asp699Metfs^∗^13]; F2, c.2220G>C (p.^∗^740Tyrext26); F10, c.2071−9C>G (p.Val691fs4^∗^); F11, c.898C>T (p.Pro300Ser) (C). Quantitative graphs from independent experiments representing the protein bands, normalized to the housekeeping proteins, and calculated as a percentage of controls; data are expressed as mean ± SEM (n ≥ 3 independent experiments) (D).

While not every person had available electrophysiological investigations, biochemical, metabolic studies, and muscle histochemical analysis, the obtainable tests suggested the presence of mitochondrial dysfunction in the affected individuals. Hence, electromyography and nerve conductions studies available from 5 individuals showed neurogenic (3/5) and myopathic changes (2/4). Elevated serum lactate was confirmed in 8/12 (67%) affected individuals. Plasma amino acids were abnormal in 4/9 tested with mildly elevated alanine (501–597 μmol/L, normal range 232–494), glycine, and serine. CSF-alanine was tested and mildly increased in 2 probands. Urine amino acids were tested in 4 persons, and only one person showed abnormal results including increased levels of aspartic, serine, and glycine. Urine organic acids were analyzed in 11 affected individuals and were abnormal in 9 of them with 3-methylglutaconic acid excretion (5/11), moderately elevated beta-hydroxybutyrate and acetoacetate (1/11), and significant elevation of adipic acid (1/11). Muscle biopsy was available from 7 persons and of these, 5 had abnormal findings including scattered necrotic and regenerating COX-deficient fibers with an excess of internal nuclei, lipid depositions within fibers and prominent mitochondrial pattern in vacuolated fibers (F3:S2), COX-deficient multiple ragged-red fibers with increased fiber unisometry (F8:S1), type I fiber predominance with mild glycogen storage (F9:S1), and COX-deficient fibers (F11:S1). Respiratory chain enzyme (RCE) analysis was performed in 11 individuals showing isolated or combined mitochondrial respiratory chain deficiencies in all persons tested (Tables 2, S1, Figure 3C).

Brain MRI investigations were available for 6 persons, performed between 6 days and 32 years of age (Figure 1B). In some persons, only a few sections or low-quality images could be reviewed. In 4/6 affected individuals, optic nerve and chiasm atrophy were present and in two persons optic nerves were normal. Three individuals showed infratentorial abnormalities, with severe pontine hypoplasia and cerebellar atrophy in a proband from family 1 and mild vermian hypoplasia in 2 probands from family 3 and family 6. Other minor and non-specific findings were mild supratentorial atrophy and mild ventricular dilatation noted in 2 persons each.

The affected individual from family 8 was the oldest member of the cohort showing a phenotype consistent with the rest of the individuals that survived into adulthood.

### Molecular genetic findings

In all probands, ES at the local genetic centers did not identify causative variants in known disease-associated genes. Filtering for novel (i.e., not present in available databases) and rare protein-altering variants identified bi-allelic variants in *LETM1* (GenBank: NM_012,318.3) in probands from all families (Table 1). Segregation by Sanger sequencing in families with proband-only ES and where available trio ES supported *LETM1* as a candidate gene (Figure 2A). The proband from family 6 carried a homozygous c.1178G>A (p.Arg393His) variant in *LETM1* resulting from maternal uniparental disomy. Known pathogenic variants in mtDNA and mtDNA rearrangements were excluded in all families.

The *LETM1* variants (Table 1 for variant characterization and Figure 2B) comprised missense variants causing changes in amino acid charge, size, hydrophobic or “helix breaker” properties, and frameshift variants causing premature or delayed termination. All detected missense variants were located specifically within the conserved LETM domain, while the frameshift variants were localized to the C-terminal part of LETM1 (Figure 2B). Of all the amino acid changes, the only fully conserved amino acid across mammals, vertebrates, invertebrates, plants, and yeast is Asp358, and the semi-conserved ones are Lys252 and Ile293 (Figure 2B). Arg294, which is affected by the missense variant c.881G>A (p.Arg294Gln), is conserved in all sub-families excluding yeast, and it was found in two independent persons (F4:S1 and F8:S1) of Egyptian and Italian origin, respectively. Pro300, affected by the variant c.898C>T (p.Pro300Ser), is conserved in mammals and zebrafish. Four variants affect the C-terminal stretch of human LETM1 that is absent in the yeast LETM1 homolog (Letm1p/Mdm38p) as its protein sequence is shorter. The splice variant c.2071−9C>G (p.Val691fs4^∗^) (Sashimi plot, Figure S2, supplemental material and methods) affects two residues conserved across mammals, zebrafish, worms, and plants and introduces a premature stop codon before the second EF loop. The variant c.2094del removes Asp699, a negatively charged residue, well-conserved in mammals, fish, worms, and plants that locates close to the second EF loop and prematurely terminates the protein sequence. The stop-loss variant c.2220G>C (p.^∗^740Tyrext26) leads to an elongation of 26 amino acids. This variant was present in two independent families of Pakistani origin suggesting a possible founder effect. Five of the ten identified *LETM1* variants were absent across a number of large genetic databases (∼1 million alleles), whereas the remaining four variants appear to be ultra-rare (Table 1).

### Genotype-phenotype correlation

A remarkable interfamilial phenotypic variability was observed in the present cohort. Four persons from families 1, 2, and 8 have survived into adulthood albeit with a significant disability, while 10 individuals from families 3, 4, 9, 10, and 11 had a rapidly progressing disease course leading to early death in 9 of them. Affected individuals from family 5 (age 11 years), family 6 (age 17 months), and family 7 (ages 8 and 15 years) displayed less severe phenotypes. Affected individuals from family 4 and family 8 carrying the recurrent missense *LETM1* c.881G>A (p.Arg294Gln) variant exhibited a similar range of symptoms, though F4:S1 displayed more rapid disease progression with significant cardiac involvement and early mortality. Persons of Pakistani origin from family 2 and family 7 with loss-of-function (LoF) *LETM1* c.2220G>C (p.^∗^740Tyrext26) variant were reported with a similar phenotypic range, which was more severe in family 2, possibly due to older age and longer disease course. No significant intrafamilial phenotypic variability was observed in the cohort.

### Effects of the *LETM1* variants on patient-derived fibroblasts and muscle tissue

Loss of mitochondrial volume homeostasis is the most characteristic and universally accepted phenotype of *LETM1* deficiency in human, animal models, plants, and yeast, which leads to mitochondrial fragmentation, matrix swelling, and disorganized cristae as reviewed in Austin et al.^5^ Therefore, we first evaluated the mitochondrial morphology in the available fibroblasts. Compared to fibroblasts from healthy donors (C1–C4), fibroblasts from F1:S1 and F1:S2 (compound heterozygous for c.[878T>A; 2094del], p.[Ile293Asn; Asp699Metf^∗^13]), F10 (homozygous for c.2071−9C>G [p.Val691fs4^∗^]), F2 (homozygous for c.2220G>C [p.^∗^740Tyrext26]), F5 (homozygous for c.1072G>A [p.Asp358Asn]), and F11:S2 (homozygous for c.898C>T [p.Pro300Ser]) displayed mitochondrial alterations, with significantly increased fragmented shapes seen as donut segments and punctate and enlarged units often separated from the main network (Figures 3A and S1A–S1C). Elongated mitochondrial shapes were restored by ketone bodies and by nigericin, as both reverted the ratio of elongated tubules versus fragmented units to control levels (Figure S1C). The use of the membrane potential-dependent mitochondrial dye Mitotracker Red (MTR) revealed an irregular polarization pattern of the mitochondrial network of all affected individuals, with partly depolarized tubules and hyperpolarized patches, as well as a markedly reduced electric potential of mitochondria in F10 and trend-wise also in F5 (Figures 3A, S1A, and S1C). Impaired KHE activity in LETM1-deficient cells leads to uncompensated electrophoretic K^+^ uptake and consequent mitochondrial swelling.^13^ Treatment with the synthetic KHE nigericin to counteract the loss of K^+^ homeostasis reverted the decreased membrane potential to control levels in F10 and F5 (Figures S1A and S1C), while addition of ketone bodies had no beneficial effect. Consistent with the electrophoretic K^+^ influx rate exceeding the K^+^ release rate due to a lack of KHE activity, mitochondria in F11:S2 cells readily underwent swelling and depolarization (as assessed by *in situ* staining with the potentiometric probe TMRM) upon the addition of low concentrations of valinomycin, a selective K^+^ ionophore that allows electrophoretic K^+^ uptake unlike mitochondria of control fibroblasts. Treatment of F11:S2 fibroblasts with the ionophore nigericin restored the mitochondrial sensitivity to valinomycin, a strong indication that the response to valinomycin was indeed due to lack of KHE activity (Figure S1D). Based on the protective effect of ketone bodies as an energy source for LETM1-deficient cells,^14^ we tested next whether a tubular network could be better maintained as a result. Ketone bodies suppressed MTR fluorescence in fibroblasts from F1 and F2 and attenuated its intensity in F10:S1. However, increasing the laser intensity, an elongated tubular shape of the mitochondrial network also became apparent in the samples of F1:M and F5:S1 (Figure S1A). Thus, elongation of mitochondrial tubules was accompanied by a reduced inner membrane potential, a phenomenon previously described in the context of transient matrix contraction.^46^ Replacement of glucose with galactose, known to suppress glycolytic ATP production, in F1:S1 and F11:S2 for up to 5 days produced a more dramatic morphological phenotype, in some persons resembling LETM1 siRNA (Figures S1B) and Durigon et al.^14^ and it caused cell death after only 48–72 h in F11:S2. Transmission electron microscopy was performed for F5 and F10 fibroblasts as well as control fibroblasts and confirmed ultrastructural mitochondrial changes associated with *LETM1* variants compared to the elongated tubular shapes of the healthy control mitochondria (Figure 3B). Different morphological stages of mitochondrial alterations were associated with *LETM1* c.2071−9C>G (p.Val691fs4^∗^) (F10), including short tubules containing enlarged sections with reduced cristae, swollen matrix devoid of cristae, and perinuclearly distributed spherical ghost shapes resembling a mixture of mitochondrial remnants and vacuoles. Similarly, fibroblasts with the variant *LETM1* c.1072G>A (p.Asp358Asn) (F5) showed broad, short, and electron-luce mitochondria, partly devoid of cristae and intermediate shapes between mitochondria and vacuoles.

Pathological variants frequently lead to altered expression or stability of the encoded proteins, and so we assessed LETM1 protein levels via immunoblotting. The steady-state levels of LETM1 in fibroblasts from F10 were comparable to those from control subjects. Instead, LETM1 was significantly decreased in bi-allelic *LETM1* variant fibroblasts F1:S1 and F1:S2, and F11:S2, and more drastically in F2 (Figures 3C and 3D).

Because LETM1 dysfunction restricts mitochondrial respiratory capacity in yeast and mammals,^14^^,^^47^ and the clinical and metabolic findings in the affected individuals were consistent with a mitochondrial disorder, we next investigated the abundance of the oxidative phosphorylation (OXPHOS) subunits. Fibroblasts of affected individuals harboring bi-allelic *LETM1* variants displayed reduced steady-state levels of selected respiratory chain proteins of complex I and IV, in opposite to increased levels in F10 (c.2071−9C>G [p.Val691fs4^∗^]) (Figures 3C and 3D). OXPHOS proteins NUDUFB8 and NDUFA9 were decreased in F1:S1 and F1:S2 fibroblasts and to a higher extent in F11:S2 (Figures 3C and 3D). Since mitochondrial defects can limit cellular growth, we assessed the proliferation rates of the fibroblast cell lines. While proliferation was comparable for fibroblasts with the single or compound heterozygous variants (F1), extension variant (F2), or wild-type *LETM1* (*LETM1* WT), it was significantly slowed down in *LETM1* c.2071−9C>G (p.Val691fs4^∗^) (F10) and absent in *LETM1* c.1072G>A (p.Asp358Asn) (F5) fibroblasts (Figure S3).

**Figure 4:**
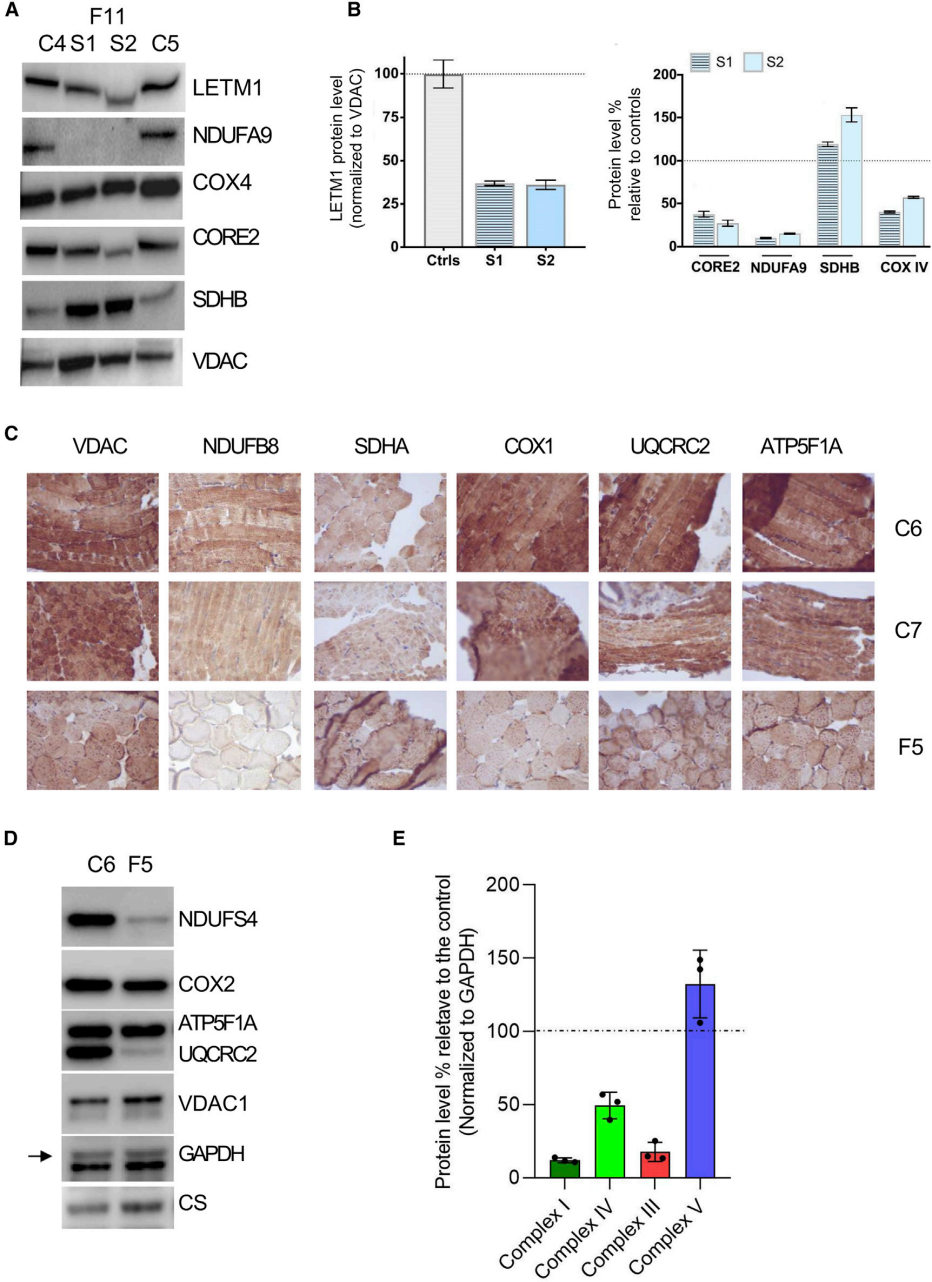
*LETM1* variants affect the stability of LETM1 and OXPHOS components in muscle samples (A and B) Western blot analysis of LETM1 and components of the OXPHOS complexes I, II, III; and IV in muscle samples from F11 and quantitative graphs. Total lysates of muscle samples from healthy donors (C4, C5) and F11 c.898C>T (p.Pro300Ser) (S1, S2) were analyzed by immunoblotting using the indicated antibodies; VDAC served as a loading control (A). Quantitative graphs represent the protein levels relative to controls and normalized to VDAC. Data are expressed as mean ± SEM; n ≥ 3 independent experiments (B). (C) Immunohistochemical staining of OXPHOS subunits and VDAC of the muscle of F5 and control subjects. Muscle samples from healthy donors (C6, C7) and F5 c.1072G>A (p.Asp358Asn) were stained for each of the five OXPHOS subunits using the indicated antibodies; VDAC served as a control. Magnification 400×. (D and E) Western blot analysis of subunits of the OXPHOS complexes, citrate synthase, and GAPDH of the muscle of F5 and control subjects. Total lysates of muscle samples were analyzed by immunoblotting using the indicated antibodies; VDAC, GAPDH, and CS served as loading controls. C6, healthy donor; F5, c.1072G>A (p.Asp358Asn) (D). Quantitative graphs representing the protein levels percentage relative to controls (normalized to GAPDH). n ≥ 3 independent experiments.

Similar to fibroblasts, LETM1 was significantly reduced in the muscle of F11 (Figures 4A and 4B). NDUFA9 (complex I) was reduced in muscles samples from F11 while SDHB (complex II) displayed a strong tissue-specific upregulation (Figures 4A and 4B). The immunohistochemistry and western blotting analysis from F5 muscle tissue (Figures 4C–4E) revealed even greater reductions for components of complexes I, III, and IV, increased SDHA, accompanied by decreased enzyme activity of complex I, and upregulated activity of complex II and citrate synthase and increased mtDNA copy number (Table S2). Proteins of the ATP synthase remained not significantly changed in all tested cell lines and tissue samples.

Overall, altered LETM1 and OXPHOS protein levels in fibroblasts and muscle samples were observed in most of the individuals. Fibroblasts cell culture data indicated that bi-allelic *LETM1* variants result in aberrant mitochondrial morphology, which was more pronounced under galactose challenge (Figure S1B) and was often lethal for F11-derived fibroblasts. Consistent with the frequently observed effect of mitochondrial defects on cellular functions and growth, cell proliferation was retarded in F10 and more drastically in F5 fibroblasts. The synthetic KHE nigericin restored mitochondrial morphological aberrations and membrane depolarization, coupling mitochondrial dysfunctions and impaired K^+^ homeostasis.

### Functional compensation analysis in yeast

Considering that LETM1 controls mitochondrial volume by regulating KHE, we ectopically expressed *LETM1* variants or wild-type in the *S. cerevisiae letm1*Δ strain to explore the functional impact of *LETM1* variants on mitochondrial KHE activity. All *LETM1* variants listed in the supplemental data were included in this analysis. The loss of KHE activity in yeast *letm1* deletion mutants, the complementation by re-expression of the homologous human *LETM1* WT, and the absence of a Ca^2+^ transport system in *S. cerevisiae* mitochondria make the system ideally suited for functional complementation analysis of *LETM1* variants and determination of their pathogenicity with respect to KHE defects.

**Figure 5:**
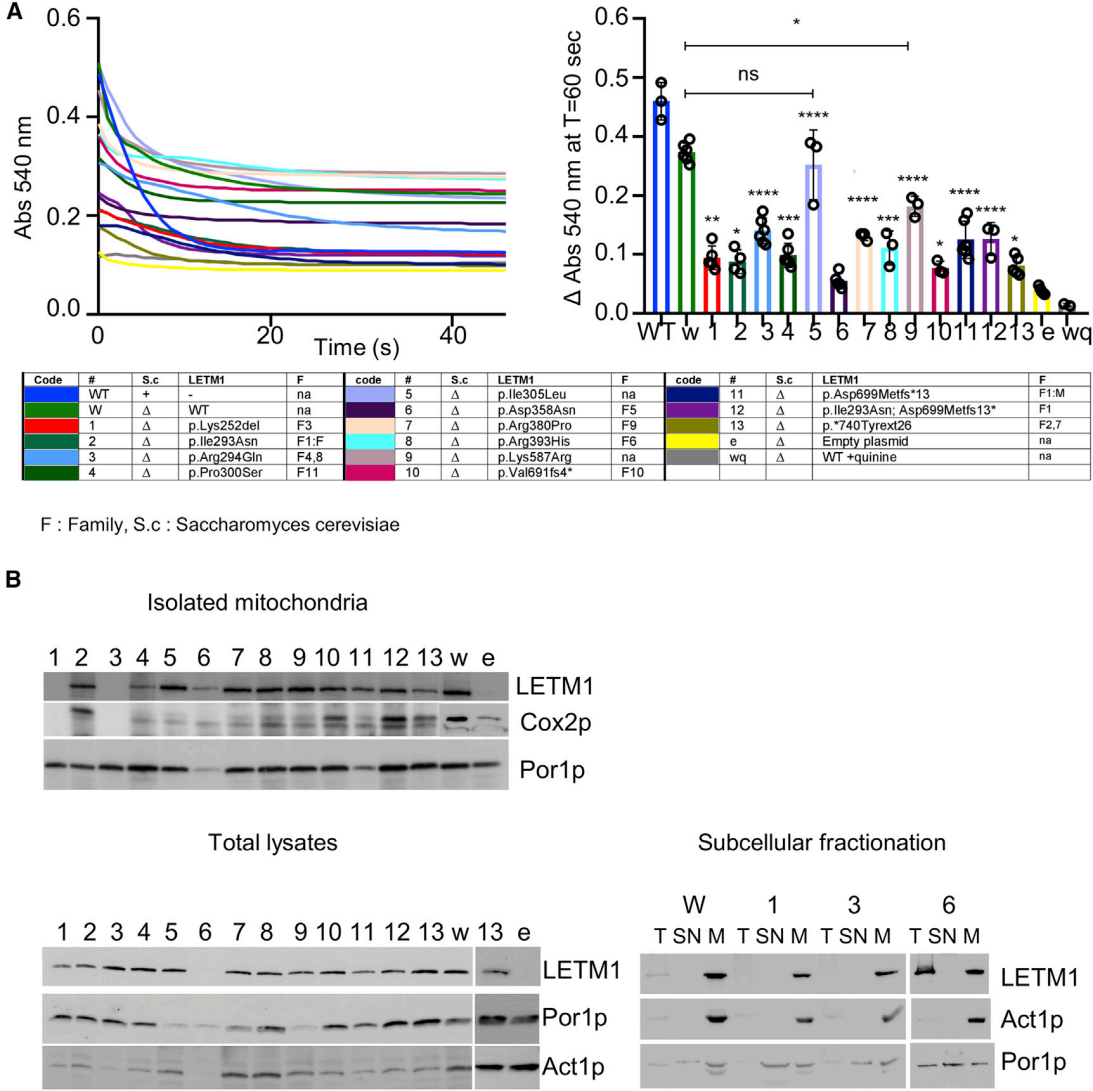
Functional implication of *LETM1* variants on yeast mitochondria (A) *LETM1* variants fail to restore KHE activity of yeast *letm1*Δ. Isolated and de-energized mitochondria were subjected to KOAc and changes of optical density at OD_540_ immediately measured. Left upper panel: representative traces of KOAc-induced swelling in *S. cerevisiae LETM1* WT mitochondria (WT, blue) or *S. cerevisiae letm1*Δ mitochondria overexpressing the empty plasmid (e, yellow) or the plasmid carrying human LETM1 WT untreated (w, green) or treated (wq, gray) with quinine or the human *LETM1* variants; color code as indicated in the inserted table: c.754–756del (p.Lys252del) (1, red), c.878T>A (p.Ile293Asn) (2, bottle green), c.881G>A (p.Arg294Gln) (3, aqua), c.898C>T (p.Pro300Ser) (4, dark green), c.913A>C (p.Ile305Leu) (5, lavender), c.1072G>A (p.Asp358Asn) (6, violet), c.1139G>C (p.Arg380Pro) (7, beige), c.1178G>A (p.Arg393His) (8, turquois), c.1760A>G (p.Lys587Arg) (9, mauve), c.2071−9C>G (p.Val691fs4^∗^) (10, purple), c.2094del (p.Asp699Metfs^∗^13) (11, dark blue), compound (12, lilac), c.2220G>C (p.^∗^740Tyrext26) (13, olive). Right upper panel: quantified rates of KOAc-induced swelling amplitudes (t = 60 s) from 3 independent experiments. An overview of the swelling rate is given in Figure S5. One-way ANOVA with Dunnett’s multiple comparisons test performed against *S. cerevisiae letm1*Δ transformed with empty pVT-103U plasmid ^∗^p = 0.0426, ^∗∗^p = 0.0026, ^∗∗∗^p = 0.0006, ^∗∗∗∗^p < 0.0001. And for p.Ile305Leu and p.Lys587Arg relatively to *S. cerevisiae letm1*Δ transformed with WT, ns > 0.05, ^∗^p = 0.0169. (B) Ectopic expression of *LETM1* variants in *S. cerevisiae letm1* Δ. Isolated mitochondria (upper panel) and total protein lysates (left lower panel) from the same strains as in (A). Subcellular fractions (T, total; SN, post-mitochondrial supernatant; M, mitochondria) (right lower panel) were immunoblotted using the indicated antibodies; Por1p and Act1p served as mitochondrial and total (and SN) loading control, respectively.

Light scattering recording of KOAc-induced swelling is a well-established method to measure the mitochondrial electroneutral exchange of K^+^ for H^+^.^48^ Exposure of de-energized mitochondria to hypotonic KOAc buffer elicits the rapid uptake of protonated acetic acid, acidification of mitochondrial matrix, and thereby activation of KHE, which results in mitochondrial K^+^ influx and water uptake and thus swelling.^45^ Isolated mitochondria from *S. cerevisiae LETM1* wild-type cells and *S. cerevisiae letm1*Δ cells overexpressing human *LETM1* WT or variants or the empty control vector were subjected to KOAc-induced swelling experiments. Recording KHE activity by measuring the decrease in optical density (OD) using light scattering techniques allows discrimination of its main determinants: initial OD, indicating the osmotic state of mitochondria before KOAc addition, and KHE exchange rate per second, indicated by the amplitude from initial to final OD as a function of the time required to achieve it. As shown in Figure 5A, KOAc-induced swelling was sensitive to the KHE inhibitor quinine, confirming the correlation of optical density with KHE activity. Knockout of *S. cerevisiae LETM1* (*S. cerevisiae letm1*Δ) entirely abolished KHE activity, as illustrated by low initial OD and swelling amplitude, which were restored by expression of *LETM1* WT. The non-pathogenic variants (p.Ile305Leu and p.Lys587Arg) performed as well as *LETM1 WT* for the initial OD, and almost as well for the kinetics values. LETM1 with the variant p.Val691fs4^∗^ (F10) almost restored the initial OD, and so did *LETM1* p.Lys587Arg (F9) and p.Arg393His (F6) but their swelling amplitudes were very low. Expression of *LETM1* variants c.754–756del (p.Lys252del) (F3), c.878T>A (p.Ile293Asn) (F1:M), or c.2220G>C (p.^∗^740Tyrext26) (F2, F7) marginally compensated K^+^ fluxes with extremely slow swelling kinetics; swelling traces for *S. cerevisiae letm1*Δ transformed with *LETM1* c.881G>A (p.Arg294Gln) (F4, F8) or *LETM1* c.2094del (p.Asp699Metfs^∗^13) (F1:F) suggested uncontrolled cation leakage (Figure 5A). Overexpression of *LETM1* c.1072G>A (p.Asp358Asn) did not rescue KHE. Taken together, these results suggest that mitochondrial reduced K^+^ flux dynamics and swollen matrix are indicative of the functional impact of disease-associated *LETM1* variants.

LETM1 protein levels associated with *LETM1* variants were examined using total cell lysates and isolated mitochondria. In comparison to the mitochondrial loading control (Porin, Por1p), LETM1 total protein levels from ectopic *LETM1* WT or variant expression were similar, except those from F1:S1-S2 (*LETM1* c.[878T>A; 2094del], p.[Ile293Asn; Asp699Metfs13^∗^]), F2 and F7 (both *LETM1* c.2220G>C [p.^∗^740Tyrext26]), and F11 (*LETM1* c.898C>T [p.Pro300Ser]), which showed reduced LETM1 levels in mitochondria (Figure 5B upper panel). The levels of LETM1 from the F5 *LETM1* variant (c.1072G>A [p.Asp358Asn]) were also low, but not when normalized to Por1p, which was similarly decreased (Figure 5B). LETM1 levels from the variants identified in F3 (c.754–756del [p.Lys252del]) and in F4 and F8 (both c.881G>A [p.Arg294Gln]) were detectable in total lysates and mitochondria prepared from a large-scale intracellular fractionation (Figure 5B right lower panel) but were also reduced. None of the ectopic expression of *LETM1* variants, however, affected the mitochondrial subcellular localization.

As previously noticed^17^^,^^49^^,^^50^ and shown here (Figure 5B), Cox2p (subunit of CIV) is reduced in *S. cerevisiae letm1*Δ strains. Cox2p levels were restored upon ectopic expression of human *LETM1* WT or *LETM1* c.878T>A (p.Ile293Asn) (F1:M), *LETM1* c.2071−9C>G (p.Val691fs4^∗^) (F10), or *LETM1* c.[878T>A; 2094del], p.[Ile293Asn; Asp699Metfs13^∗^] (F1:S1, F1:S2), but remained absent upon expression of *LETM1* c.754–756del (p.Lys252del) (F3), *LETM1* c.881G>A (p.Arg294Gln) (F4, F8), or *LETM1* c.1072G>A (p.Asp358Asn) (F5) (Figure 5B).

*S. cerevisiae letm1*Δ shows poor growth on non-fermentable (YPG) substrate.^42^ To determine the significance of the *LETM1* variants in rescuing the growth defects of *S. cerevisiae letm1*Δ compared to human *LETM1* WT, serial dilutions of *S. cerevisiae letm1*Δ strains overexpressing an empty plasmid or *LETM1* variants or WT were spotted onto fermentable (YPD) and non-fermentable (YPG) plates and grown at 30°C or 37°C (Figure S4). We found a detrimental effect of the mutant phenotype by ectopic expression of *LETM1* c.1072G>A (p.Asp358Asn) (F5) variant; this strain was able to grow on selective media but showed worsened growth defect on complete media. Growth was also slowed down at 37°C on YPD by *LETM1* c.898C>T (p.Pro300Ser)) (F11). On YPG, a marginal rescue was obtained by ectopic expression of *LETM1* c.881G>A (p.Arg294Gln) (F4, F8), *LETM1* c.2071−9C>G (p.Val691fs4^∗^) (F10), or LETM1 c.[878T>A; 2094del], p.[Ile293Asn; Asp699Metfs13^∗^] (F1:S1, F1:S2) variants.

In summary, ectopic expression in *S. cerevisiae letm1*Δ of human *LETM1* variants associated with clinical presentations phenocopied *S. cerevisiae letm1* loss of function, whereas expression of wild-type LETM1 restored the yeast deletion defects in non-fermentable growth and mitochondrial KHE exchange.

## Discussion

LETM1 function is required for the maintenance of mitochondrial cationic and osmotic balance, and swelling of the matrix due to impaired LETM1 has far-reaching consequences. Matrix swelling is supported by the unfolding of inner membranes and loss of cristae invaginations and results in dilution of metabolic substrates. Here, we found that bi-allelic *LETM1* variants identified in the affected individuals with severe clinical features differently affected the LETM1 levels and led to the typical aberrant mitochondrial morphology previously described for LETM1-deficient cells. Several OXPHOS subunits were downregulated in fibroblasts or muscle tissue, enzymatic activities were reduced, and mtDNA copy number increased. The fact that nigericin, the synthetic KHE, restored morphological aberrations interconnects these phenotypes to impaired K^+^ homeostasis. Decreased membrane potential or increased sensitivity to valinomycin and normalization of this sensitivity by nigericin supports the presence of a defect in K^+^/H^+^ exchange. Moreover, it is tempting to speculate that OXPHOS decreases proportionally to cristae loss. The finding that loss of KHE activity in *LETM1*-defective yeast was restored by ectopic expression of wild-type *LETM1* but not *LETM1* variants strongly support the notion of deregulated mitochondrial K^+^ homeostasis caused by the *LETM1* variants. Whether and how Ca^2+^ handling is also perturbed will need to be determined in future studies. Together with the fibroblasts, muscle biopsy, and yeast analyses, and with the prior knowledge that the mitochondrial phenotypes in cells match those caused by LETM1 haploinsufficiency, knockdown, or deletion in other eukaryotic species, the present findings amount to compelling evidence that the bi-allelic *LETM1* variants are the cause of the disease in the pedigrees reported in this study.

### Diseases of mitochondrial morphology and dynamics

Defects in non-OXPHOS genes responsible for mitochondrial homeostasis including mitochondrial fission and fusion have been suggested to cause primary MD (PMD).^51^ Primarily targeting the non-bioenergetic capabilities of the mitochondria, non-OXPHOS gene defects could indirectly affect the OXPHOS system,^52^ leading to a phenotype mimicking the inactivation of RCE.^12^

While there is a plethora of non-OXPHOS genes accounting for PMD,^51^^,^^53^^,^^54^ the examples relevant to the context of the present study are the genes regulating mitochondrial shape and interorganellar communication. They regulate mitochondrial dynamics through fusion and fission processes. Defects in these genes have been emerging as a cause of a novel class of inherited neurodegenerative disorders with variable onset ranging from infancy to adulthood.^53^^,^^54^ Residing in the outer and inner mitochondrial membranes or the cytosol, upon misregulation, they cause altered mitochondrial morphology including matrix swelling, fragmentation, elongation, and abnormal cristae structure, similar to what has been observed in abnormal LETM1 function.^53^, ^54^, ^55^, ^56^ Reviews of the disease-causing genes responsible for mitochondrial dynamics are provided in Burté et al.^53^ and Navaratnarajah et al.^54^ To date, affected individuals diagnosed with diseases of mitochondrial dynamics present first and foremost with neurological symptoms.^53^^,^^54^ Being essential for the survival of all organisms tested so far and having important control over the mitochondrial osmotic balance, morphology, and dynamics, before now, bi-allelic variants in *LETM1* have not been associated with any Mendelian disorder in humans.

### Bi-allelic *LETM1* variants present with a phenotypic spectrum of MD largely involving the CNS

Here we report on the association of bi-allelic *LETM1* variants with a spectrum of predominantly infantile-onset neurological, metabolic, dysmorphic, and multiple organ dysfunction syndromes in a cohort of 18 affected individuals from 11 unrelated families. Overall, the disease had a progressive course, though with variable rates of deterioration. Hence, the disease progression varied from rapid (as in families 3, 4, and 9–11) to a slow deterioration (as in the oldest persons from families 1, 2, and 8). Similar to the clinical presentation of the defective mitochondrial dynamics genes, bi-allelic *LETM1* variants were associated with an infantile-onset neurodegenerative disorder with a complex phenotype as described for *DNM1L*/*DRP1* (Dynamin 1 like [MIM: 603850]), *OPA1* (OPA1 mitochondrial dynamin-like GTPase [MIM: 605290]), *OPA3* (Outer mitochondrial membrane lipid metabolism regulator OPA3 [MIM: 606580]), *MFF* (Mitochondrial fission factor [MIM: 614785]), and *MSTO1* (Misato mitochondrial distribution and morphology regulator 1 [MIM: 617619]).^53^, ^54^, ^55^, ^56^ The shared phenotype mainly included global developmental delay, regression, and neurosensory impairment combined with neuromuscular symptoms, cerebellar ataxia, seizures, and early mortality. Akin to defects in *OPA3*, 3-methylglutaconic aciduria was a frequent finding in the subjects with bi-allelic *LETM1* variants.^57^ Bilateral cataracts and facial dysmorphism observed in the present *LETM1* cohort have also been reported in individuals with defective *OPA3* and *MSTO1*, respectively.^55^^,^^56^

All persons with RCE analysis results in the present study showed defects in the OXPHOS system suggesting that *LETM1* defects can affect the mitochondrial ability to generate ATP. This in turn might have mimicked the clinical presentation of OXPHOS MD. Therefore, distinguishing the *LETM1* phenotype from OXPHOS MD or the aforementioned diseases of mitochondrial dynamics can be challenging without the help of genetic testing, particularly in affected individuals with a rapidly progressive disease course.

### The phenotype of defective *LETM1* and WHS

Monoallelic *LETM1* deletion has been suggested to be responsible for epilepsy and neuromuscular features in WHS.^5^^,^^19^^,^^21^^,^^58^ Indeed, the current *LETM1* cohort presented with hypotonia and epilepsy. Additionally, though, persons with bi-allelic *LETM1* variants showed a milder spectrum of WHS signs that has not been previously ascribed to the *LETM1* deletion. These included thin habitus, low set ears, microcephaly, micrognathia, and low body weight.^59^^,^^60^ It has been previously speculated that the most probable cause of growth deficiency, microcephaly, and the characteristic facial features in WHS is due to haploinsufficiency of *WHSC1*, a region located far from *LETM1.*^61^ The expression of mild non-neurological symptoms of WHS in our cohort could be due to either putative interaction between *LETM1* and *WHSC1* or other undiscovered mechanisms, including those intrinsically caused by *LETM1* deficiencies.

We have observed some degree of clinical overlap between the presentation of defective *LETM1* and small interstitial deletions in WHS presenting with a milder phenotype. The latter presents with a variable degree of growth and neurodevelopmental delay, microcephaly, thin faces with dysmorphic features, intellectual disability, language impairment, and seizures.^62^, ^63^, ^64^ Interestingly, persons with small 4p16.3 deletions encompassing *LETM1* suggested that *LETM1* might not be responsible for seizures in WHS as some individuals with *LETM1* deletion did not have seizures by the age of 4 and 9 years, whereas persons with preserved WSHCR-2 including *LETM1* developed seizures.^64^ Previous retrospective analysis suggests that several other genes in the terminal 4p region might potentially be involved in seizures in WHS.^5^

Clinical features including lactic acidosis, diabetes, cataract, neuropathy, and proximal myopathy combined with cerebellar ataxia, progressive spastic-ataxic gait, hyperkinetic movement disorders, and pontine/cerebellar atrophy were among the signs of the defective *LETM1* phenotype that are not typical of WHS; instead, they are more typical of archetypal mitochondrial disorders.

Although there have been a handful of reports on microdeletions in WHS describing genotype-phenotype correlations, the association between the specific symptoms of WHS and *LETM1* remains to be fully determined. To understand the full contribution of *LETM1* in WHS cases, further studies would be needed to investigate which phenotypes of WHS can be restored by the re-expression of *LETM1*. Apart from this, the identification of phenotypes that were consistent with both *LETM1* haploinsufficiency in WHS and *LETM1* bi-allelic variants will advance our understanding of the contribution of *LETM1* in WHS.

### Genotype-phenotype correlation of bi-allelic *LETM1* variants

The general distribution of the missense and frameshift variants to the highly conserved LETM domain and the C-terminal coiled coils, together with their comparable deleterious effects on mitochondrial morphology and KHE function, support the correlation of mitochondrial morphologic defects and imbalanced cation homeostasis. A previous variant analysis of the LETM domain found that Asp359 or the triple combination of Arg382, Gly383, and Met384 is necessary for the organization of cristae structure and growth complementation of *S. cerevisiae letm1*Δ strains.^65^ The missense variant c.1072G>A (p.Asp358Asn) identified here in family 5, which impaired mitochondrial morphology and KHE activity, is adjacent to Asp359. Based on cell-free data showing that the reconstituted LETM domain was sufficient to induce cristae invagination, Nakamura et al.^65^ concluded that cristae disorganization due to the single or triple variant occurred independently of ion homeostasis. Our findings are not in contradiction but propose that a regulatory contribution to cristae architecture by the LETM domain may depend on the swelling state of mitochondria in the cellular context.

Given the growing consensus that the hallmark of LETM1 deficiency is mitochondrial cation imbalance, we used yeast as a model organism to analyze mitochondrial KHE activity of *LETM1* variants from affected individuals and *LETM1* variants not associated with the disease. Based on the results, we propose that light-scattering experiments that capture mitochondrial volume status and kinetics of K^+^/H^+^ exchange are useful to predict the pathogenic potential of *LETM1* variants (Figure S5).

Linking clinical features with *in vitro* data, we found that fibroblasts expressing *LETM1* variants c.[878T>A; 2094del], p.[Ile293Asn; Asp699Metfs13^∗^], which were identified in the individuals F1:S1 and F1:S2 affected with epilepsy, neurosensory deficiencies, and diabetes, displayed mitochondria with disturbed morphology and membrane potential, reduced LETM1 levels, and a severe decrease in respiratory proteins of CI and CIV. Ectopic expression of the variants in yeast marginally rescued mitochondrial KHE activity. Persons harboring the variant c.2071−9C>G (p.Val691fs4^∗^) (F10) showed rapid clinical progression and died before reaching 1 year of age. Fibroblasts from this person displayed high LETM1 protein levels, indicating that the pathogenic variant and not the lack of protein was associated with the severe phenotypes. Ectopic expression of this variant failed to rescue wild-type KHE activity. The abundance of this non-functional *LETM1* variant suggests that it likely escaped the nonsense-mediated decay as the gained stop codon falls into the last exon.^66^ The variant c.898C>T (p.Pro300Ser) was identified in family 11 leading to a severe early infantile disease in the homozygous state. Fibroblasts and muscle lysates from those individuals showed reduced CI and CIV proteins. Drastic growth defects and lack of KHE activity were induced by this variant in yeast, which could somehow explain the severe clinical conditions caused by this variant. *LETM1* c.2220G>C (p.^∗^740Tyrext26) was identified in several subjects from F2 and F7 with developmental delay, walking difficulties, and seizures. Fibroblasts from F2:S1 exhibited swollen and fragmented mitochondria and hardly detectable LETM1 protein levels. Ectopic expression in yeast displayed somewhat reduced LETM1 protein levels and poorly improved KHE activity. Since the KHE uses the proton gradient generated by the respiratory chain to drive K^+^ flux, and LETM1 is likely involved in the insertion of mitochondrial encoded OXPHOS proteins into the membrane, it is surprising that the reduction of this LETM1 variant did not correlate with decreased OXPHOS components. There are several possible explanations for this. The OXPHOS damages could be secondary to LETM1 deficiency, the OXPHOS components (although not reduced) may not assemble as efficiently, or genetic compensatory mechanisms are involved. The affected individual carrying the homozygous variant *LETM1* c.1072G>A (p.Asp358Asn) (F5) presented defects in neurosensory functions and type 3 diabetes. We found severely impaired proliferation of F5-derived fibroblasts. Similarly, yeast growth was also repressed by this variant, and mitochondrial KHE activity could not be restored. Compared to the other variants, c.1072G>A (p.Asp358Asn) had the most deleterious effects on mitochondrial morphology, cell proliferation, and KHE activity, predicting this variant to have the most severe consequences. However, the viable state of the affected individual also here raises the possibility of a potential genetic compensatory background. In this respect, increased mtDNA copy number—often considered as an efficient way to overcome OXPHOS deficiencies in diseases and aging^67^—or elevated citrate synthase activity found in muscle specimens may indicate such a compensatory pathway (Figure 4C). Further examination will be required to clarify molecular compensatory mechanisms.

Other *LETM1* variants were analyzed in yeast, as fibroblasts from affected individuals were not available. Yeast data revealed poor complementation of *S. cerevisiae letm1*Δ by human *LETM1* c.754–756del (p.Lys252del), a variant identified in affected individuals with a neurological, neuromuscular, and craniofacial presentation, rapid progression, and eventually death (F3). Ectopic expression of *LETM1* c.881G>A (p.Arg294Gln), identified in persons with variable disease progression (F4, F8) but similar neuromuscular deficiencies, was not able to restore the activity of the mitochondrial KHE, since the swelling traces revealed continuous but very slow kinetics indicating minimal KHE activity per time unit, thus suggesting leaky mitochondrial membranes. Yeast growth was also impaired by overexpression of this variant. Phenotypic data were rather consistent with severe clinical presentation and early demise in F4.

The affected individual from family 9 was homozygous for the *LETM1* variant c.1139G>C (p.Arg380Pro) and presented respiratory insufficiency, epileptic encephalopathy, neuromuscular disorder, and rapid disease progression. The missense variant is located in the middle of the LETM domain, in proximity to the three highly conserved amino acid residues (Arg382, Gly383, Met384) described in Nakamura et al.^65^ (Figure 2B), supporting an essential functional role of the LETM stretch between residues 380 and 384.

### LETM1 role in cation homeostasis and neurodegenerative phenotype of the cohort

Among the mitochondrial EF-hand-containing proteins, LETM1 has been identified as essential across several cell lines in genome-wide essentiality screens.^68^^,^^69^ Functionally, LETM1 is required for maintaining mitochondrial homeostasis of K^+^ and was considered an essential component of the KHE. After LETM1 was identified in a genomic *Drosophila* RNAi screen for mitochondrial Ca^+^/H^+^ exchanger (CHX), it has been suggested to catalyze the exchange of Ca^2+^ against H^+^ in both directions in a ruthenium red-sensitive pattern,^70^ which is difficult to reconcile with the CHX, and has been implicated in the pathogenesis of Parkinson’s disease through interaction with *PINK1* (PTEN-induced kinase 1 [MIM: 608309]).^71^ The mitochondrial CHX is part of the mitochondrial Ca^2+^ release system, which compensates for electrophoretic mitochondrial Ca^2+^ uptake mainly through H^+^- or Na^+^-dependent Ca^2+^ extrusion. While the role of LETM1 as a mitochondrial KHE or CHX has remained controversial, deregulation of the mitochondrial KHE has been shown to affect mitochondrial Ca^2+^ buffering by impacting the Na^+^-dependent Ca^2+^ release pathway.^72^ Proper maintenance of mitochondrial Ca^2+^ levels is critical to neurons, synaptic function, and neurodevelopment with mishandled mitochondrial Ca^2+^ levels posing a risk of synaptopathies. In turn, synaptopathies may be a harbinger of neurodegenerative disorders.^73^ The neurodegenerative phenotype observed in the present *LETM1* cohort could partially be explained by impaired mitochondrial Ca^2+^ buffering and ensuing glutamate excitotoxicity, generation of reactive oxygen species, and apoptosis.^74^ Consistent with previous studies,^13^^,^^14^^,^^50^^,^^75^ exposure to nigericin or ketone bodies improved the mitochondrial morphological phenotypes of fibroblasts from affected individuals, supporting the link between *LETM1* variant and impaired cation homeostasis. While nigericin enables K^+^-H^+^ exchange and prevents accumulation of matrix K^+^, ketone bodies may bypass the deficient Ca^2+^-dependent catalytic function of the pyruvate dehydrogenase.

Unlike in yeast, LETM1 orthologs of more complex organisms possess EF-hands, which may implicate LETM1 in Ca^2+^ sensing or regulation.^76^ Focusing on K^+^ analysis using yeast, we did not investigate the impact of the reported bi-allelic *LETM1* variants on the mitochondrial Ca^2+^ homeostasis. This would need to be investigated in further studies as it might have future therapeutic implications.^73^

Collectively, our results demonstrate that bi-allelic pathogenic *LETM1* variants are associated with defective mitochondrial K^+^ efflux, swollen mitochondrial matrix structures, and a reduction in proteins levels and activity of the electron transfer chain. The former highlights the implication of perturbed mitochondrial osmoregulation caused by bi-allelic *LETM1* variants in neurological and mitochondrial pathologies. Data showing that mitochondrial KHE activity is maintained above a functional threshold in non-pathogenic variants suggest that such functional yeast assays could be implemented to routinely determine the pathogenicity of a variant. While the beneficial effect of nigericin strengthened the link to KHE defects, that of ketone bodies, consistent with Durigon et al.,^14^ supports the promising therapeutic role of ketogenic-based diets.

### Data and code availability

The accession numbers for the genetic variants reported in this paper are ClinVar: SCV001981656, SCV001981657, SCV001981658, SCV001981659, SCV001981660, SCV001981661, SCV001981662, SCV001981663, SCV001981664, and SCV001981665.

## Web resources

Brain RNA-seq, http://www.brainrnaseq.org/

ClinVar, https://www.ncbi.nlm.nih.gov/clinvar/

ClinVar (summary of submissions), https://www.ncbi.nlm.nih.gov/clinvar/submitters/26957

Database of Genome Variants, http://dgv.tcag.ca/

Ensemble, https://www.ensembl.org/index.html

FlyBase, http://flybase.org/

GeneMatcher, https://genematcher.org/

gnomAD, https://gnomad.broadinstitute.org/

Iranome, http://www.iranome.ir/

OMIM, https://www.omim.org/

TOPMed, https://bravo.sph.umich.edu/freeze8/hg38/gene/snv/LETM1

UKBB, https://www.ukbiobank.ac.uk/

Uniprot, https://www.uniprot.org/

Varnomen, http://varnomen.hgvs.org/

Varsome, https://varsome.com/

## Supplemental information

Document S1. Supplemental note, Figures S1–S5, Table S2, supplemental material and methods, and supplemental acknowledgments

Table S1. Extended version of Table 2

Document S2. Article plus supplemental information
