# Supplementary material for: Bi-allelic LETM1 variants perturb mitochondrial ion homeostasis leading to a clinical spectrum with predominant nervous system involvement
Source: Am J Hum Genet. 2022 Sep 1;109(9):1692–712. doi: 10.1016/j.ajhg.2022.07.007 (PMC9502063; doi:10.1016/j.ajhg.2022.07.007)
Supplement: Document S1. Supplemental note, Figures S1–S5, Table S2, — supplemental material and methods, and supplemental acknowledgments [file mmc1.pdf]

The American Journal of Human Genetics, Volume *109*

Supplemental information

Bi-allelic *LETM1* variants perturb mitochondrial ion homeostasis leading to a clinical spectrum with predominant nervous system involvement

Rauan Kaiyrzhanov, Sami E.M. Mohammed, Reza Maroofian, Ralf A. Husain, Alessia Catania, Alessandra Torraco, Ahmad Alahmad, Marina Dutra-Clarke, Sabine Grønborg, Annapurna Sudarsanam, Julie Vogt, Filippo Arrigoni, Julia Baptista, Shahzad Haider, René G. Feichtinger, Paolo Bernardi, Alessandra Zulian, Mirjana Gusic, Stephanie Efthymiou, Renkui Bai, Farah Bibi, Alejandro Horga, Julian A. Martinez-Agosto, Amanda Lam, Andreea Manole, Diego-Perez Rodriguez, Romina Durigon, Angela Pyle, Buthaina Albash, Carlo Dionisi-Vici, David Murphy, Diego Martinelli, Enrico Bugiardini, Katrina Allis, Costanza Lamperti, Siegfried Reipert, Lotte Risom, Lucia Laugwitz, Michela Di Nottia, Robert McFarland, Laura Vilarinho, Michael Hanna, Holger Prokisch, Johannes A. Mayr, Enrico Silvio Bertini, Daniele Ghezzi, Elsebet Østergaard, Saskia B. Wortmann, Rosalba Carrozzo, Tobias B. Haack, Robert W. Taylor, Antonella Spinazzola, Karin Nowikovsky, and Henry Houlden

Supplemental data

Supplemental note: case reports

Family 1

This family presents with two affected siblings. The index case (F1:S1) is a 35-year-old female born full-term following uneventful pregnancy and delivery to non-consanguineous healthy British parents.Her neonatal period was unremarkable with normal birth weight, height, and occipitofrontal circumference. The parents have started expressing concerns from about one year of age. She has never crawled and always been unsteady when standing. She has never acquired speech and always had difficulty swallowing. She walked at around 17 months of age, and although she was delayed in walking, she did learn to run and did ballet until she was around the age of 6 or 7 years. From that time, there seemed to be a deterioration in her balance overall, although she did fluctuate from time to time. Treatment with acetazolamide did seem to help with less period of fluctuation. Continuous deterioration in ataxia started from age 7 years and had led to the loss of independent ambulation by age 13 years. Hearing loss was confirmed at the age of 2 years but in retrospect, her parents feel that she probably never heard. Visual problems were noted at around the age of 5 years, and she is now registered with partially sighted. Learning difficulties became apparent when she started at a school with a unit for hearing impairment and around the same age, intellectual disability was diagnosed. At 14 years old generalized tonic-clonic seizures started with the maximum frequency of one per two years. Seizures were well-controlled by carbamazepine. At age 18 years, she developed insulin-dependent diabetes mellitus. No behavioral symptoms and autism were reported. She did not develop cardiomyopathy and her electrocardiography together with echocardiography were normal. Her disease course has been slowly progressive.

With suspicion of mitochondrial disease as a cause of her symptoms, she underwent various investigations. Visual evoked potentials showed post retinal dysfunction and electroretinogram was normal. Nerve conduction studies showed signs of axonal sensory-motor neuropathy and electromyography was revealing for denervation and re-innervation processes. Muscle biopsy, white cell enzymes, very-long-chain fatty acids, and CSF lactate were normal. Mitochondrial respiratory chain enzymes showed borderline-low activity of complex II+III and IV. Co-Q levels were slightly low (127, normal value 140-580) and alanine was slightly raised at 38 in CSF. Genetic tests for *SCA1, SCA2, SCA3, SCA6, SCA7, FRDA*, EA2, common *POLG* mutations, mtDNA rearrangements, common point mutations of mtDNA were negative. Plasma amino acids analysis showed increased alanine, glycine, and serine.

Upon recent examination at age 35 years, she had short stature (height 153 cm) and a small head circumference (50 cm). Her face was dysmorphic including a long thin face with a prominent nose, low sitting ears, and teeth abnormalities. Joint contractures at knees, elbows, and hands together with kyphoscoliosis were present. She was alert but non-verbal and could make only unintelligible sounds. The cranial nerves examination revealed bilateral optic atrophy, mild weakness of facial muscles, and severe bilateral sensorineural deafness. Her arms and legs were slim, and her feet and hands were cold with wasted muscles. In combination with reduced pinprick sensation, these findings suggested peripheral neuropathy. There was significant spasticity in the upper and lower limbs. She could make several steps with support and her gait was spastic-ataxic. Myoclonic jerks in limbs and facial muscles were observed. Muscle jerks precipitated by movement. Her movements generally were slow. A tremor in fingers when arms outstretched was also noticeable. Deep tendon reflexes were brisk at the knees and ankles with feet clonus and upgoing plantars bilaterally. Biceps tendon reflexes were present on the right side and reduced on the left. Supinator reflexes were also reduced bilaterally. Brain MRI showed severe pontine and cerebellar atrophy.

Affected brother (F1:S2)

This is a 25-year-old male born full-term following a pregnancy complicated by one kidney infection in his mother. He was born by normal vaginal delivery and his birth weight and length were normal. There were no concerns initially but, again, he was a late walker at 17 months. He did however crawl at around 9 months of age and did learn a few words. His parents feel that he was always different from his sister. Brain stem evoked potentials at six weeks of age were reported to be normal. His parents had concerns regarding his balance and felt that when he did learn to walk it was “like a little old man”. When it became apparent that he was unstable on his feet, further investigations confirmed that he had a bilateral hearing loss at age 18 months. He has been wearing hearing aids since age 5 years. An audiogram at 5 years old reported a profound high-frequency loss and a moderate-to-severe low-frequency loss. He had attended a school with a hearing unite there had been some improvement in speech. By age 5 years he was found to have impaired vision. The disease has had a slowly progressive course and he lost autonomous ambulation by age 6-7 years. Nevertheless, his symptoms have always been less severe than that of his sister. From the age of 9 years, he developed generalized tonic-clonic seizures that used to come in clusters 2-3 times every 2-3 months. The duration of seizures was around 2-3 minutes and they were well-controlled by antiepileptic medication. He did not express cardiac complications but recently diabetes was diagnosed. Muscle biopsy, EMG, NCS conducted at

age 5-6 were reported to be normal. Plasma amino acids analysis showed Increased serine, glycine, and homocysteine. Plasma FGF21 levels were within normal limits (740pg/ml).

Upon examination at age 25 years, he presented with an almost similar phenotype as his sister but at the milder end of the spectrum. He had a short stature with a small head circumference (height 152 cm, head circumference 52 cm). Mild dysmorphic facial features and joint contractures at knees, elbows, and hands were also present. He was intellectual disability, right sided esotropia, and bilaterally pale optic discs. He could say some words with dysarthric speech and some of his words were comprehensible. The rest of the neurological examination was similar to his sister's findings.

Family 2

The index case (F2:S1) is a 24-year-old man born full-term after uneventful pregnancy and delivery to consanguineous healthy parents of Pakistani origin. He has two unaffected sisters. His birth weight was 3.35 kg and no neonatal abnormalities were found. Normal development was reported until age 2½ years when he gradually lost language skills. After initial normal motor development, his motor function has regressed from 4 years of age along with his cognition. His disease slowly progressed and by age 5 years he manifested epileptic seizures that have been well controlled. The type of seizures included focal, generalized, and episodes of absences. Their frequency was 1-3 per year over the recent years, and they typically last for one minute. EEG at 18 years showed 3-4 Hz activity in the left fronto-pretemporal region as single potentials and short trains, with amplitude up to 150 microvolts, and a single 3-4 Hz irregular spike/wave paroxysm lasting 1 second. At age 6 years he was diagnosed with bilateral sensorineural deafness and hearing aids were fitted. He has gradually developed severe spasticity requiring bilateral Achilles' tendon surgery (extension) at age 12 years and 18 years. There were no signs of cardiomyopathy, and an ECG was normal at age 20 years. At age 15, his weight was 33 kg and his height was 170 cm.

As an adult, he has severe psychomotor retardation, optic atrophy (6/38 Cardiff at age 21 years) with nystagmus, bilateral cataracts combined with spasticity, hand tremor, and atrophy of small hand muscles. He does not speak and uses a wheelchair. He did not have dysmorphic features, scoliosis, diabetes, or behavioral problems. No cerebellar ataxia, hypotonia, myopathy, or neuropathy were reported. Screening of urine showed excretion of methylglutaconic acid. Brain MRI at age 4 was reported to be normal.

Family 3

This family had 3 affected siblings born to consanguineous Kuwaiti parents.

Index case (F3:S1)

The index case was a female born at term with a birth weight of 2.9 kg and her mother had gestational diabetes. Her Apgar scores were 8-1 and 9-5 and she was admitted to SCBU for overall assessment due to a family history of previous infant deaths. Neonatal echocardiography revealed mild ventricular hypertrophy, a small atrial septal defect (ASD), and a patent foramen ovale (PFO). Serum lactate (3.56 mmol/L) and CK-MB (9.7 ng/ml) were elevated. Repeat echocardiography showed 2 small ASDs, left ventricle hypertrophy that evolved to biventricular hypertrophy with a mild left ventricular outflow tract obstruction (LVOTO). At 2 months old, her blood tests showed normal readings for complete blood count, amino acids, ions, metabolites, carnitine, and acylcarnitine, while her serum lactate level was reduced (2.56mmol/L). At 4 months old, she was noted to have sweats during feeds. Her length and head circumference were above the 10th centile, she had nystagmus, blue sclera, and abnormal hearing but no ptosis was observed. Significant shoulder girdle and limb hypotonia were noted with no brisk deep tendon reflexes (DTRs). At 7 months old, echocardiography revealed pericardial effusion for which she was hospitalized. Her weight was on the 3rd centile and her head circumference and height were on the 10th centile. Hypotonia persisted but brisk DTRs were noted. At 9 months old, an MRI brain scan showed no significant findings other than a mild ventriculomegaly. At 1 year old, her mother noticed a reduced amount of urine and she became lethargic, drowsy, pale, with poor feeding, which later progressed to cyanosis and tachypnoea. She had tachycardia (210 beats per minute) with no measurable blood pressure for which she was put on a mechanical ventilator. At the same time, she was treated for metabolic acidosis. Echocardiography showed a hypertrophic dilated left ventricle with poor systolic function. She passed away 2 days later due to refractory cardiogenic shock and disseminated intravascular coagulation.

Affected older brother (F3:S2)

The proband's older affected brother was born at term with no neonatal problems. At 6 months old, his parents noticed him becoming floppy with difficulty feeding and failure to thrive. A gastrostomy was performed, and a feeding tube was fitted. By 7 months old, his development stopped, and he presented with hearing problems and nystagmus. Dysmorphic features and facies myopathica were noted along with reduced visual acuity. Lactate levels were repeatedly elevated, EMG reported myopathic changes, and a brain MRI scan showed non-specific changes. Muscle biopsy analyses reported histopathological changes associated with mitochondrial disorders, electron microscopy

reported normal mitochondrial structure, and respiratory chain enzyme activity was reduced in OXPHOS complexes I, II, III and IV. mtDNA depletion was suspected but DNA quantification analysis reported no mtDNA depletion.

He later presented with cyanosis, respiratory distress, tachycardia, and desaturation, which was suggestive of pneumonia, and was connected to a mechanical ventilator. Arterial blood gases showed respiratory acidosis with elevated serum lactate at 12.65 mmol/L. He had non-generalized edema with cold extremities, distended abdomen with abdominal wall edema. He was tested and was diagnosed with septic shock due to a Pseudomonas infection with CMV infection. This resulted in multiorgan failure including liver and renal failure. He became hypotensive and echocardiography showed left ventricular hypertrophy and ejection fraction was 50%. He became bradycardic and was resuscitated numerous times until he passed away soon after.

Affected older brother (F3:S3)

The proband's younger affected brother was a product of 36 weeks gestation with a birth weight of 2.7 kg. His Apgar scores were 7-1 and 8-5. His mother had gestational diabetes and was only placed on a diet. He was admitted to the Special care baby unit due to tachypnoea, grunting, and missed heartbeats and so was at the hospital for the first 2 weeks of his life. A systolic murmur was audible, but the heart ultrasound showed a thick myocardium with a first-degree heart block. Echocardiography showed hypertrophied left ventricle and repeat echocardiography showed the same presentation with good ventricular function. Repeat lactate level readings showed a sharp elevation (11 mmol/L) that gradually normalized (2.4 mmol/L). An elevation in pyruvate levels was also measured. At 50 days of life, he had a 2-day history of cough, poor feeding, vomiting, and inactivity. Lactate was elevated (4.1 mmol/L) but reduced upon repeating the read. At 4 months old, he developed respiratory distress, fever, a runny nose, and a cough. A throat swab identified a Candida infection. Lactate levels were elevated (3.57 mmol/L) and he was treated with IV fluids, nebulized salbutamol, ipratropium bromide, cefotaxime, and erythromycin. He was later noted to be developmentally delayed with his head circumference, length, and weight all measuring below the 3rd centile. He was alert with a pale complexion and had an extended posture in both his lower limbs. He was hypotonic with normal DTRs. At 5 months old, he presented with tachypnoea and cardiomegaly due to pericardial effusion identified by echocardiography. The effusion resolved after he was put on protein-based powder milk. A metabolic blood workup showed elevated creatine phosphokinase (251 U/L), elevated lactate (6.54 mmol/L), elevated lactate/pyruvate ratio (105.6), and elevated total carnitine (105 µmol/L). A follow-up test 2 weeks later showed reduced lactate levels (2.66 mmol/L)

with an elevated lactate/pyruvate ratio (111), elevated alkaline transferase (75 U/L), elevated CK-MB (10.5 μg/L), and elevated CPK (82 U/L).

At the age of 1 year, he developed progressive respiratory distress and was admitted to a hospital. Arterial blood gases showed metabolic acidosis and echocardiography showed a pericardial effusion which was resolved by pericardiocentesis. He again developed respiratory distress and metabolic acidosis and was intubated and managed for 3 days before being shifted to pressure-controlled ventilation mechanical ventilators for further management. His lactate level was 6.4 mmol/L and his chest X-ray showed bilateral haziness and cardiomegaly. Tracheal aspirate showed growth of Pseudomonas aeruginosa and Klebsiella pneumonia. A repeat tracheal aspirate 2 weeks later showed the presence of the Pseudomonas aeurginosa and Klebsiella pneumonia. ECG showed sinus bradycardia and he required a blood transfusion. On discharge, his lactate level was 2.9 mmol/L. The younger brother passed away at the age of 1 year and 3 months with no report on the cause of death.

Family 4

The index case (F4:S1) is an Egyptian boy born full-term to consanguineous first cousin parents after a pregnancy complicated with hypertension. His birth weight was 2.7 kg and the neonatal period was unremarkable. He manifested at 4 months of age with nystagmus, hypotonia, and myopia. His disease had moderate rates of progression. He sat autonomously at 9 months of age, started walking independently at 20 months, and his first words started at the age of 16 months. The onset of progressive bilateral deafness was noticed from age 18 months and hearing aids were fitted from age 22 months. At 18 months of age, he had one attack of febrile convulsions and it was repeated only once at age 8 years. By the age of 2.5 years, he was noticed to lose independent ambulation due. Around the age of 3 years old, he developed significant impairment of vision and bilateral cataracts. First echocardiography done at age of 3 years showed mild ventricular hypertrophy and minimal mid cavity obstruction with pericardial effusion. There was one episode of haematemesis. He used to be admitted to hospitals due to hypoglycemia and metabolic acidosis. On examination at age 3 years, he presented with myopia, nystagmus, bilateral sensorineural hearing loss, and had midfacial hypoplasia. The speech was delayed as he used only sounds. He was hypotonic with myopathy. He was not reported to have ataxia spasticity, or neuropathy, feeding difficulties, muscular atrophy, encephalopathy, and hyperreflexia were present. Intellectual disability, behavioral abnormalities, and autism were not documented as well. He did not have kyphoscoliosis and no signs of retinitis pigmentosa were found. A brain MRI scan showed a mild reduction in brain volume. Lactic acidosis was reported but only with attacks of hypoglycemia. His renal function tests, plasma, and urine amino

acids were found to be normal. There was a significant elevation of adipic acid in the urine organic acids test. He died at age 8 years and the cause of death was unavailable.

Family 5

The proband (F5:S1) is an 11-year-old male, born by cesarean section to healthy non-consanguineous parents from Chechnya after an uneventful twin pregnancy with a birth weight of 2.4 kg. At the age of about 7 months, unilateral nystagmus of the left eye was noticed, at about 12 months also of the right eye. Eventually, atrophy of the optic nerve was diagnosed with pronounced visual impairment. A hearing loss was noticed since the 5th year of life. No other significant developmental problems were noted in early childhood. He was a poor eater and noted to be nervous and at times aggressive.At first presentation with 8 years, he showed a failure to thrive (BMI 12.7; -2.5 z) and slight microcephaly (-2.5 z), furthermore hirsutism, nystagmus, visual and hearing impairment, reduced muscle mass, externally rotated feet, and motor coordination deficits. Brain MRI showed optic nerve and chiasm atrophy. At the age of 9 years, he was admitted to a local hospital because of exercise intolerance which had been noticed at running, cycling, and swimming, maximum walking distance of1 km. A treadmill ergometry (600 m, 6 km/h, slope 10 %) led to a heart rate increase from 105 to 180/min, breathing rate 58/min, drop of pH from 7.44 to 7.06, lactate increase from 3.1 to 20 mmol/l.Other metabolic laboratory investigations and cardiology examinations were unremarkable. A thorough investigation revealed the following results: electroencephalography with background slowing, learning disability, mildly increased lactate and alanine in blood and CSF, known visual and hearing impairment, no further organ involvement. A muscle biopsy was unremarkable histologically.Western blot analysis of mitochondrial markers in muscle showed a global reduction for complexes I-IV, whereas complex V was slightly increased. Measurement of respiratory chain enzymes displayed a combined defect of complexes I, III, and IV in muscle. Medication with ubiquinone was started. At 10 years of age, a routine laboratory control showed lactate 5.1 mmol/l, pH 7.32, and glucose 8.0 mmol/l. An increased HbA1C level and pathological oral glucose tolerance test led to the diagnosis of type 3 diabetes and eventually treatment with repaglinide was started. At the current age of 11 years, the patient is underweight and slightly microcephalic, has visual and hearing impairment, a mild intellectual disability, muscular atrophy, otherwise no further neurological issues. He uses glasses, a reading device, and hearing aids and attends a special needs school. His FGF21 was 861 pg/ml (normal value <200).

He has three healthy siblings including his twin brother. Another 6-year-old sister has a history of unexplained acute symptoms in early infancy, according to the parents she had to be resuscitated. In

the further course febrile convulsions, night spasms, reduced muscle mass, externally rotated feet, rectal bleeding leading to diagnosis and removal of tubular adenoma of the rectum, hirsutism, mild hepatomegaly, and iron deficiency anemia occurred. Currently, there is no obvious developmental delay. Blood gases, lactate, and alanine were unremarkable. Sanger sequencing confirmed the familiar LETM1 variant in a heterozygous state. The family history is otherwise unremarkable.

Family 6

The index case (F6:S1) is a 17 month old female born to non-consanguineous Mexican parents. She has one male sibling with a possible autism spectrum disorder. Her prenatal period was remarkable with maternal polyhydramnios, 3rd-trimester spotting, and cervical incompetence. She was born full-term with an occipitofrontal circumference of 36 cm, birth weight of 3.1kg, and birth length of 48cm with Apgar scores of 8 and 9. Her neonatal period was significant for apnea, poor feeding requiring G-tube, laryngomalacia, and two ventricular septal defects, one of which was spontaneously closed later. The disease manifested from birth with poor feeding. She had acquired motor milestones at a normal age: sat at 6 months old, independent walking started at the age of 16 months, and the first words have started from the age of 8 months. Although, some delay in intellectual functioning was reported. Her feeding difficulties have gradually resolved, and the disease course was reported to be static. Upon her examination at the age of 2 months, her head circumference was below 3rd centile, and shehad bitemporal narrowing, micrognathia, high arched palate, overriding posterior sutures, and Anterior Fontanelle Open and Flat. Her weight and height were above the 50th percentile. She had normal vision and hearing with no nystagmus, ptosis, or ophthalmoparesis. On neuromuscular examination, she displayed normal muscle tone with no signs of muscular atrophy or hyperkinetic movement disorders. She did not display clinical seizures, but her EEG was abnormal due to excessive sharp transients with no epileptiform activity. No signs of diabetes and kyphoscoliosis were found upon her examination at 2 months old. From laboratory investigations only plasma amino acids wereavailable with normal results. Brain MRI conducted at age 1 week showed mild vermian hypoplasia.

Family 7

This family presents with 2 affected male siblings (F7:S1 and F7:S2) both born full-term after unremarkable pregnancies to healthy consanguineous Pakistani parents. The older affected sibling is currently aged 15 years old. The disease manifested at 1.5 years old with muscular weakness and difficulty standing up. He had a mild developmental delay with the acquisition of gait and first words

by the age of 2 years. At this time parents noticed an unsteady gait. His disease has had moderate rates of progression leading to the loss of ambulation by the age of 5 years and regression of speech. From the age of 5 years, he has started experiencing one per day generalized tonic-clonic seizures. The seizures were well controlled. By 11 years old he displayed impaired vision. He has shown no signsof metabolic acidosis or respiratory distress. Upon examination, at age 15 years his was significantly underweight (below 3rd centile) with small head circumference (below 3rd centile), kyphoscoliosis, and no clear features of dysmorphism. He is non-verbal with intellectual disability, communication, and behavioral issues. On neurological examination, he had appendicular spasticity with brisk tendon reflexes, upgoing plantars, and muscle atrophy. Examination of the sensory organs was remarkable only for impaired vision with normal hearing. He had chronic constipation and sleep disturbances, which was one of the main medical issues reported by the parents. His liver and renal function tests were normal and neurophysiology studies showed mil neuropathic changes.

The phenotype of the younger affected brother, currently aged 8 years, is similar to the older brother. He manifested at age 2 years with difficulty standing and walking with consequent ataxic gait and later motor regression with loss of ambulation by age 5 years. His brain MRI showed T2WI/FLAIR T2WS/FLAIR hyperintensities in periventricular white matter of bilateral parietooccipital lobes on both sides. Severe optic nerve and chiasm atrophy.

Family 8

The index subject (F8:S1) is a 37-year-old Italian patient born from first-degree cousins. She presented at birth with dysmorphic features, namely micrognathia and low set ears, divergent strabismus, and bilateral ptosis. She acquired normal gait at 10 months of age; early during childhood she manifested with growth and psychomotor delay, speech disturbances associated with severe sensorineural hypoacusis and bilateral cataracts. She also presented progressive gait ataxia and limb incoordination, diffuse skeletal muscle hypotrophy and facial hyposthenia. She was diagnosed with combined pituitary hormone deficiency during later development to early menopause and consequently leadingto severe osteoporosis. First brain MRI at the age of 8 demonstrated cerebral cortical atrophy, predominantly vermian cerebellar hypoplasia, abnormal optic tracts small pituitary gland. Severe bilateral brainstem dysfunction was evident at auditory evoked potentials, while electromyography displayed myopathic features without neuropathy. A muscle biopsy was performed when she was 11years old. Histology and histoenzymatic analysis were consistent with neurogenic muscle atrophy and mitochondrial dysfunction as several ragged-red fibers and COX-negative fibers. Biochemistry revealed reduced activity of mitochondrial respiratory complexes apart from complex II.

Last examination available, at 37 years of age, displayed a severely worsened ataxic gait, dysarthria, incoordination, oculomotor abnormalities and nystagmus; low body mass index (BMI), associated with advanced generalized amyotrophy and reduced deep tendon reflexes was also documented. A complete ophthalmologic evaluation was remarkable with tight bilateral myosis and disclosed an eso-hypertropia and signs of bullous keratopathy. Basic neuropsychological tests revealed behavioural disturbances with emotional lability and panic attacks reported by parents, marked intellectual disability with executive dysfunctions and defective verbal denomination. Repeated panic attacks were also reported by parents. Her plasma FGF21 levels were significantly elevated 2554 (normal value 0 - 153 pg/ml). The gas-chromatographic plot, relative to the urine sample, showed an increasein the excretion of some intermediates of the cycle of tricarboxylic acids (succinic, fumaric, 2-ketoglutaric and aconitic) associated with an increase in acid excretion 3-methylglutaconic and 3- methylglutaric. In addition, an increased excretion of pyruvic acid was found together with a modest increase in 3-hydroxybutyric acid (both in the -iso and in the -n forms). Urine amino acid analysis revealed increased levels of aspartic, serine, and glycine.

Her father developed ataxic wide-based gait during his 50s with evidence of mild dysarthria, limb incoordination and mild dysdiadochokinesia and bradykinesia during a follow up period of 9 years. Anamnestic recollection revealed a history of anxiety, panic attacks and phobic behaviour treated with serotonin reuptake inhibitors and benzodiazepines. Last clinical examination was performed when he was 79 years old: gait had worsened over time requiring the use of a walking aid for short-medium distance walking; neurological examination also showed moderate limb apraxia and signs of mild neuropathy, with absent tendon reflexes and impaired vibratory sensation on lower limbs Defective attentive and executive functions and weakened visual memory were documented by cognitive tests. Muscle strength was normal. Brain MRI displayed a predominantly vermian moderate cerebellar atrophy and mild brain atrophy.

The mother and the older sister of the proband were healthy; no health problems in the paternal grandparents were reported.

Family 9

The affected child (F9:S1) is a 1 year and 2 months old girl born full-term after uneventful pregnancy to consanguineous Pakistani parents. She was noted to have a weak cry, stridulous breathing, and recurrent apneas starting a few hours after birth with an initially normal neurological examination and Apgar scores of 9-9-9. Starting from the first days after birth and progressing over the first 4 months

of life, she developed progressive central and peripheral hypotonia with a paucity of spontaneous movements with poor respiratory effort. She spent ~ 4 months in PICU with ventilator support. She failed extubations on multiple occasions and finally had a tracheostomy at ~ 3 months of life with long-term ventilatory support. She had a cardiac arrest secondary to an apneic episode with downtime of 4 min during a brief period of trial of extubation at ~ 2 months of age. Subsequent MRI did not show any hypoxic-ischemic element. She failed the newborn hearing test; subsequent repeat hearing tests were technically difficult as she was ventilated in PICU. These were suggestive of sensorineural hearing loss. There was mild left ventricular hypertension felt secondary to hypertension but no cardiac dysfunction. Refractory infantile spasms and myoclonic jerks started at around 7 months of age. There were hourly clusters of spasms at peak. Spasms reduced and did not completely settle with adrenocorticotropic hormone. EEG did not show any improvement with steroid therapy and continues to be abnormal in keeping with epileptic encephalopathy. The EEG conducted at 4 months old shows evidence of mild maturational lag expressed by an asymmetrical increase in posterior slow. The repeat EEG at 5 months old has significantly deteriorated compared to the previous recording. The occasional sharp transients seen previously are now replaced by frequent bilateral and independent epileptiform discharges. She was initially bottle-fed but later nasogastric tube-fed after the need for ventilator support. Currently, she has poor suck and swallows. The main medical problems reported by parents were hypotonia, poor respiratory effort, and epileptic encephalopathy.

At her last follow-up examination at age 1-year-old, she had a global developmental delay with some responsiveness to surroundings- touch, sounds, and visual stimuli. There was a full range of ocular movements with perioral myokymia and intermittent nystagmus. She had a normal ophthalmologic assessment with no evidence for optic atrophy. However, she did not reliably fix or follow objects. There was peripheral and central hypotonia with mild flexion contracture at wrists and knees. There were no clinical or EMG signs of peripheral neuropathy.

Regarding investigations, basic metabolic workup on serum and urine was unrevealing. She had transient mildly elevated CSF lactate with normal plasma lactate. Abdominal ultrasound showed a slightly enlarged liver with echogenic parenchyma and normal kidneys. There was borderline reduced complex IV and muscle biopsy was suggestive of neurogenic but also had quite a lot of glycogen. Brain MRI scans from days 11 and 15 of life were reported to be normal. CGH microarray, tests for congenital myasthenic syndrome, common mitochondrial mutations, and SMA gene were negative.

Family 10

This family presents with currently deceased 2 affected siblings (F10:S2 and F10:S3) and affected maternal aunt (F10:S1) all born to consanguineous Portuguese parents. Due to early infantile death, their clinical details were limited. Their available medical records reported early onset rapidly progressive disease with hypotonia, spasticity, seizures present in all affected, and additional deafness, elements of cerebellar ataxia, and poor feeding present in the affected aunt. The three cases also had raised urinary 3-MGA (F10:S1 268, F10:S2 "detectable, not quantified" and F10:S3 1363 mol/mmol creatinine, reference range < 20). Both the affected aunt and the younger sibling had Complex I deficiency in muscle (37 and 38%, respectively).

Family 11

This family had 2 affected siblings (F11:S1, F11:S2). Subject 1 (female) was the first child of non-consanguineous healthy parents (Figure 2). She was born at 40 weeks from spontaneous delivery after an uneventful pregnancy. She presented at birth with cataracts and muscular hypotonia and was admitted to the Bambino Gesù Children's Hospital at the age of 8 months for apneic spells and cyanosis during crying. Clinical examination at admission documented severe muscular hypotonia, nystagmus and convergent strabismus, bilateral cataracts and retinal hyperpigmentation, systolic murmur, hepatomegaly with lower liver margin at 2 cm from the costal arch. Biochemical investigations documented metabolic acidosis, hyperlactacidemia (6.5 µmol/L, nv<2.1), low levels of free carnitine (15 µmol/L; normal value >26) and increased esterified/free carnitine ratio (3; normal value <0.3), hypertransaminasemia (GOT/GPT 88/135 UI/L; normal value <40), with normal blood glucose, LDH and CPK levels. Urinary organic acids profile showed increased excretion of Krebs cycle metabolites. EKG and echocardiogram documented the presence of hypertrophic cardiomyopathy. Brain CT scan and MRI were negative. Severe sensorineural deafness was detected by brainstem auditory evoked potentials and audiometry. Visual evoked potentials documented an increased latency. Electroretinogram was severely abnormal for increased latency and reduced amplitude. The electrophysiological study of the peripheral nervous system disclosed an axonal-type motor-sensory polyneuropathy. Muscular biopsy documented the presence of a type I fiber predominance, with mild glycogen storage. No typical RRFs were detected, but histochemical staining for SDH showed a widespread markedly increase signal due to mitochondrial proliferation, more evident in some fibers, while reaction for Cytochrome C oxidase (COX). Biochemical assays of MRC activities documented a defect of COX (10.92 nmol/min/mg protein; nv:17.87+/-3.99) and NADH cytochrome c reductase (796.74 nmol/min/mg protein; 1396.91+/-376.87). Molecular genetic studies performed on mtDNA extracted from muscle tissue ruled out mtDNA deletions. The patient was dismissed at home under

carnitine therapy and with external acoustic prostheses. Two additional brain MRIs performed during follow up at 13 and 31 months documented brainstem and cerebellar vermis hypoplasia, ventriculomegaly and delayed myelination. At the last examination, at 5 years, the patient showed severe moderate psychomotor delay with visual defect and bilateral nystagmus. The child died at 6 years of heart failure.

Subject 2 (male), the younger brother of subject 1, was the fourth child of the couple. He was born at42 weeks from spontaneous delivery after a pregnancy complicated by oligohydramnios. Weight at birth was 2.95kg (<3rdpercentile). He presented at birth with hypotonia and respiratory distress and was treated with oxygen therapy. At age 6 days a megacolon was suspected. Rectal biopsy however was negative. He also suffered from sepsis, requiring antibiotics, and necrotic enterocolitis. He was then transferred to Bambino Gesù Children's Hospital at the age of 2 months. Clinical examination atthe admission documented muscular hypotonia and severe failure to thrive. Routine biochemical testsshowed metabolic acidosis (bicarbonates 17 meq/l, base excess -10), responsive to oral NAHCO3 supplementation, hyperlactacidemia (up to 9.6 µmol/l, normal value <2.1), mild increase of transaminases (GPT/GOT 162/139 UI/L.; normal value <40), with normal glucose, ALP, LDH and CPK levels. Metabolic investigations revealed mild generalized increase of plasma aminoacids and significant excretion of lactic and pyruvic acids, Krebs cycle metabolites (2-ketoglutaric, 2-etil-3- hydroxypropionic, and fumaric acid) and tiglylglycine at urinary organic acids profile. EKG was normal,but an echocardiogram documented the presence of mild left ventricular hypertrophic cardiomyopathy. Based on the clinical phenotype and the family history, a mitochondrial disease wassuspected, and skin and muscle biopsies were performed at 3.5 months. Despite bicarbonate therapy,his clinical conditions and acidosis progressively worsened, and he died at 4.5 months of age of heartfailure.

Sanger sequencing in the following genes *SCO1, SCO2, COX15, EFG1, EFG2, EFG3, SUCLA2; MTO1*, *DNAJC1,* did not display any mutations. Moreover, based on the clinical association of cataracts, mitochondrial myopathy and cardiomyopathy, Sengers syndrome was hypothesized, however, sequencing of coding regions of AGK revealed no pathogenic changes. The possibility of MEDGEL syndrome was excluded as well by normal molecular testing of SERAC1 gene. A targeted resequencing for 1381 genes encoding for mitochondrial proteins ("Mitoexome") was performed.

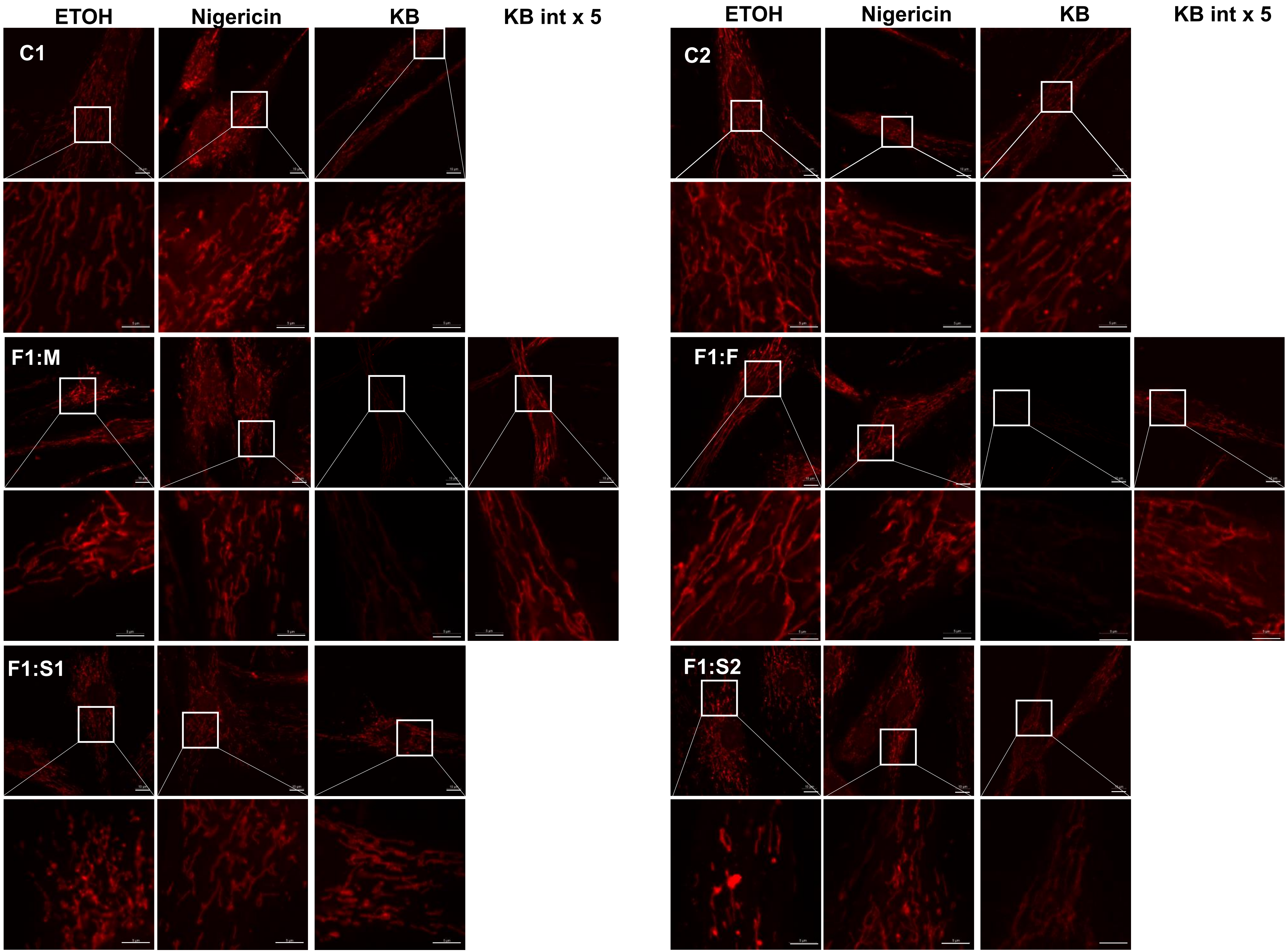

ETOH
Nigericin
KB
KB int x 5
ETOH
Nigericin
KB
KB int x 5
C1
C2
F1:M
F1:F
F1:S1
F1:S2

Figure S1A

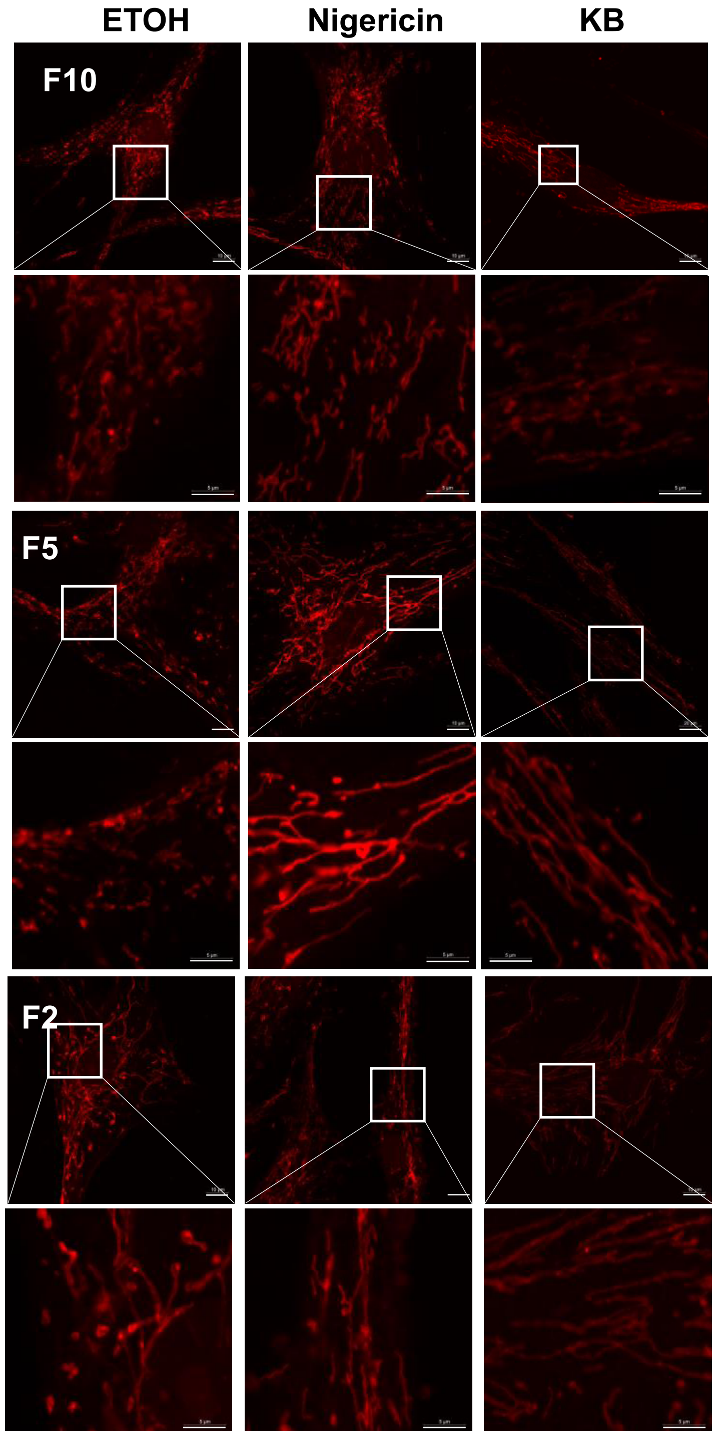

Figure S1A

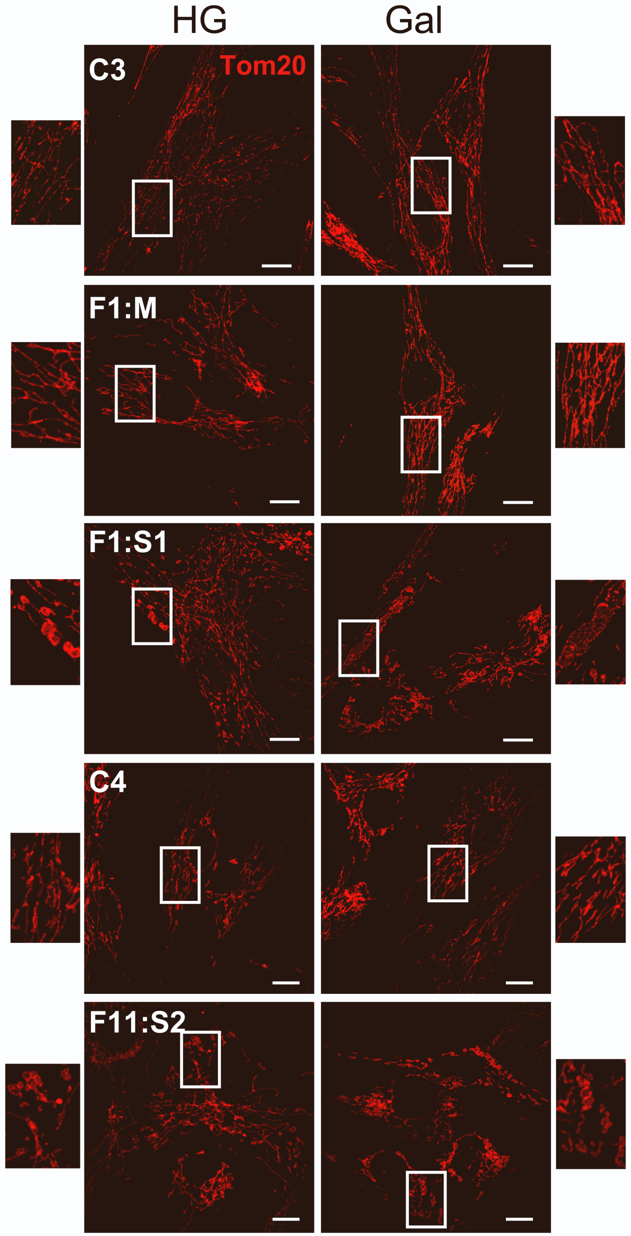

Figure S1B

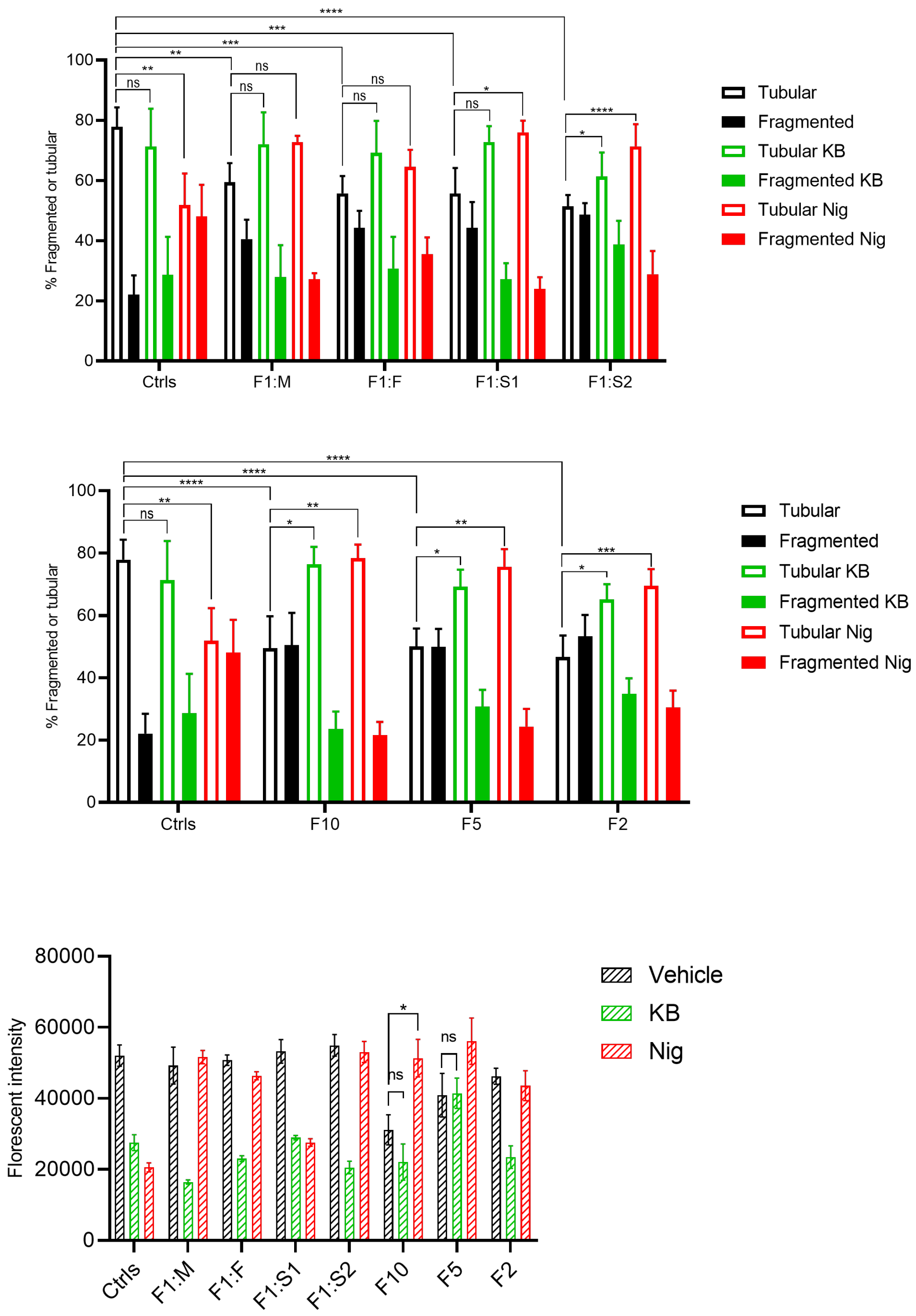
% Fragmented or tubular
Ctrls
F1:M
F1:F
F1:S1
F1:S2
Tubular
Fragmented
Tubular KB
Fragmented KB
Tubular Nig
Fragmented Nig
F10
F5
F2
Florescent intensity
Vehicle
KB
Nig

Figure S1C

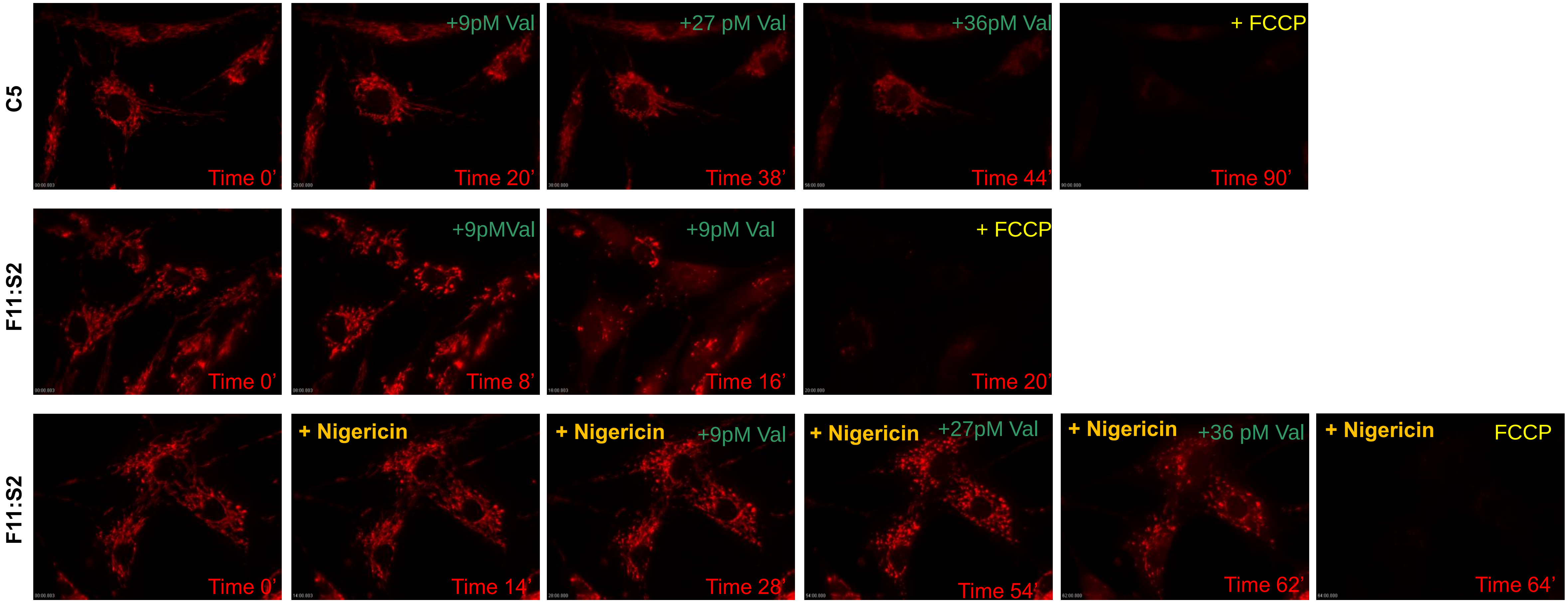
C5
Time 0'
+9pM Val
Time 20'
+27 pM Val
Time 38'
+36pM Val
Time 44'
+ FCCP
Time 90'
F11:S2
Time 0'
+9pMVal
Time 8'
+9pM Val
Time 16'
+ FCCP
Time 20'
F11:S2
Time 0'
+ Nigericin
Time 14'
+ Nigericin
+9pM Val
Time 28'
+ Nigericin
+27pM Val
Time 54'
+ Nigericin
+36 pM Val
Time 62'
+ Nigericin
FCCP
Time 64'

Figure S1D

Figure S1. Mitochondrial morphology in function of *LETM1* variants.

A) Rescue experiments with nigericin and ketone bodies

Summary of confocal images of fibroblasts exposed to vehicle (ETOH); nigericin or ketone bodies (KB)as indicated. Shown are representative images all taken at the laser intensity set at the same level forall samples, except at a 5 fold increase when indicated (KB intensity x 5) for F1:M and F1:F. Scale bars:overview images 10 μm, details: 5 μm

B) Detrimental effect of galactose on patient fibroblasts

Mitochondria of fibroblasts from control C3, C4, F1:M, F1:S1 and F11:S2 grown in medium supplemented with 25 mM glucose (HG) or 5 mM galactose (Gal) for at least 48 hours and immunolabeled with anti-TOM20 (red) antibody. Scale bar: 10 μm.

C) Quantitative comparison of mitochondrial morphotypes and membrane potential.

Upper and middle panels show the quantitative comparison between tubular versus fragmented shaped mitochondria given in percentage per cell under vehicle, ketone body and nigericin conditions, (Ctrls: mean of C1 and C2; the same control values were used in the upper and middle panels). Bottom panel shows the quantification of the membrane potential as a mean grey value per cell. N= 3 independent experiments, a minimum of 10 cells (with an average of 200 mitochondria per cells being analysed) is pooled per experiment. Vehicle: ethanol, Nig: nigericin, KB: ketone body, Statistics for morphotypes: mean ±SEM, non-parametric Kruskal-Wallis multiple comparisons test was performed of patient against healthy donor, *p<0.04, **p<0.004, ***p<0.0008, ****p<0.0001, non-significant ns>0.05. Statistics for lower panel vehicle F10 vs Ctrls ***p=0.0006; F10 vehicle vs nigericin F10 *p=0.0236, F1:S1 vehicle vs nigericin, and vehicle vs ketone body ***p<0.0002; F1:S2 vehicle vs ketone body ****p<0.0001; F2 vehicle vs ketone body ***p=0.0001, others n.s, non-parametric Kruskal-Wallis multiple comparisons test was performed.

D) Real time study of mitochondria from F11:S2

Time lapse recording of control (C5) and F11:S1 fibroblasts stained with TMRM (10 nM) and challengedwith increasing concentration of valinomycin in the absence or presence of nigericin over time. Complete depolarisation was achieved with FCCP (4 μM) at the end of the time course.

Figure S2

A

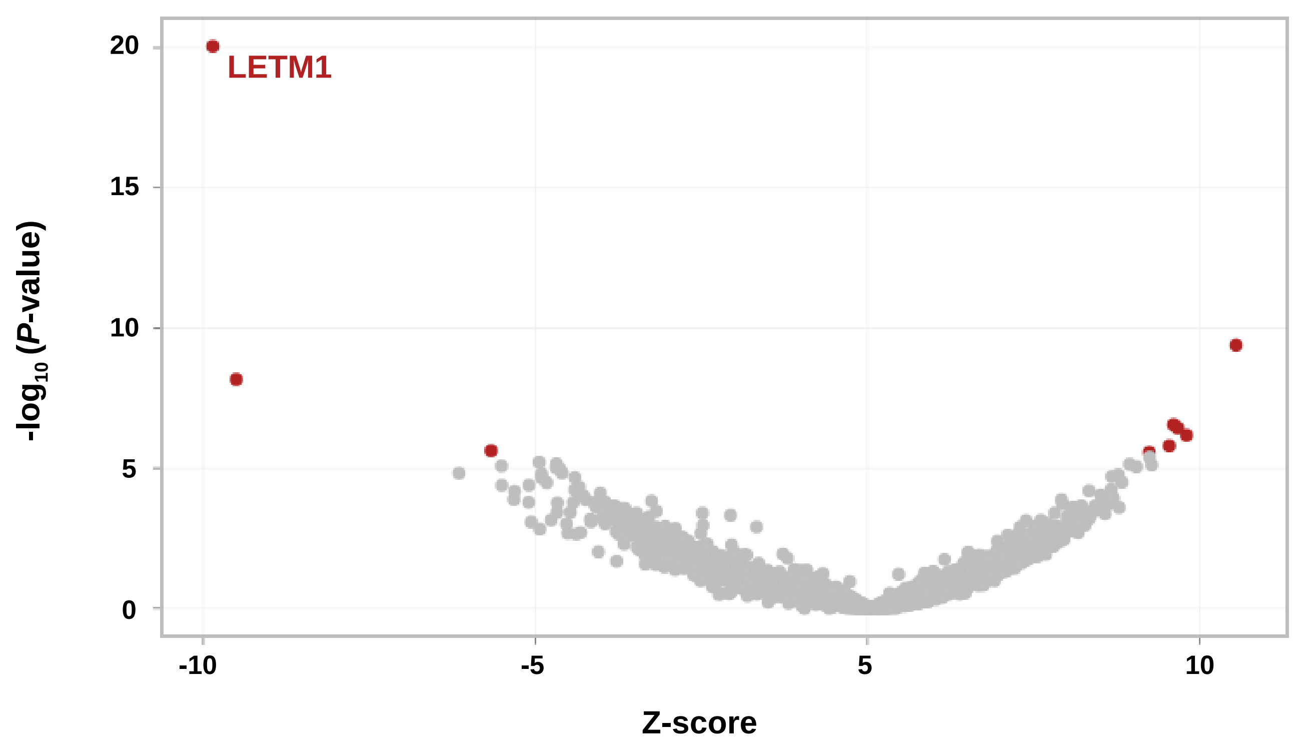

LETM1
20
15
10
5
0
-log10 (P-value)
-10
-5
5
10
Z-score

B

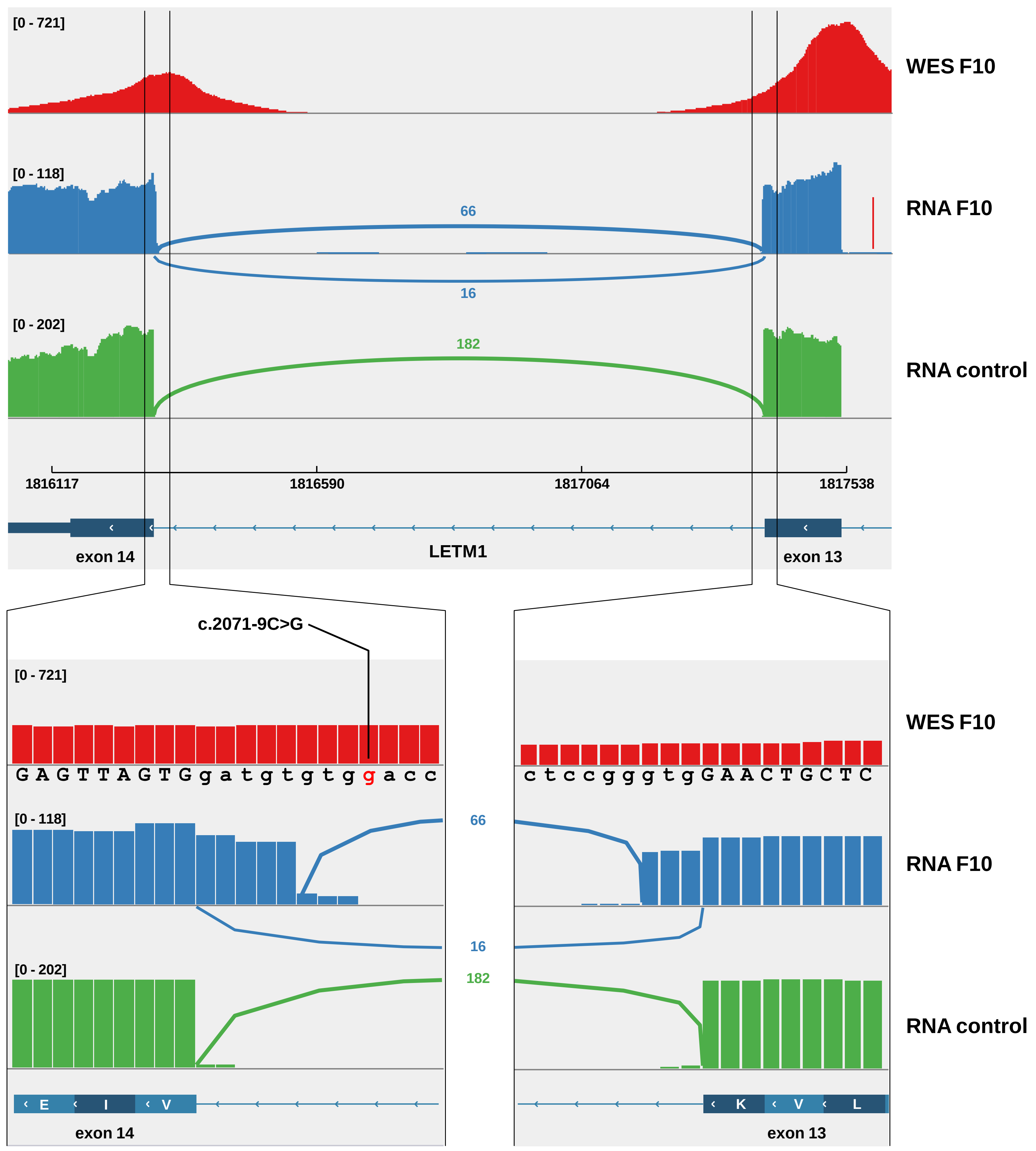

[0 - 721]
WES F10
[0 - 118]
66
16
RNA F10
[0 - 202]
182
RNA control
1816117
1816590
1817064
1817538
exon 14
LETM1
exon 13
c.2071-9C>G
[0 - 721]
WES F10
G A G T T A G T G g a t g t g t g g a c c
c t c c g g g t g G A A C T G C T C
[0 - 118]
66
RNA F10
16
[0 - 202]
182
RNA control
E
I
V
K
V
L
exon 14
exon 13

Figure S2. RNA-seq reveals aberrant expression and splicing of *LETM1* in F10

A. Volcano plot of F10 gene expression depicting the gene-level significance (-log10(P), y-axis) versus Z-score, with *LETM1* labeled among the expression outliers (red dots). B. *LETM1* Sashimi plot showing ES and RNA-seq of F10 (WES F10 in red and RNA F10 in blue, respectively) compared to the control RNA-seq (RNA control, in green), and the gene model of the RefSeq annotation below, with highlighted partial intron 13 retention caused by homozygous splice region variant c-2071-9C>G (C>G annotation based on the antisense strand) in F10, leading to a frameshift p.Val691*fs*Ter4*and nonsense-mediated decay.

Figure S3

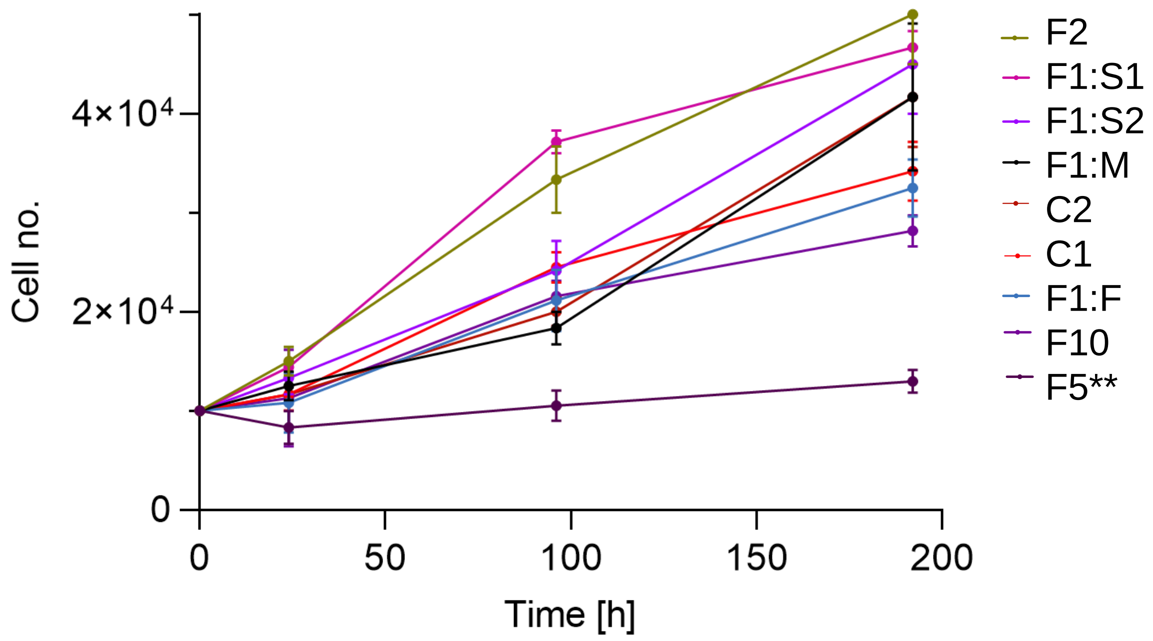

Cell no.
4×10⁴
2×10⁴
0
0
50
100
150
200
Time [h]
F2
F1:S1
F1:S2
F1:M
C2
C1
F1:F
F10
F5**

Figure S3. Different effects of *LETM1* variants on fibroblast proliferation

Overview of the proliferation of fibroblasts from controls (C1 and C2) and case (F1, F2, F5, F10). Cells were seeded at equal cell number and grown for 8 days, counted every second day as indicated, n=3 independent experiments. Statistics: ns>0,05, **p=0,007 using non-parametric Kruskal-Wallis multiple comparisons test comparing each of the cases against the controls.

Figure S4

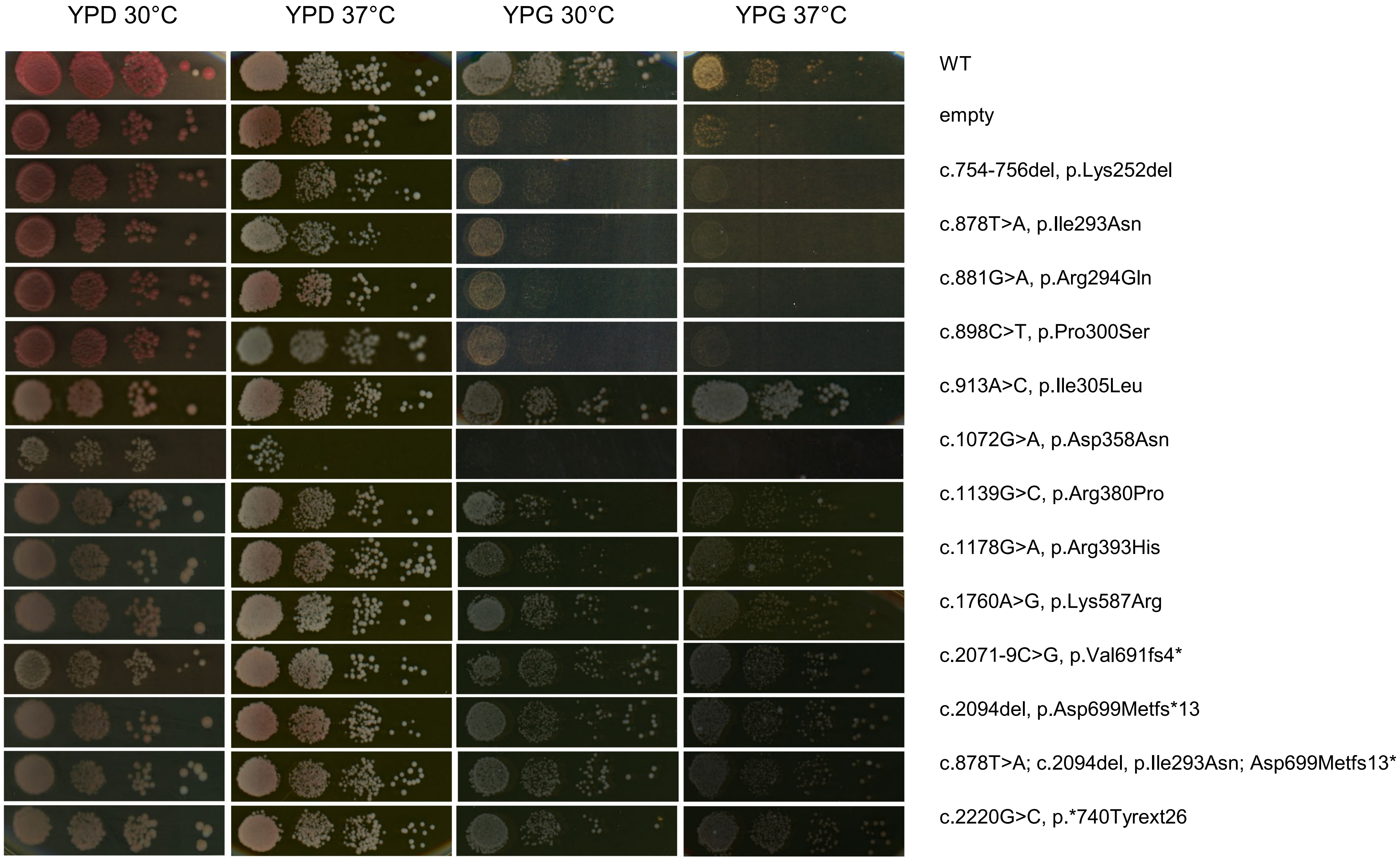
YPD 30°C
YPD 37°C
YPG 30°C
YPG 37°C
WT
empty
c.754-756del, p.Lys252del
c.878T>A, p.Ile293Asn
c.881G>A, p.Arg294Gln
c.898C>T, p.Pro300Ser
c.913A>C, p.Ile305Leu
c.1072G>A, p.Asp358Asn
c.1139G>C, p.Arg380Pro
c.1178G>A, p.Arg393His
c.1760A>G, p.Lys587Arg
c.2071-9C>G, p.Val691fs4*
c.2094del, p.Asp699Metfs*13
c.878T>A; c.2094del, p.Ile293Asn; Asp699Metfs13*
c.2220G>C, p.*740Tyrext26

Figure S4. Growth phenotype of *S. cerevisiae letm1Δ* expressing or not human *LETM1* wild-type or variants.

Serial dilutions of *S. cerevisiae letm1Δ* strains expressing pVT-103U empty (e) or carrying LETM1 WT, or LETM1 with the indicated variants were spotted onto fermentable (YPD) and non-fermentable (YPG) media and grown at 30°C or 37°C.

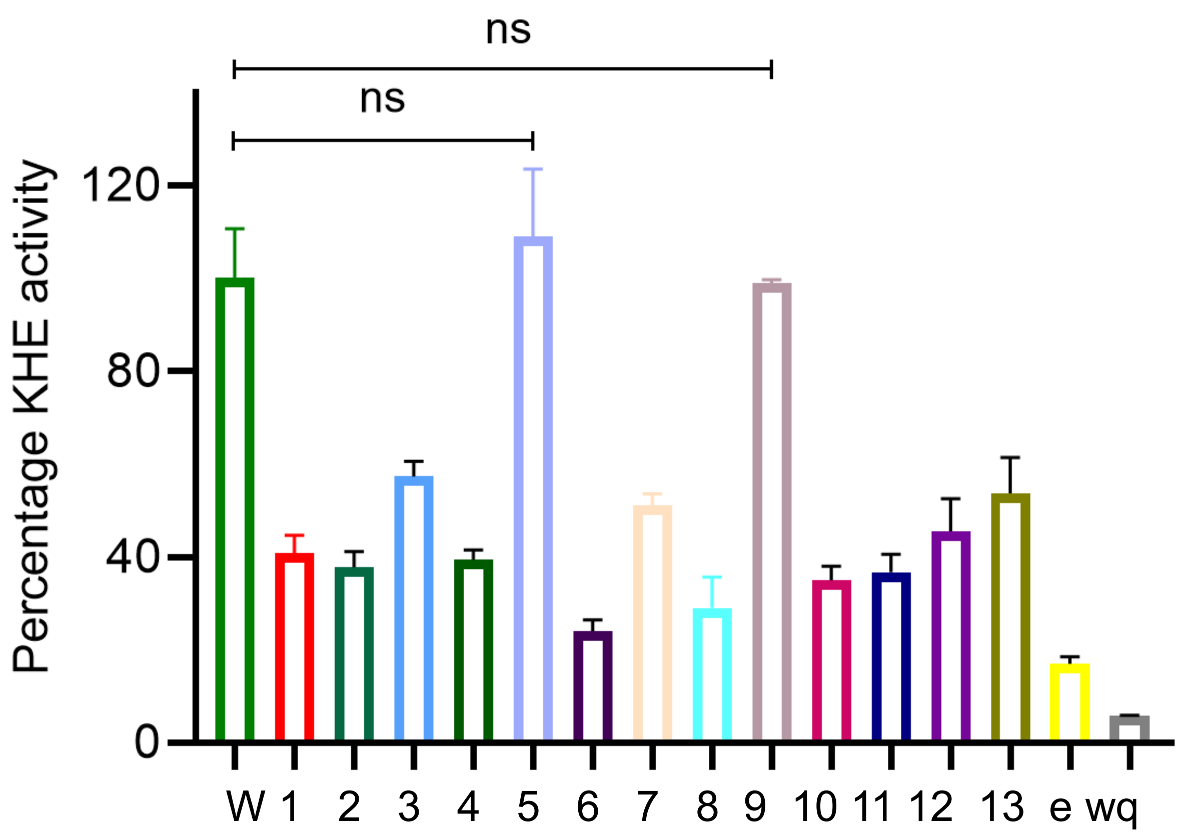

ns
ns
Percentage KHE activity
120
80
40
0
W 1 2 3 4 5 6 7 8 9 10 11 12 13 e wq

Figure S5

Figure S5. Evaluation of the relative KHE activity

Results shown in Figure 5 were evaluated in function of the swelling amplitudes, the swelling time between t=0 and t= 18 sec and the initial OD and are represented relatively to *S. cerevisiae ΔLETM1* expressing human *LETM1* wildtype. Statistics: non-pathogenic variants: n.s, all other ****p>0.0001 by one way ANOVA Dunnett´s multiple comparison test, means are ± SEM.

Table S1. Extended version of Table 2.

Available as a separate excel file.

Table S2. Enzyme investigations in muscle sample of Family 5

	Patient	Normal range
mtDNA/nDNA copy number	4367	887-2066
**Enzyme activities [nmol/min/mg protein]**		
Citrate synthase (CS)	349	134-260
Complex I	16	18-59
Complex I+III	23	18-45
Complex II	147	28-69
Complex II+III	71	16-43
Complex III	203	149-480
Cytochrome c oxidase	197	148-392
Complex V	230	60-223
Protein concentration [mg/ml]	3,02	
**Relative enzyme activities**		
Complex I/CS	0,05	0,11-0,26
Complex I+III/CS	0,07	0,11-0,24
Complex II/CS	0,42	0,14-0,43
Complex II+III/CS	0,20	0,10-0,29
Complex III/CS	0,58	0,75-2,32
Cytochrome c oxidase/CS	0,56	0,83-2,40
Complex V/CS	0,66	0,30-0,75

Supplemental Material and Methods

Skin and muscle biopsy and primary fibroblasts culture

Affected individuals F1:S1, F1:S2 and both parents (F1: Father (F1:F) and F1: Mother (F1:M)), F2:S1, F5:S1, F10:S1, and F11:S2 provided skin biopsies. Skin fibroblast cell lines were established in RPMI medium supplemented with 20% fetal bovine serum (FBS) and 1% penicillin/streptomycin (P/S) (Thermo Fisher Scientific) at 37° C in a humidified atmosphere with 5% CO_2. Cell lines were maintained in an incubator set to 37º C and 5% CO_2 and cultured in Dulbecco's modified Eagle medium DMEM with high glucose, GlutaMAX™, and pyruvate (ThermoFisher, cat# 31966-021), supplemented with 10% fetal bovine serum (FBS) and uridine 50 µg/ml (Sigma), or DMEM with glutamine, w/o glucose and w/o pyruvate (ThermoFisher, cat# 11966-025) supplemented with 0.3 mM β-hydroxybutyrate (ketone bodies (KB)) (Cayman Chemical, cat# 14148), 10% FBS, and 1% P/S for 24 hours. In some experiments, fibroblasts of F1 and F11 were grown in absence of uridine. The galactose regime employed glucose-free DMEM medium (Life Technologies) with the addition 5 mM of galactose and 10% dialyzed FBS. Cells were regularly screened for and confirmed free mycoplasma (MycoAlert Lonza kit cat# LT07-418 or Look Out Mycoplasma PCR Detection Kit (Sigma).

Muscle biopsies were provided by affected individuals F11:S1, F11:S2 and F5:S1. For F5, 50-100 mg tissue were transferred into sterile SIMPORT tubes (T405-2A) and 10-20-fold volume of SEKT buffer (250 mM saccharose, 2 mM EGTA, 40 mM KCl, 20 mM Tris; pH 7.4) was added. Samples were homogenized with an Ultra-Turrax at 20.500 U/min and the homogenate transferred to the glass Potter S- Homogenisator for 10 – 12 times homogenisation strokes on ice. The homogenate was transferred into a SIMPORT tube and centrifuged at 600 x g for 10 min at 4 °C. Aliquots were stored at -70°C. For F11 30 mg of muscle samples were homogenized in RIPA buffer (SIGMA) in the presence of 1X protease and phosphatase inhibitor cocktail (Thermo scientific). Subsequently, samples were centrifuged at 12000 rpm for 20 min at 4°C and supernatant was collected. Protein concentration was evaluated using BCA protein assay kit (Thermo scientific) and samples were read at λ 562 nm using an Agilent 8453 spectrophotometer.

Immunoblotting analysis

Total lysates of fibroblast cultures from F2, F10 and C2 were lysed in RIPA buffer (50 mM Tris-HCl pH 8.0, 150 mM NaCl, NP40 1%, 0.5% Sodium deoxycholate, 0.1% SDS, 1 mM EDTA with proteinase inhibitors (Sigma Aldrich, St. Louis, MS, USA). Protein lysates (15 µg/lane), as well as TCA-precipitated proteins from yeast total cell extracts (50 µg/lane) or cytoplasmic fractions, or isolated crude mitochondria (25 µg/lane) were separated by 12 or 15% SDS PAGE after determination of protein

concentration by BCA assay. After the transfer of proteins onto nitrocellulose membrane, membranes were blocked in 2% BSA-TBS-T and probed with the indicated antibodies (Table S2). Reactive bands were detected using SuperSignal West pico Chemiluminescent Substrate (Thermoscientific). Fibroblasts from F1, F11, C3, C4 were lysed in 0.1% n-dodecyl-D-maltoside (DDM, Sigma), 1% SDS, 50 U benzonase (Novagen), 1:50 (v/v) protease inhibitor cocktail (Roche), and 1:100 (v/v) phosphatase inhibitor (Cell Signaling). Fibroblasts lysates or 30 μg of muscle mitochondria were separated by 12% SDS PAGE, proteins were transferred onto polyvinylidene difluoride (PVDF) membrane. After blocking in 5% non-fat dry milk in PBS-T and probing with indicated antibodies (and Table S2), reactive bands were detected using Lite Ablot Extend Long Lasting Chemiluminescent Substrate (Euroclone, Pero (Mi), Italy). For mitochondria isolated by differential centrifugation and resuspended in SEKT buffer from F5 and C5 muscle, proteins were separated on 10% SDS PAGE and transferred onto nitrocellulose membrane, blocking reagent was from Roche in TBS-T, antibodies were as indicated in Table S2, and bands were detected using Lumi-Light PLUSPOD substrate (Roche).

Densitometry analysis was performed using Quantity One software (BioRad, Hercules, CA, USA) or Image Lab 6.1.0 software (Bio-Rad Laboratories, Inc.).

List of Antibodies used for immunoblotting:

Antibody	Company
LETM1 1: 1000	Abnova #H00003954-M03
LETM1 1: 1000	Aviva #OAAB12878
LETM1 1: 1000	Santa Cruz #sc-163013
LETM1 1:1000	Abnova H00003954-PW1
Porin 1: 1000	Invitrogen #459500
Cox2 1: 1000	Invitrogen #459150
Actin 1: 1000	Invitrogen #MA5-11869
Total OXPHOS cocktail 1:1000	Abcam #ab110411
COX4 1: 1000	Cell signaling #4850
NDUFA9 1:1000	Abcam #ab14713
NDUFB8 1:2000	Abcam #ab110242
MT-CO2 1:1000	Abcam #ab110258
SDHB 1:200	Abcam ab #14714
UQCRC2 1:1000	Abcam #ab14745
COXIV 1:1000	Abcam # ab14744
TOM20 1:600	Abcam #ab186735
DNA 1:250	Progen #AC-30-1
VINCULIN 1:5000	Abcam #ab14714
NDUFS4 1:1000	Sigma#WH0004734M1
SDHA 1:2000	Abcam ab #14715
UQCRC2 1:1500	Abcam #14745
MT-CO2 1:1000	Abcam #ab79393
ATP5F1A 1:2000	Abcam #ab14748
VDAC1 1:2000, or 1:3000	Abcam #ab14734, Abcam #ab15895

CS 1:3000	THP #NBP2-43648
GAPDH 1:5000	Trevigen #2275-PC-100
NDUFB8 rabbit polyclonal, 1:500	Abcam #ab192878
MT-CO1 mouse monoclonal, 1:1000	Abcam #ab14705
Peroxidase Mouse IgG	Thermo Fisher #31430
Peroxidase Rabbit IgG	Cell Signaling #7074p2
Peroxidase Goat IgG	Jackson ImmunoResearch Laboratories #305-035-003
Alexa Fluor 647Mouse IgG2a	Jackson ImmunoResearch, Laboratories # 115-607-186
DyLight 488 1:1000	Vector Laboratories #DI-2488
horseradish peroxidase-(HRP) 1:100)	EnVisionkit, Dako

Immunohistochemical staining

FFPE muscle tissue was cut with a microtome in 4 µm slides. The slides were heated for 1 h at 60°C. After rehydration (3 x 4 min xylol, 3 x 3 min isopropanol) slides were washed 3 x 3 min in ddH2O. Heat-induced antigen retrieval was performed in 1 mM EDTA, 0.05% Tween-20, pH 8 for 40 min at 95°C. Slides were allowed to cool down to room temperature. After washing 3 x 3 min in ddH2O and 3 x 3 min in PBS-T endogenous peroxidase activity was blocked with peroxidase block (DAKO envision kit) for 5 min. Slides were washed 3 x 3 min in PBS-T. Antibodies are listed in Table S2. All antibodies were diluted in Dako antibody diluent with background-reducing components (Dako, Glostrup, Denmark). Thereafter, the slides with incubated with the respective DAKO polymer (rabbit or mouse) for 1 h at RT. After washing 3 x 3 min in PBS-T DAB development was performed. Samples were rinsed 5 min in tap water to stop the reaction. Nuclei were stained with hemalaun for 3-5 min, briefly rinsed in 3% HCl-EtOH and blueing was done for 10 min in running tap water. After dehydration in isopropanol and xylol mounting was done in Histokit.

Spectrophotometric determination of the OXPHOS enzyme activity

Muscle 600 × g homogenates or isolated fibroblast mitochondria were used for determination of enzymatic activities of the OXPHOS complexes. Enzyme activities of the OXPHOS complexes were determined as previously described.[1] Rotenone-sensitive complex I activity was measured spectrophotometrically as NADH/decylubiquinone oxidoreductase at 340 nm. The activities of citrate synthase, complex IV (ferrocytochromec/oxygen oxidoreductase), and oligomycin-sensitive ATP synthase activity of the F1FO ATP synthase (complex V) were determined as previously described.[2] The reaction mixture for the ATPase activity measurement was treated for 10 s with an ultra-sonifier (Bio cell disruptor 250, Branson, Vienna, Austria). The reaction mixture for the measurement of

complex III activity contained 50 mM potassium phosphate buffer pH 7.8, 2 mM EDTA, 0.3 mM KCN, 100 µM cytochrome c, 200 µM reduced decyl-ubiquinol. The reaction was started by addition of the 600 x g homogenate. After 3–4 min the reaction was inhibited with 1 µM antimycin A. All spectrophotometric measurements (Uvicon 922, Kontron, Milan, Italy) were performed at 37°C.

Cell imaging

Fibroblasts from F1, F2, F5, F10, C1 and C2 were seeded onto 8 well dishes (Ibidi, cat#80826), stained with 50 nM Mitotracker Red CMXRos. Live staining was either fixed or followed by immunostaining (as described in Wilfinger et al.)[3] Image acquisition was done using a Zeiss laser confocal microscope LSM 880 and RFP channel, Plan-Apochromat 40x or 63x/1.40 Oil DIC M27 lens and pinhole 65 µm. In other cases, fibroblasts from F1 and F11 were grown on coverslips and immunostained as described in.[4] Samples were imaged either on a SP5 TCS Inverted Confocal Microscope (Leica Biosystem) using an immersion objective with a numerical aperture of 63x or 100x/1.4 Oil or on Nikon Ti Inverted Confocal Microscope using 60x immersion Oil objective. Z stack of red, green, and blue images using a step size of either 0.3 or 0.125 lm was acquired sequentially and merged using ImageJ. Laser power, gain and offset parameters were kept constant for each experiment. Any adjustments to brightness and contrast were applied linearly to all images in a comparison. For single immunostaining of the mitochondrial network with TOM20, samples from F11 were fixed with methanol:acetone (2:1) for 10 min in 5% BSA-PBS, visualized using the Alexa Fluor 647 secondary antibody, and images were acquired with a fluorescence-inverted microscope (Leica DMi8). An average of eight image planes wasobtained along the z-axis at 0.2 µm increments using the LASX 3.0.4 (Leica) software.

For transmission electron microscopy, fibroblasts were grown to semi-confluency on Aclar coverslips (Science Services; Munich). Primary fixation was done with 2.5% glutaraldehyde in 0.1 M sodium cacodylate buffer, pH 7.3 and postfixation with 1% OsO_4, dehydration in a series of ethanol, followed by sample infiltration in mixtures of acetone and Agar 100 resin. For final infiltration with pure resin, the Aclar coverslips were placed on glass slides with the cell layers facing up. After infiltration of the droplets of resin, Eppendorf tubes with their bottoms and lids cut off were placed above the samples. After an initial heat polymerization at 60°C, the Eppendorf tubes were filled with resin, and polymerization continued for two days. The Aclar coverslips were removed from the resin blocks. Thin sections (70-80 nm) oriented in parallel to the contact surface with the substratum were cut with an ultramicrotome Ultracut S (LEICA Microsystems, Vienna, Austria), mounted on 200 mesh copper grids, counterstained with neodymium(III)-acetate[5] for 50 min followed by lead citrate for 8 min and examined at 120 kV in a ZEISS Libra 120 transmission electron microscope. Images were acquired using

a bottom stage digital camera, TRS (4 megapixels), and ImageSp-professional software (Tröndle, Moorenweis, Germany).

RNA sequencing

Primary skin fibroblasts from patient S3 from Family 10 were used for RNA-sequencing as descirbed in Yepez et al.[6] In short, RNA was isolated from the cells using the RNeasy mini kit (Qiagen, Hilden, Germany) according to the manufacturer's protocol. RNA integrity number (RIN) was subsequently measured with the Agilent 2100 BioAnalyzer (RNA 6000 Nano Kit, Agilent Technologies, Santa Clara, CA, USA). Library preparation for the strand-specific RNA-sequencing was done according to the TruSeq Stranded mRNA Sample Prep LS Protocol (Illumina, San Diego, CA, USA). Library´s quality and quantity were determined with the Agilent 2100 BioAnalyzer and the Quant-iT PicoGreen dsDNA Assay Kit (Life Technologies, Carlsbad, CA, USA). Library was sequenced as 100 bp paired-end runs on Illumina HiSeq4000 platform. Reads from RNA-seq were demultiplexed and mapped with STAR v2.7.0a to the hg19 genome assembly.[7] Upon alignment, data were analyzed using the computational workflow DROP.[6]

Yeast serial dilution

All strains were grown overnight in selective media (SD-URA, synthetic dextrose media lacking uracil media following the protocol of Nowikovsky et al.[8] and diluted to final OD600 of 1; transferred 1:1, 1:10, 1:100 and 1:1000 to a microtiter plate, stamped onto YPD (2% glucose) and YPG (3% glycerol) plates and incubated at 30 or 37°C. YPD plates were incubated 2-4 days, YPG 30°C 4-6 days, and YPG 37°C 7-9 days.

Primers used for site-directed mutagenesis.

#	Variant	Forward primer 5´-3´	Reverse primer 5´-3´
1	c.754-756del	GAGCTTCGGGTCAAGCTG	CTTCAGCCTCTCCTCCTTG
2	c.878T>A	TTCCAGAAGAACCGGGAAACAG	AAACACAGAGAAGTCTTTGG
3	c.881G>A	CAGAAGATCCAGGAAACAGGG	GAAAAACACAGAGAAGTCTTTG
4	c.898C>T	AGGGGAGAGGTCCAGCAATGA	GTTTCCCGGATCTTCTGGAAAAAC
5	c.913A>C	CAATGAGGAACTCATGCGTTTTTCCAAATTATTTG	CTGGGCCTCTCCCCTGTT
6	c.1072G>A	CATAAAGGCAAACGACAAGCTGATTGC	GAGCGCAGCCGCATGGTA

7	c.1139G>C	GCAGCGTGTCCGGCACGAGGC	CTGCAGCTCCTTGACATTCAGG
8	c.1178G>A	ACGGAAGACCACCTGAGGGGTCAGC	GACGCCCAGGGCCCGCAT
9	c.1760A>G	CAGGAGATCAGGAAGGAACTTTC	CAAGTCCTCGCTGTAGTC
10	c.2071-9C>G(1)	GTAGGTGATTGAGCTGGTGGAC	ACACCTTGACGAGGTCGTCGAT
11	c.2094del(2)	GATGTTCACATCTCCACC	TCTTTGTCCACCAGCTCA
12	c.878T>A in LETM1-11 (3)	TTCCAGAAGAACCGGGAAACAG	AAACACAGAGAAGTCTTTGG
13	c.2220G>C(4)	GTCACCCTGGCAAGGGCCGTGAGGGCGATTGC TTTGTGGTTTACCTTTTACCCATACGATG	GGCACAGCAGGAGGACAGGTGCCC AGGCCAGTGGTTGTAGCTCTTCACCT CTGCGAC

(1) c.2071-9C>G leads to splice site defect characterized by retention of the first 8 nucleotides upstream of exon 14, the set of primers is used to insert the nucleotides. (2) c.2094del results in a frameshift and a premature introduction of a stop codon (at position 710). (3) To generatethe compound variant, LETM1 #2 was used as a template for introducing the second point variant (2094del). (4) c.2220G>C results in a longer protein of 765 aa.

Statistical analysis

All statistical analyses were done for independent experimental replicates using GraphPad (La Jolla, CA) Prism v8.0.1. for Windows. Tests and individual p values are indicated in the figure legends. Data are presented as mean ± SD unless other specified.

Supplemental acknowledgements

The families were collected as part of the SYNaPS Study Group collaboration funded by The Wellcome Trust and strategic award (Synaptopathies) funding (WT093205 MA and WT104033AIA). This research was conducted as part of the Queen Square Genomics group at University College London, supported by the National Institute for Health Research University College London Hospitals Biomedical Research Centre. We acknowledge Exeter Genomics Laboratory for providing the exome testing.

This study was supported by the Italian Ministry of Health (Ricerca Corrente, and Ricerca Finalizzata RF-2016-02361241), the Pierfranco e Luisa Mariani Foundation (CM23). T.B.H. was supported by the Deutsche Forschungsgemeinschaft (DFG, German Research Foundation) – 418081722, 433158657. RM and RWT are supported by the Wellcome Centre for Mitochondrial Research (203105/Z/16/Z), the Medical Research Council (MRC) International Centre for Genomic Medicine in Neuromuscular Disease (MR/S005021/1), the Mitochondrial Disease Patient Cohort (UK) (G0800674), the UK NIHR Biomedical Research Centre for Ageing and Age-related disease award to the Newcastle upon Tyne Foundation Hospitals NHS Trust, the Lily Foundation and the UK NHS Specialist Commissioners which funds the "Rare Mitochondrial Disorders of Adults and Children" Diagnostic Service in Newcastle upon Tyne. RWT also receives financial support from the Pathological Society. AA was supported by a PhD studentship funded by the Kuwait Civil Services Commission. JM are support by the German Federal Ministry of Education and Research (BMBF, Bonn, Germany) and Horizon2020 through the EJP RD project GENOMIT (01GM1920A, genomit.eu). CL, DG, and SBW are funded by the BMBF through ERA PERMED2019-310 – Personalized Mitochondrial Medicine (PerMiM): Optimizing diagnostics and treatment for patients with mitochondrial diseases and by the E-Rare project GENOMIT. HP was supported by the BMBF and Horizon2020 through the EJP RD project GENOMIT (01GM1920A), the ERA PerMed project PerMiM (01KU2016A) and mitoNET (01GM1906B). JAM was supported by the ERA-Net E-Rare project GENOMIT Austrian Science Fund (FWF) I4704-B. CL, AC, and DG are members of the European Reference Network for Rare Neuromuscular Diseases (ERN EURO-NMD). CL and AC were supported by the project GENOMIT (J42F19000030006-RE17) and the Italian Ministry of Health RF-2006-02361495.

Supplemental references

1. Kusikova, K., Feichtinger, R.G., Csillag, B., Kalev, O.K., Weis, S., Duba, H.C., Mayr, J.A.,

Weis. D. (2021). Case Report and Review of the Literature: A New and a Recurrent Variant in the VARS2 Gene Are Associated With Isolated Lethal Hypertrophic Cardiomyopathy, Hyperlactatemia, and Pulmonary Hypertension in Early Infancy. Front Pediatr. *9,* 660076.

2. Rustin, P., Chretien, D., Bourgeron, T., Gérard, B., Rötig, A., Saudubray, J.M., and Munnich, A. (1994). Biochemical and molecular investigations in respiratory chain deficiencies. Clin ChimActa. *228* :35-51
3. Wilfinger, N., Austin, S., Scheiber-Mojdekhar, B., Berger, W., Reipert, S., Praschberger, M., Paur, J.,Trondl, R., Keppler, B. K., Zielinski, C. C., and Nowikovsky, K. (2016). Novel p53-dependent anticancer strategy by targeting iron signaling and BNIP3L-induced mitophagy. Oncotarget. *7*, 1242–1261.
4. Durigon, R., Mitchell, A.L., Jones, A.W., Manole, A., Mennuni, M., Hirst, E.M., Houlden, H., Maragni, G., Lattante, S., Doronzio, P.N., et al. (2018).LETM 1 Couples Mitochondrial DNAMetabolism and Nutrient Preference . EMBO Molecular Medicine. *10*, 1–20.
5. Kuipers, J., and Giepmans, B.N.G. (2020). Neodymium as an alternative contrast for uranium inelectron microscopy, Hitochem. Cell. Biol. 153, 271-277.
6. Yepez, V.A., Mertes, C., Muller, M.F., Klaproth-Andrade, D., Wachutka, L., Fresard, L., Gusic, M.,Scheller, I.F., Goldberg, P.F., Prokisch, H., et al. (2021). Detection of aberrant gene expression events in RNA sequencing data. Nature protocols 16, 1276-1296.
7. Dobin A, Davis CA, Schlesinger F, Drenkow J, Zaleski C, Jha S, et al. STAR: ultrafast universal RNA-seq aligner. Bioinformatics. 2013 Jan;29(1):15–21.
8. Nowikovsky, K., Froschauer, E.M., Zsurka, G., Samaj, J., Reipert, S., Kolisek, M., Wiesenberger, G., Schweyen, R.J. (2004). The LETM1/YOL027 gene family encodes a factor of the mitochondrialK+ homeostasis with a potential role in the Wolf-Hirschhorn syndrome. J Biol Chem. *279*, 30307-30315.
