# Supplementary material for: Bi-allelic LETM1 variants perturb mitochondrial ion homeostasis leading to a clinical spectrum with predominant nervous system involvement
Source: Am J Hum Genet. 2022 Sep 1;109(9):1692–712. doi: 10.1016/j.ajhg.2022.07.007 (PMC9502063; doi:10.1016/j.ajhg.2022.07.007)
Supplement: Document S2. Article plus supplemental information [file mmc7.pdf]

# Bi-allelic *LETM1* variants perturb mitochondrial ion homeostasis leading to a clinical spectrum with predominant nervous system involvement

## Graphical abstract

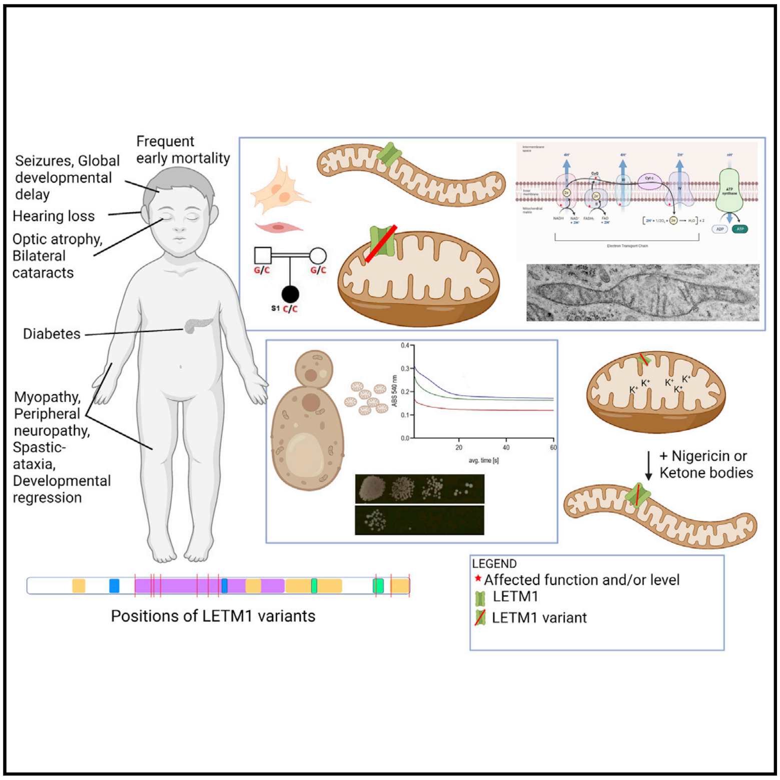


## Authors

Rauan Kaiyrzhanov,
Sami E.M. Mohammed,
Reza Maroofian, ...,
Antonella Spinazzola,
Karin Nowikovsky, Henry Houlden

## Correspondence

karin.nowikovsky@vetmeduni.ac.at
(K.N.),
h.houlden@ucl.ac.uk (H.H.)

**Kaiyrzhanov et al. describe 18 affected individuals with bi-allelic variants in the leucine zipper-EF-hand containing transmembrane protein 1 gene presenting with clinical features suggestive of a mitochondrial disease. Functional studies showed defective mitochondrial $K^+$ efflux, swollen mitochondrial matrix structures, and loss of mitochondrial oxidative phosphorylation protein components.**

Kaiyrzhanov et al., 2022, The American Journal of Human Genetics *109*, 1692–1712
September 1, 2022 © 2022 The Authors.
https://doi.org/10.1016/j.ajhg.2022.07.007

ARTICLE

# Bi-allelic *LETM1* variants perturb mitochondrial ion homeostasis leading to a clinical spectrum with predominant nervous system involvement

Rauan Kaiyrzhanov,[1,40] Sami E.M. Mohammed,[2,40] Reza Maroofian,[1,40] Ralf A. Husain,[3,4] Alessia Catania,[5] Alessandra Torraco,[6] Ahmad Alahmad,[7,8] Marina Dutra-Clarke,[9] Sabine Grønborg,[10] Annapurna Sudarsanam,[11] Julie Vogt,[11] Filippo Arrigoni,[12] Julia Baptista,[13] Shahzad Haider,[14] René G. Feichtinger,[15] Paolo Bernardi,[16] Alessandra Zulian,[16] Mirjana Gusic,[17,18,19] Stephanie Efthymiou,[1] Renkui Bai,[20] Farah Bibi,[21] Alejandro Horga,[1,22] Julian A. Martinez-Agosto,[23] Amanda Lam,[24,25] Andreea Manole,[1] Diego-Perez Rodriguez,[26] Romina Durigon,[26] Angela Pyle,[7] Buthaina Albash,[8] Carlo Dionisi-Vici,[27] David Murphy,[28] Diego Martinelli,[27] Enrico Bugiardini,[1]

*(Author list continued on next page)*

## Summary

Leucine zipper-EF-hand containing transmembrane protein 1 (*LETM1*) encodes an inner mitochondrial membrane protein with an osmoregulatory function controlling mitochondrial volume and ion homeostasis. The putative association of *LETM1* with a human disease was initially suggested in Wolf-Hirschhorn syndrome, a disorder that results from *de novo* monoallelic deletion of chromosome 4p16.3, a region encompassing *LETM1*. Utilizing exome sequencing and international gene-matching efforts, we have identified 18 affected individuals from 11 unrelated families harboring ultra-rare bi-allelic missense and loss-of-function *LETM1* variants and clinical presentations highly suggestive of mitochondrial disease. These manifested as a spectrum of predominantly infantile-onset (14/18, 78%) and variably progressive neurological, metabolic, and dysmorphic symptoms, plus multiple organ dysfunction associated with neurodegeneration. The common features included respiratory chain complex deficiencies (100%), global developmental delay (94%), optic atrophy (83%), sensorineural hearing loss (78%), and cerebellar ataxia (78%) followed by epilepsy (67%), spasticity (53%), and myopathy (50%). Other features included bilateral cataracts (42%), cardiomyopathy (36%), and diabetes (27%). To better understand the pathogenic mechanism of the identified *LETM1* variants, we performed biochemical and morphological studies on mitochondrial $K^+/H^+$ exchange activity, proteins, and shape in proband-derived fibroblasts and muscles and in *Saccharomyces cerevisiae*, which is an important model organism for mitochondrial osmotic regulation. Our results demonstrate that bi-allelic *LETM1* variants are associated with defective mitochondrial $K^+$ efflux, swollen mitochondrial matrix structures, and loss of important mitochondrial oxidative phosphorylation protein components, thus highlighting the implication of perturbed mitochondrial osmoregulation caused by *LETM1* variants in neurological and mitochondrial pathologies.

## Introduction

Leucine zipper-EF-hand containing transmembrane protein 1 (*LETM1*) (MIM: 604407) is a ubiquitously expressed and phylogenetically highly conserved nuclear gene. LETM1, also named SLC55A1, is part of the mitochondrial transporter protein SLC55 family that belongs to the SLC solute carrier superfamily,[1] is the founder of the LETM1 superfamily, and is listed as one of the EF-hand $Ca^{2+}$-binding proteins of the MitoCarta library.[2,3] The proteins of the LETM1 superfamily contain leucine zipper and several coiled-coil domains.[2,4] LETM1 is an inner mitochondrial

[1]Department of Neuromuscular Diseases, University College London, Queen Square, Institute of Neurology, London WC1N 3BG, UK; [2]Department of Biomedical Sciences, Institute of Physiology, Pathophysiology and Biophysics, University of Veterinary Medicine Vienna, Vienna 1210, Austria; [3]Department of Neuropediatrics, Jena University Hospital, Jena 07747, Germany; [4]Center for Rare Diseases, Jena University Hospital, Jena 07747, Germany; [5]Unit of Medical Genetics and Neurogenetics, Fondazione IRCCS Istituto Neurologico Carlo Besta, Milan 20126, Italy; [6]Unit of Muscular and Neurodegenerative Disorders, Laboratory of Molecular Medicine, Bambino Gesù Children's Hospital, IRCCS, Rome 00146, Italy; [7]Wellcome Centre for Mitochondrial Research, Translational and Clinical Research Institute, Faculty of Medical Sciences, Newcastle University, Newcastle Upon Tyne NE2 4HH, UK; [8]Kuwait Medical Genetics Centre, Al-Sabah Medical Area 80901, Kuwait; [9]Division of Medical Genetics, Department of Pediatrics, David Geffen School of Medicine, the University of California at Los Angeles, Los Angeles, CA 90095, USA; [10]Center for Rare Diseases, Department of Pediatrics and Department of Genetics, Copenhagen University Hospital Rigshospitalet, Blegdamsvej 9, Copenhagen 2100, Denmark; [11]West Midlands Regional Genetics Service, Birmingham Women's and Children's Hospital, Birmingham B15 2TG, UK; [12]Paediatric Radiology and Neuroradiology Department, V. Buzzi Children's Hospital, Milan 20154, Italy; [13]Peninsula Medical School, Faculty of Health, University of Plymouth, Plymouth PL4 8AA, UK; [14]Paediatrics Wah Medical College NUMS, Wah Cantonment, Punjab 44000, Pakistan; [15]University Children's Hospital, Salzburger Landeskliniken (SALK) and Paracelsus Medical University (PMU), Salzburg 5020, Austria; [16]Department of Biomedical Sciences, University of Padova, Via Ugo Bassi 58/B, Padova 35131, Italy; [17]Institute of Neurogenomics, Helmholtz Zentrum München, Neuherberg 85764, Germany; [18]DZHK (German Centre for Cardiovascular Research), Partner Site Munich Heart Alliance, Munich 81675, Germany; [19]Institute of Human Genetics, Technical University of Munich, Munich 81675, Germany; [20]GeneDx Inc, Gaithersburg, MD 20877, USA; [21]Institute of Biochemistry and Biotechnology, Pir Mehar Ali Shah Arid Agriculture University, Rawalpindi 44000, Pakistan; [22]Neuromuscular Diseases Unit, Department of Neurology, Hospital Clinico San Carlos and San Carlos Health Research Institute (IdISSC), Madrid 28040, Spain;

*(Affiliations continued on next page)*

© 2022 The Authors. This is an open access article under the CC BY license (http://creativecommons.org/licenses/by/4.0/).

Katrina Allis,[20] Costanza Lamperti,[5] Siegfried Reipert,[29] Lotte Risom,[30] Lucia Laugwitz,[31,32] Michela Di Nottia,[6] Robert McFarland,[7,33] Laura Vilarinho,[34] Michael Hanna,[1] Holger Prokisch,[17,19] Johannes A. Mayr,[15] Enrico Silvio Bertini,[6] Daniele Ghezzi,[5,35] Elsebet Østergaard,[30,36] Saskia B. Wortmann,[15,17,19,37] Rosalba Carrozzo,[6] Tobias B. Haack,[32,38] Robert W. Taylor,[7,33] Antonella Spinazzola,[26,41] Karin Nowikovsky,[2,39,41,*] and Henry Houlden[1,41,*]

membrane protein with an osmoregulatory function that controls cation homeostasis, preventing their equilibration with the $H^+$ electrochemical gradient. While first identified to function as an electroneutral mitochondrial $K^+$-$H^+$ exchanger (KHE), LETM1 has also been connected to the regulation of the uptake or extrusion of $Ca^{2+}$.[2,5–10]

The pathological hallmark of *LETM1* depletion is mitochondrial matrix swelling, fragmentation, and loss of cristae structure, consistently found in all studied organisms,[5] whereas *LETM1* overexpression causes mitochondrial elongation, cristae swelling, and matrix condensation due to imbalance in osmotic homeostasis.[11] Silencing *LETM1* homologs in yeast, *Fusarium graminearum,* and *Toxoplasma gondii* results in lethality or loss of virulence. *Drosophila melanogaster* with tissue-specific depleted *LETM1* displays compromised tissue growth and locomotor behavior, as well as impaired evoked synaptic release of neurotransmitters.[12] The homozygous deletion of *LETM1* leads to developmental and embryonic lethality in flies, worms, and mice.[9,11,12]

Consistent with the vital role of mitochondrial osmoregulation, matrix swelling and cation imbalance due to *LETM1* inactivation have wide-reaching and pleiotropic effects on mitochondrial biogenesis and bioenergetics, perturbing glucose and pyruvate utilization, tryptophan and mtDNA metabolism, and outer mitochondrial membrane integrity and causing necrotic cell death.[9,12–17]

The importance of *LETM1* in neuronal function and pathology was initially suggested in Wolf-Hirschhorn syndrome (WHS [MIM: 194190]).[4] This genetic syndrome results from *de novo* monoallelic deletion of several genes on the short arm of chromosome 4. Depending on the length of the deletion, WHS might present with a combination of congenital malformations, specific facial dysmorphism, growth and cognitive impairment, microcephaly, hypotonia, and epilepsy.[13] *LETM1* is localized in WHS critical region 2 (WHSCR2), less than 80 kb from WHS critical region 1 (WHSCR1), and is deleted in almost all individuals with the full WHS phenotype. *LETM1* is proposed to be associated with epilepsy and neuromuscular features of WHS.[18,19] Analysis of WHS fibroblasts linked *LETM1* haploinsufficiency with mitochondrial defects. One study reports elevated intracellular $Ca^{2+}$, decreased $Ca^{2+}$ sensitivity of the mitochondrial permeability transition pore (PTP), and increased superoxide and hyperpolarization of the inner membrane;[20] another study reports mtDNA aggregation, pyruvate dehydrogenase (PDH) deficiency, and a preferential shift from pyruvate oxidation to ketone body utilization.[14] How the cation transport properties of LETM1 and the broad effects of its dysfunction on other mitochondrial and cellular functions mechanistically contribute to the WHS disease phenotypes is not well understood and is complicated by the multigenic cause for WHS. Other implications of LETM1 impairment in genetic diseases include temporal lobe epilepsy,[21] diabetes,[22] and obesity.[15]

Here, we describe 18 affected individuals from 11 unrelated families presenting with clinical features suggestive of a mitochondrial disease largely involving the CNS in which exome sequencing (ES) identified novel and ultra-rare bi-allelic segregating *LETM1* variants.

To functionally characterize the bi-allelic *LETM1* variants, we explored cellular growth and mitochondrial respiratory chain, morphology, osmotic regulation, and KHE activity in proband-derived fibroblasts, muscle samples, and yeast carrying the variants of interest.

## Subjects and methods

### Study subjects

Using the GeneMatcher platform[23] and data sharing with collaborators around the world, 11 families with bi-allelic *LETM1* variants

[23]Department of Human Genetics, Division of Medical Genetics, Department of Pediatrics, David Geffen School of Medicine, University of California, Los Angeles, Los Angeles, CA 90095, USA; [24]Neurometabolic Unit, National Hospital for Neurology and Neurosurgery, London, UK; [25]Department of Chemical Pathology, Great Ormond Street Hospital, WC1N 3BG London, UK; [26]Department of Clinical Movement Neurosciences, Royal Free Campus, University College of London, Queen Square Institute of Neurology, London WC1N 3BG, UK; [27]Division of Metabolism, Bambino Gesù Children's Hospital, IRCCS, Rome 00146, Italy; [28]Department of Clinical and Movement Neurosciences, UCL Queen Square Institute of Neurology, University College London, London WC1N 3BG, UK; [29]Core Facility of Cell Imaging and Ultrastructure Research, University of Vienna, Djerassiplatz 1, 1030 Wien, Austria; [30]Department of Genetics, Copenhagen University Hospital Rigshospitalet Blegdamsvej, Copenhagen 2100, Denmark; [31]Institute of Medical Genetics and Applied Genomics, University of Tuebingen, 72076 Tübingen, Germany; [32]Department of Neuropediatrics, Developmental Neurology and Social Pediatrics, University of Tübingen, Tübingen 72076, Germany; [33]NHS Highly Specialised Service for Rare Mitochondrial Disorders, Newcastle upon Tyne Hospitals NHS Foundation Trust, Newcastle upon Tyne NE1 4LP, UK; [34]Unit of Neonatal Screening, Metabolism and Genetics, Department of Human Genetics, National Institute of Health Dr Ricardo Jorge, Porto 4000-055, Portugal; [35]Department of Pathophysiology and Transplantation, University of Milan, Milan 20122, Italy; [36]Institute for Clinical Medicine, University of Copenhagen, Copenhagen 2200, Denmark; [37]Radboud Center for Mitochondrial Medicine, Department of Pediatrics, Amalia Children's Hospital, Radboudumc, Nijmegen 6525 EZ, the Netherlands; [38]Centre for Rare Diseases, University of Tuebingen, Tübingen 72076, Germany; [39]Department of Internal Medicine I, ASCTR and Comprehensive Cancer Center, Medical University of Vienna, Vienna 1090, Austria
[40]These authors contributed equally
[41]Senior author
*Correspondence: karin.nowikovsky@vetmeduni.ac.at (K.N.), h.houlden@ucl.ac.uk (H.H.)
https://doi.org/10.1016/j.ajhg.2022.07.007.

**Table 1. Summary of the *LETM1* variants identified in the present cohort and 2 non-pathogenic variants**

| F ID | Center | Method | gDNA Change (chr4 hg 19) | Variant type | nt change | aa change | gnomAD V3.1.2 and V2.1.1 | Other databases | CADD | GERP | SIFT | PolyPhen |
|---|---|---|---|---|---|---|---|---|---|---|---|---|
| 1 | Queen Square Genomics | proband only ES[25–27] | g.1834673A>T | missense, splice region | c.878T>A | p.Ile293Asn | 0 | 0 | 28.8 | 4.61 | D | PD |
| 1 | Queen Square Genomics | proband only ES[25–27] | g.1816277T- | frameshift | c.2094del | p.Asp699Metfs*13 | 0 | 1 het allele (UKBB) | – | – | – | – |
| 2 | Copenhagen University Hospital | proband only ES[28] | g.1816151C>G | stop_loss | c.2220G>C | p.*740Tyrext26 | 0 | 0 | – | – | – | – |
| 7 | Queen Square Genomics | proband only ES[26,27] | | | | | | | | | | |
| 3 | Wellcome Center for Mitochondrial Research | proband only ES[29] | g.1836692CTT- | inframe deletion | c.754_756del | p.Lys252del | 0 | 0 | – | – | – | – |
| 4 | Wellcome Center for Mitochondrial Research | proband only ES[29] | g.1834670C>T | missense | c.881G>A | p.Arg294Gln | 4 het alleles (V2.1.1); 2 het alleles (V3.1.2.) | 2 het alleles (UKBB); 3 het alleles (GeneDx); 2 het alleles (TOPMed) | 26.3 | 4.61 | D | PD |
| 8 | Fondazione IRCCS Istituto Neurolo-gico Carlo Besta, Milan | proband only ES[24] | | | | | | | | | | |
| 5 | Institute of Medical Genetics and Applied Genomics, University of Tuebingen, Germany | proband only ES[30] | g.1834479C>T | missense | c.1072G>A | p.Asp358Asn | 0 | 0 | 23.5 | 4.61 | D | B |
| 6 | GeneDX | trio ES[31–33] | g.1827313-C-T[a] | missense | c.1178G>A | p.Arg393His | 13 het alleles (V2.2.1.1); 2 het alleles (V3.1.2) | AF 0.0002 (1K GP); 1 het allele (UKBB); 7 het alleles (GeneDx); 7 het alleles (TOPMed) | 26.6 | 5.06 | D | PD |
| 9 | Exeter Genomics Laboratory | trio ES[34] | g.1827352C>G | missense | c.1139G>C | p.Arg380Pro | 0 | 1 het allele (UKBB) | 27.4 | 5.06 | D | PD |
| 10 | Institute of Human Genetics, Technical University of Munich | proband only ES[35] | g.1814582G>C | splice defect | c.2071−9C>G | p.Val691fs*4 | 0 | 0 | – | – | – | – |

(*Continued on next page*)

**Table 1. *Continued***

| F ID | Center | Method | gDNA Change (chr4 hg 19) | Variant type | nt change | aa change | gnomAD V3.1.2 and V2.1.1 | Other databases | CADD | GERP | SIFT | PolyPhen |
|---|---|---|---|---|---|---|---|---|---|---|---|---|
| 11 | Bambino Gesù Children's Hospital, IRCCS | proband only ES[36–38] | g.1834653G>A | missense | c.898C>T | p.Pro300Ser | 0 | 2 het alleles (GeneDx) | 25.8 | 4.61 | D | PD |
| Non-pathogenic variant 1 | | | g.1834638T>G | missense | c.913A>C | p.Ile305Leu | 1 het allele (V2.1.1); 3 het alleles, 1 hom allele (V3.1.2) | 4 het alleles (TOPMed) | 27.6 | 4.61 | D | PD |
| Non-pathogenic variant 2 | | | g.1818625T>A | missense | c.1760A>G | p.Lys587Arg | 2,756 het alleles, 39 hom alleles (V2.1.1); 2,354 het alleles, 34 hom alleles (V3.1.2) | 43,024.9 het alleles, 1 hom allele; AF 0.002, 4 hom carriers (UKBB); 4,367 het alleles and 82 hom alleles (TOPMed) | 25.3 | 5.04 | D | PD |

*LETM1* isoform is GenBank: NM_012318.3. F, family; ES, exome sequencing; gDNA, genomic DNA; nt, nucleotide; aa, amino acid; D, deleterious; PD, probably damaging; AF, allele frequency; het, heterozygous; hom, homozygous. Other databases: Queen Square Genomics database (23K exomes), ESP, Iranome, 1K GP (1000 Genomes global minor allele frequency), UKBB (UK Biobank), GeneDx database, Middle Eastern database, TOPMed.
[a]A homozygous *LETM1* variant due to maternal uniparental disomy.

were identified. The affected individual from family 8 was recruited from the report by Catania et al.[24] describing a person with a combined pituitary hormone deficiency, ocular involvement, myopathy, ataxia, and mitochondrial impairment carrying variants in several putative disease-causing genes, including rare bi-allelic variants in *OTX2* (orthodenticle homeobox 2 [MIM: 600037]) and *LETM1* as well as rare heterozygous variants in *AFG3L2* (AFG3 like matrix AAA peptidase subunit 2 [MIM: 604581]) and *POLG* (DNA polymerase gamma, catalytic subunit [MIM: 174763]). Clinical details of the cohort were obtained by the follow-up of the living affected individuals and retrospective analysis of the available clinical records for deceased individuals. Parents and legal guardians of all affected individuals gave their consent for the publication of clinical and genetic information according to the Declaration of Helsinki, and the study was approved by The Research Ethics Committee Institute of Neurology University College London (IoN UCL) (07/Q0512/26) and the local Ethics Committees of each participating center. Consent has been obtained from families 1, 5, and 8 to publish medical photographs and video examinations. Brain magnetic resonance imaging (MRI) scans were obtained from 6 affected individuals and were reviewed by an experienced pediatric neuroradiologist (FA).

## Exome sequencing and data analysis

Proband only or trio ES in 11 families was carried out in DNA extracted from blood-derived leukocytes in 9 different centers following slightly different protocols (see Table 1). ES data analysis and variant filtering and prioritization were performed using in-house implemented pipelines of the local genetic centers (Table 1). Sanger sequencing was performed to confirm co-segregation in all available family members.

## Skin biopsy and primary fibroblast culture and muscle biopsy

Individuals F1:S1, F1:S2 and parents (mother-F1:M, father-F1:F), F2:S1, F5:S1, F10:S1, F11:S1, and F11:S2 provided each one skin biopsy, and affected individuals F11:S1, F11:S2, and F5:S1 provided also each one muscle biopsy. Details on fibroblast cell lines establishment and muscle sample preparations are described in the supplemental material and methods.

## Western blotting analysis

Immunoblotting analysis was performed using standard protocols as described previously;[39] detailed descriptions of sample preparation, quantification, and western blotting are in the supplemental material and methods. A list of antibodies used for this study is given in supplemental data.

## Cell imaging

Confocal microscopy was performed for fibroblasts from F1, F2, F5, F10, and F11 and respective control subjects following established protocols for life and immune staining described in Durigon et al.,[14] Wilfinger et al.,[40] and supplemental material and methods. Transmission electron microscopy is described in the supplemental material and methods.

## mtDNA copy number

DNA was extracted from muscle or fibroblasts by proteinase K treatment. The mtDNA content was determined by quantitative

real-time PCR using two independent mitochondrial and four independent nuclear DNA sequences as previously described.[41]

### Immunohistochemistry

FFPE muscle tissue was cut with a microtome in 4 μm slides. Immunohistochemistry was performed as described previously in Kusikova et al.[39] with some modifications (a detailed description of the method is given in the supplemental material and methods). All antibodies used in this experiment are listed in supplemental material and methods.

### Plasmid and *LETM1* single-nucleotide variants

Full-length human *LETM1* cDNA fused to C-terminal Hemagglutinin (*HA*)-tag and subcloned into the multi-copy plasmid pVT-103U[42] served as a template to introduce the *LETM1* variants by site-directed mutagenesis. Amino acid replacements and deletions were performed with non-overlapping back-to-back annealing mutagenic primers, using the Q5 site-directed mutagenesis kit (NEB #E0552S) with NEB 5-alpha competent *E. coli* cells (NEB #C2987). All primers were from Microsynth and all the identified variants were confirmed by DNA sanger sequencing. To distinguish the phenotypes of disease-associated *LETM1* variants and non-pathogenic variants, two non-disease-associated *LETM1* (GenBank: NM_012,318.3) missense variants (rare *LETM1* variants but with homozygotes in gnomAD v3.1.1), c.913A>C (p.Ile305Leu) and c.1760A>G (p.Lys587Arg), were included in this study. A list of variants studied in yeast and primers used for site-directed mutagenesis is given in supplemental data.

### Yeast transformation

W303 (ATCC 201239) *Saccharomyces cerevisiae* strain *mdm38/letm1Δ* (lacking the open reading frame *YOL027c*, which encodes the yeast LETM1 homolog)[42] was transformed with the multi-copy vector pVT-103U, either empty or containing wild-type human *LETM1*[42] or *LETM1* variants using the lithium acetate/single-stranded carrier DNA/polyethylene glycol method[43] and grown on selective media (SD-URA) to ensure the retention of the plasmids. Yeast growth media were described in Zotova et al.[44]

### Mitochondrial isolation and KOAc-induced swelling assay

Mitochondria were isolated from yeast cells logarithmically grown in SD-URA by homogenization and differential centrifugation method as described in Nowikovsky et al.[42] and immediately used for KOAc-induced swelling assays. The protocols of Nowikovsky et al.[42] were adapted to smaller volumes. In brief, isolated yeast mitochondria suspended in breaking buffer (0.6 M sorbitol, 20 mM Tris-HCl [pH 7.4]) were de-energized with antimycin A (2.5 μM) for 10 min at room temperature (25°C), washed, and resuspended in breaking buffer at a concentration of 200 μg/20 μL. As $Mg^{2+}$ is a brake to the KHE,[45] mitochondria were depleted from $Mg^{2+}$ with A23187 (0.5 μM) and EDTA (10 mM) and transferred onto 96-well plates for measurement (200 μg/well). When indicated, quinine (200 μM) served as a control to inhibit KHE-mediated swelling. The 96-well plates were placed in the Thermo Scientific Varioskan LUX Multimode Microplate Reader. The swelling was initiated by injection of KOAc media (55 mM KOAc, 5 mM TES, 0.1 mM EDTA) to a final volume of 200 μL/well and the optical density changes at $OD_{540}$ were immediately recorded at 25°C. Each measurement was performed in 3 independent replicates. Raw swelling data were fitted into a curve showing changes in absorbance versus time to quantify the swelling rate.

## Results

### Clinical findings

The summary of the core phenotypic features of 18 affected individuals from 11 independent families with bi-allelic *LETM1* variants is provided in Table 2, Figure 1C, and Table S1. Detailed clinical history is provided in the supplemental note (case reports). Video recordings are available for affected individuals from family 1 (Videos S1, S2, S3, and S4). The cohort comprises 10 males and 8 females, 9 of whom are currently alive with a median age of 15 years (range 1–39) at the latest available follow-up (Figure 2A). Half of the persons (9/18) succumbed to their rapidly progressing disease at an early age, ranging between 2 months and 8 years old. The ethnic composition of the cohort is diverse, including families of Pakistani, Caucasus, Middle Eastern, European, and Mexican origin, with 67% of the individuals (12/18) being from consanguineous unions. Only limited clinical data were obtainable from 6 deceased persons belonging to families 3 and 10.

The cohort members had unremarkable prenatal histories with full-term birth in 14/15 persons (93%). Admission to the special care baby unit was necessary in 5/15 affected individuals (33%) due to respiratory, cardiac, and feeding issues during the neonatal period. Most of the persons (14/18, 78%) had an infantile-onset disease manifestation, and 4/18 (22%) presented first symptoms between the ages of 1.5 and 2 years. The common presenting symptoms were global developmental delay, cognitive and motor regression, failure to thrive, central hypotonia, respiratory distress, and feeding difficulties. The disease progressed rapidly in 9/18 (50%), moderately fast in 4/18 (22%), and slowly in 5/18 (28%) affected individuals. Developmental regression was later present in 9/13 (69%) affected individuals with loss of independent ambulation by a mean age of 5.4 ± 3.2 years (range 2–12).

On the most recent follow-up, the affected individuals displayed clinical features suggestive of a mitochondrial disorder. Impaired vision (10/10, 100%) with a mean onset age of 5.2 ± 3.1 years, which was confirmed to be due to optic atrophy in 5/6 (83%), and bilateral sensorineural hearing loss (11/14, 78%) diagnosed at a mean age of 2.6 ± 1.9 years (range from congenital up to 6 years) with hearing aids fitted in 7/10 (70%) persons were the common neurosensory abnormalities. While cognitive delay and intellectual disability (7/8, 87.5%%) and impaired speech acquisition (6/9, 67%) were among the common neurodevelopmental symptoms, more than half of the individuals displayed neuromuscular features including spasticity (8/15, 53%), hypotonia (11/18, 61%), muscular wasting (7/10, 70%), and cerebellar ataxia (7/9, 78%). Other frequent neurological symptoms were nystagmus

**Table 2. Clinical features of affected individuals with bi-allelic *LETM1* variants**

| Family ID | F1 | | F2 | F3 | | | F4 | F5 | F6 | F7 | | F8 | F9 | F10 | | | F11 | |
|---|---|---|---|---|---|---|---|---|---|---|---|---|---|---|---|---|---|---|
| Subject ID | S1 | S2 | S1 | S1 | S2 | S3 | S1 | S1 | S1 | S1 | S2 | S1 | S1 | S1 | S2 | S3 | S1 | S2 |
| **Epidemiology and medical history** | | | | | | | | | | | | | | | | | | |
| Sex | F | M | M | F | M | M | M | M | F | M | M | F | F | F | M | F | F | M |
| Current age/death age | 35 y | 25 y | 24 y | D 1 y | D 2.7 y | D 1 y | D 8 y | 11 y | 17 m | 15 y | 8 y | 39 y | 1 y | D 10 m | D 2 m | D 2 m | D 6 y | D 4.5 m |
| Age at onset | 1 y | 1.5 y | 2.5 y | 4 m | 6 m | 4 m | 4 m | 7 m | birth | 1.5 y | 2 y | 10 m | birth | 4 m | 1 m | birth | birth | birth |
| Type of progression | S | S | S | R | R | R | MD | MD | S | MD | MD | S | R | R | R | R | R | R |
| GDD/ID | + | + | + | + | + | + | + | – | + | + | + | + | + | + | + | + | + | + |
| Regression in development | + | + | + | N/D | – | N/D | – | – | – | + | + | + | + | N/D | N/D | N/D | + | + |
| Loss of ambulation (age) | +, 12 y | +, 6 y | N/D | N/D | N/D | N/D | +, 2.5 y | – | N/D | +, 5 y | +, 5 y | +, 2 y | N/D | N/D | N/D | N/D | N/D | N/D |
| **Main clinical features** | | | | | | | | | | | | | | | | | | |
| Age at last examination | 35 y | 25 y | 24 y | >1 y | >1 y | >1 y | N/D | 11 y | 2 m | 15 y | 8 y | 37 | 1 | N/D | N/D | N/D | 5 y | N/D |
| Small weight and height | + | + | + | N/D | N/D | N/D | N/D | + | – | + | + | + | + | N/D | N/D | N/D | – | – |
| Facial dysmorphism | + | + | – | N/D | N/D | N/D | – | – | + | – | – | + | – | N/D | N/D | N/D | N/D | N/D |
| Optic atrophy/ impaired vision | + | + | + | N/D | + | N/D | + | + | N/D | + | + | + | N/D | N/D | N/D | N/D | + | N/D |
| Cataract | – | – | + | N/D | N/D | N/D | + | – | N/D | – | – | + | – | N/D | N/D | N/D | + | + |
| Sensorineural deafness | + | + | + | N/D | + | N/D | + | + | – | – | – | + | + | + | N/D | N/D | + | + |
| Hypotonia | – | – | – | + | + | + | + | + | – | – | – | + | + | – | + | + | + | + |
| Spasticity/hypertonia | + | + | + | N/D | N/D | N/D | – | – | – | + | + | – | – | + | + | + | – | – |
| Cerebellar ataxia | + | + | N/D | N/D | N/D | N/D | – | – | N/A | + | + | + | N/A | + | N/D | N/D | + | N/D |
| Myopathy | – | – | – | N/D | + | N/D | + | + | N/D | – | – | + | – | N/D | N/D | N/D | + | + |
| Hyperkinetic movement disorders | + | + | + | N/D | N/D | N/D | N/D | – | – | – | – | – | – | + | N/D | N/D | – | – |
| Peripheral neuropathy | + | + | N/D | N/D | N/D | N/D | – | – | N/D | – | – | – | – | N/D | N/D | N/D | + | N/D |
| Impaired speech/ language abilities | + | + | + | N/D | N/D | N/D | – | – | – | + | + | + | N/A | N/D | N/D | N/D | N/D | N/D |
| Impaired/spastic/ataxic gait | + | + | + | N/A | N/A | – | – | – | N/A | + | + | + | N/A | N/D | N/D | N/D | N/D | N/D |
| Seizures | + | + | + | N/D | N/D | N/D | – | – | – | + | + | – | + | + | + | + | + | – |
| Cardiac involvement | – | – | – | N/D | N/D | + | + | – | – | – | – | – | – | N/D | + | N/D | + | + |
| Diabetes | + | + | – | N/D | N/D | N/D | N/D | + | – | – | – | – | – | N/D | N/D | N/D | – | – |
| Lactic acidosis | – | – | – | N/D | + | N/D | + | + | N/D | N/D | N/D | N/D | – | + | + | + | + | + |
| Raised urinary 3-MGA | – | – | + | N/D | – | N/D | – | – | – | N/D | N/D | + | – | + | + | + | N/D | N/D |
| **Investigations** | | | | | | | | | | | | | | | | | | |
| MRC deficiencies | CI, II, III, IV | CI, IV | CII | N/D | CI, II, III, IV | N/D | N/D | CI, III, IV | N/D | N/D | N/D | CI, III, IV, V | CIV | CI, IV | N/D | CI | CI, IV | CI, IV |
| Muscle histochemistry | + | + | N/D | N/D | + | N/D | N/D | + | N/D | N/D | N/D | + | + | N/D | N/D | N/D | + | – |
| Brain MRI findings | CA, PA | N/D | UR | VM | UR | N/D | BA | ONA, CHA | CVH | N/D | ONA, CHA | BA, CA | UR | N/D | N/D | N/D | CVH, BSH, VM, DM | N/D |

Abbreviations: F, female; M, male; y, year; m, months; D, deceased; +, yes; −, no; N/D, no data; S, slow; MD, moderate; R, rapid; N/A, not applicable; GDD, global developmental delay; ID, intellectual disability; MCR, mitochondrial respiratory complex; C, complex; UR, unremarkable; 3-MGA, 3-methylglutaconic aciduria; CA, cerebellar atrophy; PA, pontine atrophy; VM, ventriculomegaly; BA, brain atrophy, ONA, optic nerve atrophy; CHA, chiasmal atrophy; CVH, cerebellar vermis hypoplasia; BSH, brain stem hypoplasia; DM, delayed myelination.

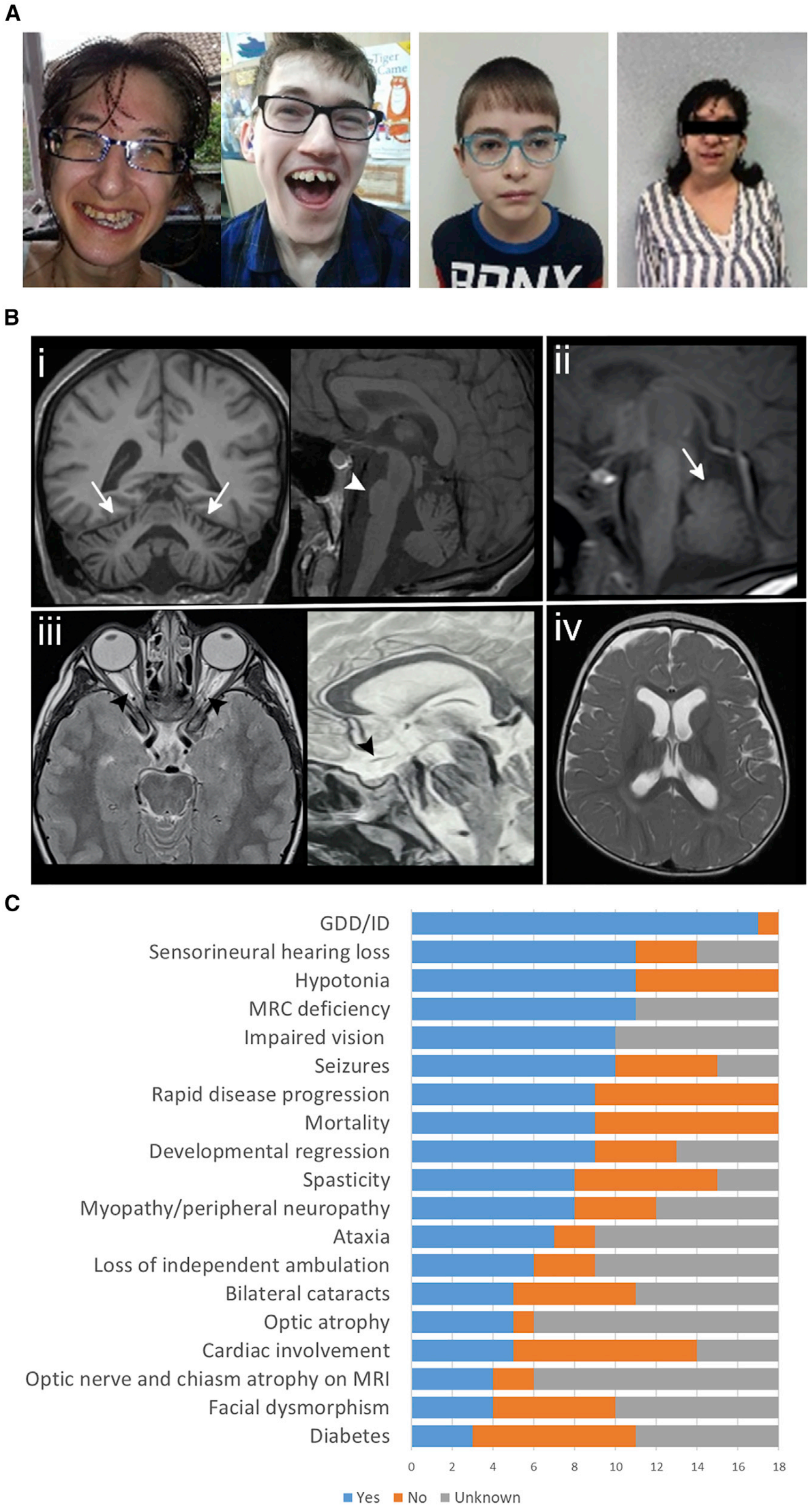


**Figure 1. Clinical features and neuroimaging findings of the individuals with bi-allelic *LETM1* variants**

(A) From left to right, facial photos of the affected individuals F1:S1, F1:S2, F5:S1, and F8:S1. All persons wear glasses due to bilateral optic atrophy. All persons have prominent noses. F1:S1 and F1:S2 show long thin faces, low-set ears, and teeth abnormalities.

(B) In (i) (F1:S1), severe cerebellar atrophy (arrows) and pontine hypoplasia (arrowheads) are shown, while in (ii) (F6:S1), only mild vermian hypoplasia is noted. In (iii) arrowheads point at the severe optic nerve and chiasm atrophy in 2 different individuals (F5:S1 and F7:S2). Mild ventricular dilatation is present in (iv) (F3:S1).

(C) Clinical features of the affected individuals with bi-allelic *LETM1* variants. GDD, global developmental delay; ID, intellectual disability; MRI, magnetic resonance imaging; MRC, mitochondrial respiratory chain.

(7/13, 54%), myopathy (6/12, 50%), hyperkinetic movement disorders (4/12, 33%), and spastic-ataxic gait (3/9, 33%) combined with brisk deep tendon reflexes (4/10, 40%), upgoing plantar response (4/9, 44%), and peripheral neuropathy (3/9, 33%).

Ten of the fifteen affected individuals (67%) developed epileptic seizures by a median age of 5 years (range 0.5–14). The seizure type ranged from infantile spasms and myoclonic jerks to absences, focal, and generalized tonic-clonic seizures. Individuals with younger age of seizure onset had frequent episodes spanning from hourly clusters of spasms at peak to seizures once per day. Two affected siblings from family 1 with seizure onset after ages 9 and 14 years, respectively, had seizures recurring either in clusters 2–3 times every 2–3 months (F1:S2) or once in 2 years (F1:S1). Pharmacoresistance and epileptic encephalopathy were confirmed in one person from family 9. Electroencephalograms, available from 4 individuals, showed background slowing (F5:S1), excessive sharp transients (F6:S1), single 3–4 Hz potentials and short trains (F2:S1), and continuous spike-and-slow wave activity, with bursts of faster activity observed during sleep, consistent with epileptic encephalopathy (F9:S1).

Other features consistent with a mitochondrial phenotype included bilateral cataracts (5/11, 45%) cardiomyopathy (5/14, 36%) with pericardial effusion (3/11, 27%), and diabetes (3/11, 27%). Craniofacial abnormalities included occipitofrontal circumference below third percentile in 2/6 persons (33%) and facial dysmorphism (4/10, 40%) with

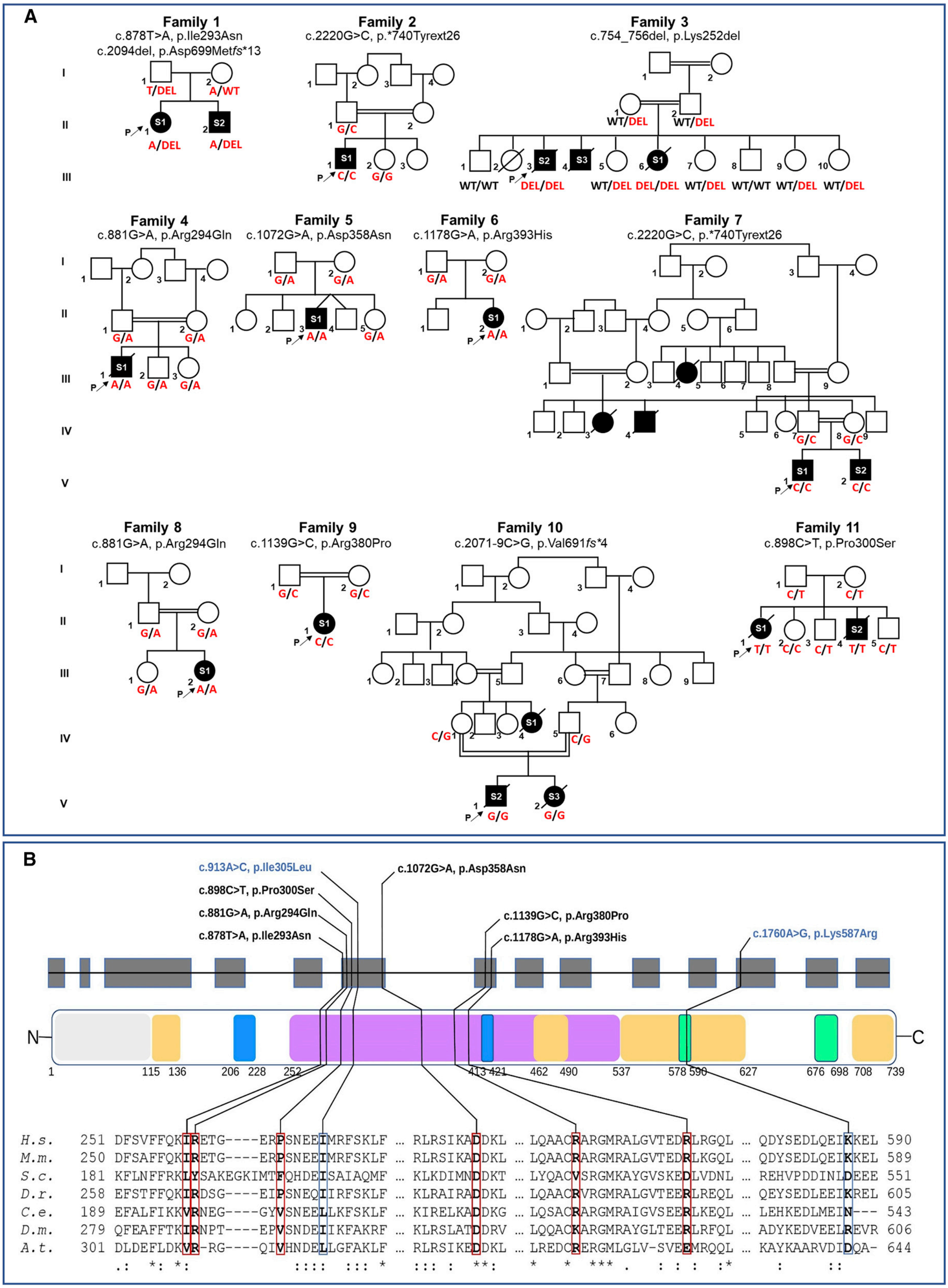


**Figure 2. Pedigrees with the segregations of the *LETM1* variants and LETM1 protein architecture with a partial sequence alignment of the variants**

(A) Family trees of the individuals with bi-allelic *LETM1* variants. Square, male; circle, female; black symbols, affected individuals; white symbols, unaffected individuals.

(B) Schematic representation of the human *LETM1* organization in introns, shown as a line, and exons, shown as boxes, and of LETM1 domains as indicated by the residue numbers and the color code: coiled-coil motifs, light yellow; transmembrane helices, blue;

*(legend continued on next page)*

a long thin face, prominent nose, low-set ears, micrognathia, high arched palate, and teeth abnormalities (Figure 1A).

While not every person had available electrophysiological investigations, biochemical, metabolic studies, and muscle histochemical analysis, the obtainable tests suggested the presence of mitochondrial dysfunction in the affected individuals. Hence, electromyography and nerve conductions studies available from 5 individuals showed neurogenic (3/5) and myopathic changes (2/4). Elevated serum lactate was confirmed in 8/12 (67%) affected individuals. Plasma amino acids were abnormal in 4/9 tested with mildly elevated alanine (501–597 μmol/L, normal range 232–494), glycine, and serine. CSF-alanine was tested and mildly increased in 2 probands. Urine amino acids were tested in 4 persons, and only one person showed abnormal results including increased levels of aspartic, serine, and glycine. Urine organic acids were analyzed in 11 affected individuals and were abnormal in 9 of them with 3-methylglutaconic acid excretion (5/11), moderately elevated beta-hydroxybutyrate and acetoacetate (1/11), and significant elevation of adipic acid (1/11). Muscle biopsy was available from 7 persons and of these, 5 had abnormal findings including scattered necrotic and regenerating COX-deficient fibers with an excess of internal nuclei, lipid depositions within fibers and prominent mitochondrial pattern in vacuolated fibers (F3:S2), COX-deficient multiple ragged-red fibers with increased fiber unisometry (F8:S1), type I fiber predominance with mild glycogen storage (F9:S1), and COX-deficient fibers (F11:S1). Respiratory chain enzyme (RCE) analysis was performed in 11 individuals showing isolated or combined mitochondrial respiratory chain deficiencies in all persons tested (Tables 2, S1, Figure 3C).

Brain MRI investigations were available for 6 persons, performed between 6 days and 32 years of age (Figure 1B). In some persons, only a few sections or low-quality images could be reviewed. In 4/6 affected individuals, optic nerve and chiasm atrophy were present and in two persons optic nerves were normal. Three individuals showed infratentorial abnormalities, with severe pontine hypoplasia and cerebellar atrophy in a proband from family 1 and mild vermian hypoplasia in 2 probands from family 3 and family 6. Other minor and non-specific findings were mild supratentorial atrophy and mild ventricular dilatation noted in 2 persons each.

The affected individual from family 8 was the oldest member of the cohort showing a phenotype consistent with the rest of the individuals that survived into adulthood.

### Molecular genetic findings

In all probands, ES at the local genetic centers did not identify causative variants in known disease-associated genes. Filtering for novel (i.e., not present in available databases) and rare protein-altering variants identified bi-allelic variants in *LETM1* (GenBank: NM_012,318.3) in probands from all families (Table 1). Segregation by Sanger sequencing in families with proband-only ES and where available trio ES supported *LETM1* as a candidate gene (Figure 2A). The proband from family 6 carried a homozygous c.1178G>A (p.Arg393His) variant in *LETM1* resulting from maternal uniparental disomy. Known pathogenic variants in mtDNA and mtDNA rearrangements were excluded in all families.

The *LETM1* variants (Table 1 for variant characterization and Figure 2B) comprised missense variants causing changes in amino acid charge, size, hydrophobic or “helix breaker” properties, and frameshift variants causing premature or delayed termination. All detected missense variants were located specifically within the conserved LETM domain, while the frameshift variants were localized to the C-terminal part of LETM1 (Figure 2B). Of all the amino acid changes, the only fully conserved amino acid across mammals, vertebrates, invertebrates, plants, and yeast is Asp358, and the semi-conserved ones are Lys252 and Ile293 (Figure 2B). Arg294, which is affected by the missense variant c.881G>A (p.Arg294Gln), is conserved in all sub-families excluding yeast, and it was found in two independent persons (F4:S1 and F8:S1) of Egyptian and Italian origin, respectively. Pro300, affected by the variant c.898C>T (p.Pro300Ser), is conserved in mammals and zebrafish. Four variants affect the C-terminal stretch of human LETM1 that is absent in the yeast LETM1 homolog (Letm1p/Mdm38p) as its protein sequence is shorter. The splice variant c.2071−9C>G (p.Val691fs4*) (Sashimi plot, Figure S2, supplemental material and methods) affects two residues conserved across mammals, zebrafish, worms, and plants and introduces a premature stop codon before the second EF loop. The variant c.2094del removes Asp699, a negatively charged residue, well-conserved in mammals, fish, worms, and plants that locates close to the second EF loop and prematurely terminates the protein sequence. The stop-loss variant c.2220G>C (p.*740Tyrext26) leads to an elongation of 26 amino acids. This variant was present in two independent families of Pakistani origin suggesting a possible founder effect. Five of the ten identified *LETM1* variants were absent across a number of large genetic databases (~1 million alleles), whereas the remaining four variants appear to be ultra-rare (Table 1).

LETM/ribosomal-binding like domain, lavender; and putative EF-hands, green. All identified missense variants in the affected individuals (black) and non-pathogenic variants (blue) are mapped according to their positions. The amino acid sequence of human LETM1 was aligned with LETM1 orthologs using Clustal Omega and alignments with LETM1 from other species are shown for all segments that contain missense variants, indicated in bold red letter. Residue conservation is shown below the alignment as fully conserved (*), highly conserved (:), or partially conserved (.). UniProt accession numbers for *H.s.* (*H. sapiens*), *M.m.* (*M. musculus*), *S.c.* (*S. cerevisiae*), *D.r.* (*D. rerio*), *C.e.* (*C. elegans*), *D.m.* (*D. melanogaster*), and *A.t.* (*A. thaliana*) LETM1 used in this alignment are O95202, Q9Z2I0, Q08179, Q1LY46, Q9XVM0, P91927, and F4J9G6, respectively.

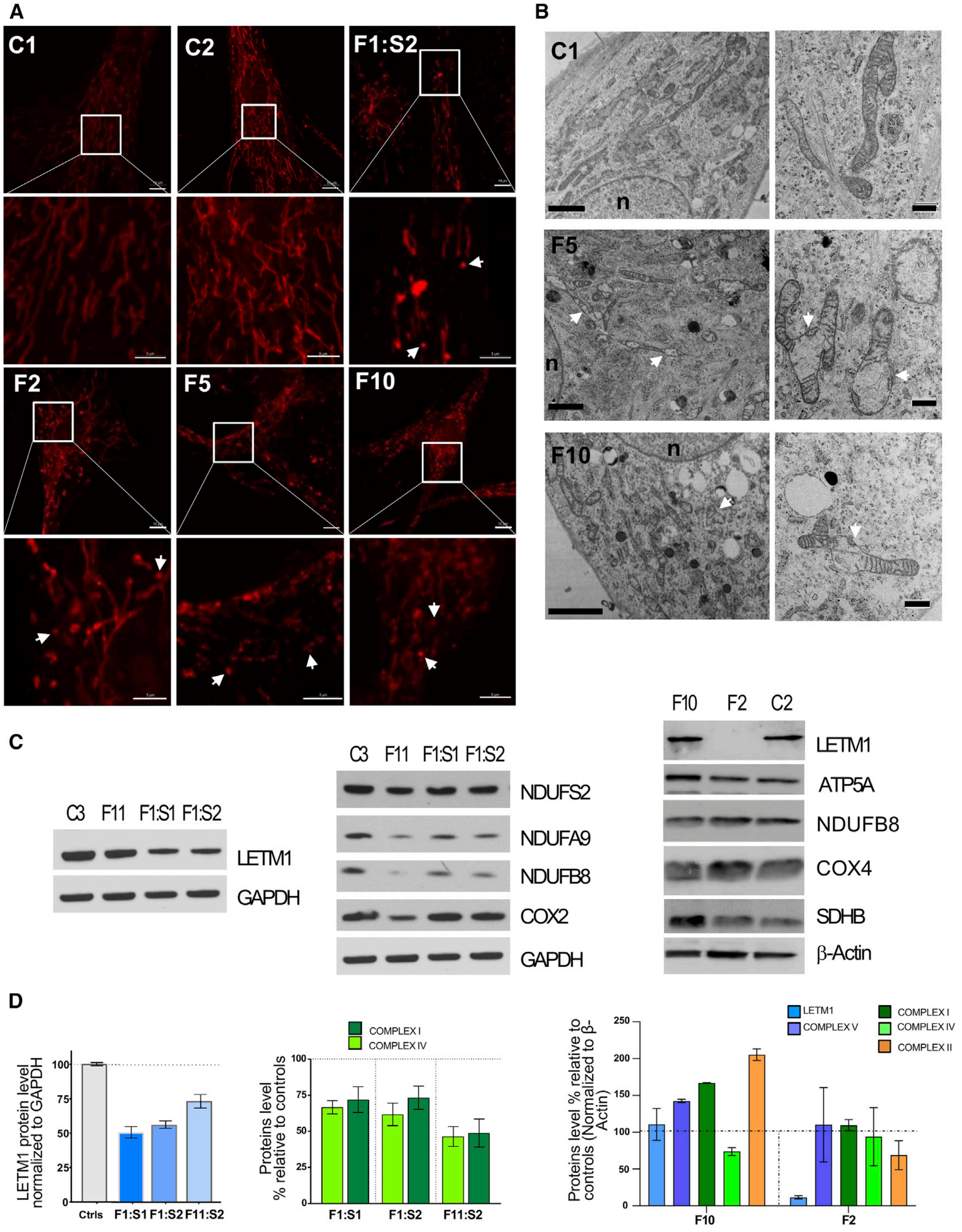


**Figure 3. Effects of *LETM1* variants on mitochondrial morphology and proliferation in fibroblasts**

(A) *LETM1* variants perturb the mitochondrial network. Confocal images of fibroblasts stained with Mitotracker Red. Shown is a representative overview of the cells (bars 5 μm, except F10 10 μm) and details magnified from the box (bars 5 μm). C1 and C2, healthy donors; F1:S2, c.878T>A (p.Ile293Asn) and c.2094del (p.Asp699Metfs*13); F2, c.2220G>C (p.*740Tyrext26); F5, c.1072G>A (p.Asp358Asn); F10, c.2071−9C>G (p.Val691fs4*). Arrow indicates representative fragmented mitochondria. For statistics, see Figure S1C.

(B) *LETM1* variants cause swollen mitochondria and loss of cristae. The ultrastructure of control (C1) and affected individual (F5 and F10) fibroblasts was investigated by transmission electron microscopy and images show overviews (left panels, bars 2 μm) and details (right panels, bar 500 nm). Arrow indicates swollen mitochondria.

(C and D) Variants differently affect LETM1 stability and OXPHOS proteins in fibroblasts samples. Total lysates of fibroblasts were analyzed by immunoblotting using the indicated antibodies, GAPDH, or β-actin as loading control: C2 and C3, healthy donors; F1:S1 and F1:S2, c.[878T>A; 2094del], p.[Ile293Asn; Asp699Metfs*13]; F2, c.2220G>C (p.*740Tyrext26); F10, c.2071−9C>G (p.Val691fs4*); F11, c.898C>T (p.Pro300Ser) (C). Quantitative graphs from independent experiments representing the protein bands, normalized to the housekeeping proteins, and calculated as a percentage of controls; data are expressed as mean ± SEM (n ≥ 3 independent experiments) (D).

### Genotype-phenotype correlation

A remarkable interfamilial phenotypic variability was observed in the present cohort. Four persons from families 1, 2, and 8 have survived into adulthood albeit with a significant disability, while 10 individuals from families 3, 4, 9, 10, and 11 had a rapidly progressing disease course leading to early death in 9 of them. Affected individuals from family 5 (age 11 years), family 6 (age 17 months), and family 7 (ages 8 and 15 years) displayed less severe phenotypes. Affected individuals from family 4 and family 8 carrying the recurrent missense *LETM1* c.881G>A (p.Arg294Gln) variant exhibited a similar range of symptoms, though F4:S1 displayed more rapid disease progression with significant cardiac involvement and early mortality. Persons of Pakistani origin from family 2 and family 7 with loss-of-function (LoF) *LETM1* c.2220G>C (p.*740Tyrext26) variant were reported with a similar phenotypic range, which was more severe in family 2, possibly due to older age and longer disease course. No significant intrafamilial phenotypic variability was observed in the cohort.

### Effects of the *LETM1* variants on patient-derived fibroblasts and muscle tissue

Loss of mitochondrial volume homeostasis is the most characteristic and universally accepted phenotype of *LETM1* deficiency in human, animal models, plants, and yeast, which leads to mitochondrial fragmentation, matrix swelling, and disorganized cristae as reviewed in Austin et al.[5] Therefore, we first evaluated the mitochondrial morphology in the available fibroblasts. Compared to fibroblasts from healthy donors (C1–C4), fibroblasts from F1:S1 and F1:S2 (compound heterozygous for c.[878T>A; 2094del], p.[Ile293Asn; Asp699Metf*13]), F10 (homozygous for c.2071−9C>G [p.Val691fs4*]), F2 (homozygous for c.2220G>C [p.*740Tyrext26]), F5 (homozygous for c.1072G>A [p.Asp358Asn]), and F11:S2 (homozygous for c.898C>T [p.Pro300Ser]) displayed mitochondrial alterations, with significantly increased fragmented shapes seen as donut segments and punctate and enlarged units often separated from the main network (Figures 3A and S1A–S1C). Elongated mitochondrial shapes were restored by ketone bodies and by nigericin, as both reverted the ratio of elongated tubules versus fragmented units to control levels (Figure S1C). The use of the membrane potential-dependent mitochondrial dye Mitotracker Red (MTR) revealed an irregular polarization pattern of the mitochondrial network of all affected individuals, with partly depolarized tubules and hyperpolarized patches, as well as a markedly reduced electric potential of mitochondria in F10 and trend-wise also in F5 (Figures 3A, S1A, and S1C). Impaired KHE activity in LETM1-deficient cells leads to uncompensated electrophoretic $K^+$ uptake and consequent mitochondrial swelling.[13] Treatment with the synthetic KHE nigericin to counteract the loss of $K^+$ homeostasis reverted the decreased membrane potential to control levels in F10 and F5 (Figures S1A and S1C), while addition of ketone bodies had no beneficial effect. Consistent with the electrophoretic $K^+$ influx rate exceeding the $K^+$ release rate due to a lack of KHE activity, mitochondria in F11:S2 cells readily underwent swelling and depolarization (as assessed by *in situ* staining with the potentiometric probe TMRM) upon the addition of low concentrations of valinomycin, a selective $K^+$ ionophore that allows electrophoretic $K^+$ uptake unlike mitochondria of control fibroblasts. Treatment of F11:S2 fibroblasts with the ionophore nigericin restored the mitochondrial sensitivity to valinomycin, a strong indication that the response to valinomycin was indeed due to lack of KHE activity (Figure S1D). Based on the protective effect of ketone bodies as an energy source for LETM1-deficient cells,[14] we tested next whether a tubular network could be better maintained as a result. Ketone bodies suppressed MTR fluorescence in fibroblasts from F1 and F2 and attenuated its intensity in F10:S1. However, increasing the laser intensity, an elongated tubular shape of the mitochondrial network also became apparent in the samples of F1:M and F5:S1 (Figure S1A). Thus, elongation of mitochondrial tubules was accompanied by a reduced inner membrane potential, a phenomenon previously described in the context of transient matrix contraction.[46] Replacement of glucose with galactose, known to suppress glycolytic ATP production, in F1:S1 and F11:S2 for up to 5 days produced a more dramatic morphological phenotype, in some persons resembling LETM1 siRNA (Figures S1B) and Durigon et al.[14] and it caused cell death after only 48–72 h in F11:S2. Transmission electron microscopy was performed for F5 and F10 fibroblasts as well as control fibroblasts and confirmed ultrastructural mitochondrial changes associated with *LETM1* variants compared to the elongated tubular shapes of the healthy control mitochondria (Figure 3B). Different morphological stages of mitochondrial alterations were associated with *LETM1* c.2071−9C>G (p.Val691fs4*) (F10), including short tubules containing enlarged sections with reduced cristae, swollen matrix devoid of cristae, and perinuclearly distributed spherical ghost shapes resembling a mixture of mitochondrial remnants and vacuoles. Similarly, fibroblasts with the variant *LETM1* c.1072G>A (p.Asp358Asn) (F5) showed broad, short, and electron-luce mitochondria, partly devoid of cristae and intermediate shapes between mitochondria and vacuoles.

Pathological variants frequently lead to altered expression or stability of the encoded proteins, and so we assessed LETM1 protein levels via immunoblotting. The steady-state levels of LETM1 in fibroblasts from F10 were comparable to those from control subjects. Instead, LETM1 was significantly decreased in bi-allelic *LETM1* variant fibroblasts F1:S1 and F1:S2, and F11:S2, and more drastically in F2 (Figures 3C and 3D).

Because LETM1 dysfunction restricts mitochondrial respiratory capacity in yeast and mammals,[14,47] and the clinical and metabolic findings in the affected individuals were consistent with a mitochondrial disorder, we next investigated the abundance of the oxidative phosphorylation (OXPHOS) subunits. Fibroblasts of affected individuals

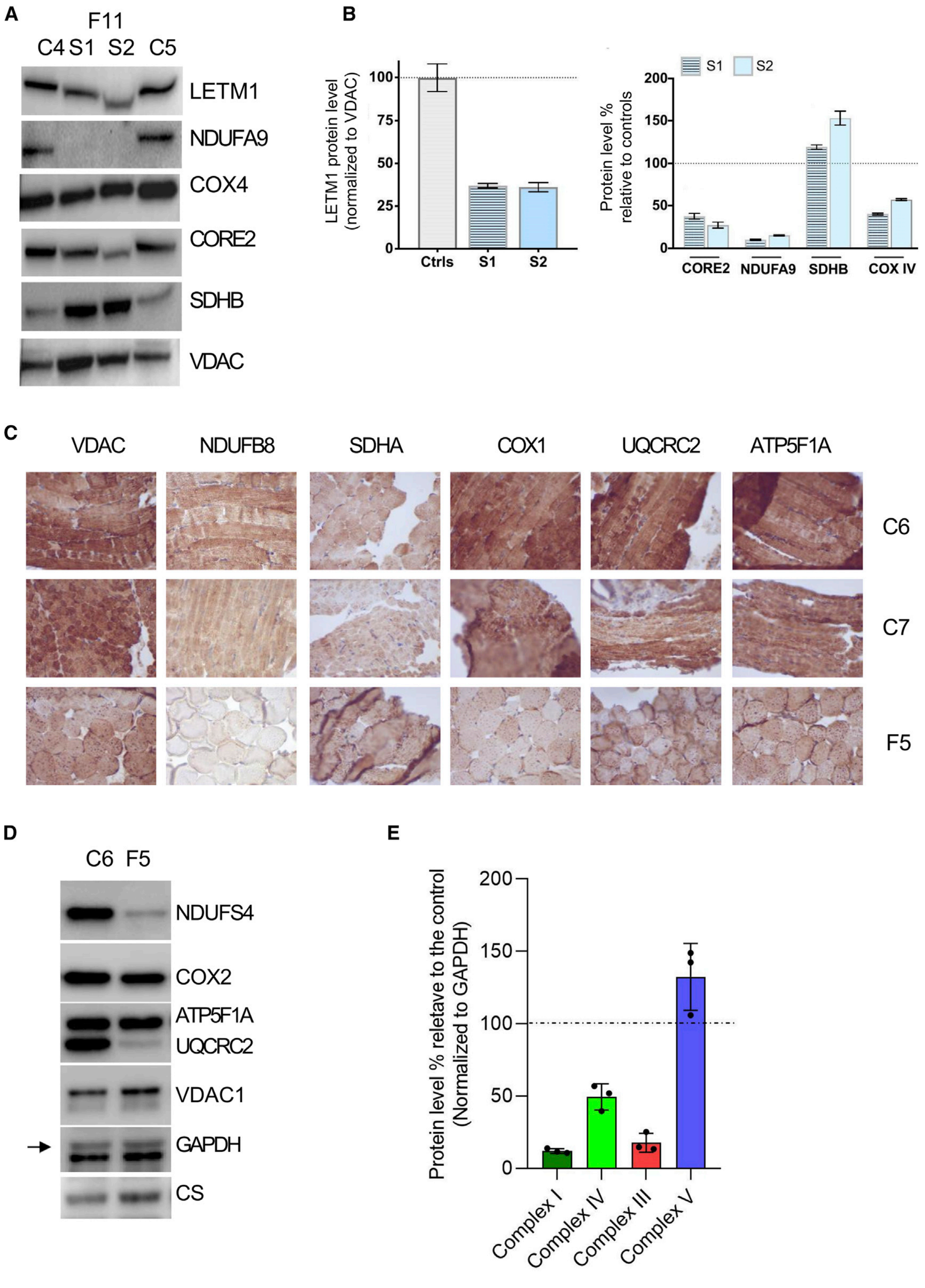


**Figure 4.** ***LETM1*** **variants affect the stability of LETM1 and OXPHOS components in muscle samples**
(A and B) Western blot analysis of LETM1 and components of the OXPHOS complexes I, II, III; and IV in muscle samples from F11 and quantitative graphs. Total lysates of muscle samples from healthy donors (C4, C5) and F11 c.898C>T (p.Pro300Ser) (S1, S2) were analyzed by immunoblotting using the indicated antibodies; VDAC served as a loading control (A). Quantitative graphs represent the protein levels relative to controls and normalized to VDAC. Data are expressed as mean ± SEM; n ≥ 3 independent experiments (B). (C) Immunohistochemical staining of OXPHOS subunits and VDAC of the muscle of F5 and control subjects. Muscle samples from healthy donors (C6, C7) and F5 c.1072G>A (p.Asp358Asn) were stained for each of the five OXPHOS subunits using the indicated antibodies; VDAC served as a control. Magnification 400×.

*(legend continued on next page)*

harboring bi-allelic *LETM1* variants displayed reduced steady-state levels of selected respiratory chain proteins of complex I and IV, in opposite to increased levels in F10 (c.2071−9C>G [p.Val691fs4*]) (Figures 3C and 3D). OXPHOS proteins NUDUFB8 and NDUFA9 were decreased in F1:S1 and F1:S2 fibroblasts and to a higher extent in F11:S2 (Figures 3C and 3D). Since mitochondrial defects can limit cellular growth, we assessed the proliferation rates of the fibroblast cell lines. While proliferation was comparable for fibroblasts with the single or compound heterozygous variants (F1), extension variant (F2), or wild-type *LETM1* (*LETM1* WT), it was significantly slowed down in *LETM1* c.2071−9C>G (p.Val691fs4*) (F10) and absent in *LETM1* c.1072G>A (p.Asp358Asn) (F5) fibroblasts (Figure S3).

Similar to fibroblasts, LETM1 was significantly reduced in the muscle of F11 (Figures 4A and 4B). NDUFA9 (complex I) was reduced in muscles samples from F11 while SDHB (complex II) displayed a strong tissue-specific upregulation (Figures 4A and 4B). The immunohistochemistry and western blotting analysis from F5 muscle tissue (Figures 4C–4E) revealed even greater reductions for components of complexes I, III, and IV, increased SDHA, accompanied by decreased enzyme activity of complex I, and upregulated activity of complex II and citrate synthase and increased mtDNA copy number (Table S2). Proteins of the ATP synthase remained not significantly changed in all tested cell lines and tissue samples.

Overall, altered LETM1 and OXPHOS protein levels in fibroblasts and muscle samples were observed in most of the individuals. Fibroblasts cell culture data indicated that bi-allelic *LETM1* variants result in aberrant mitochondrial morphology, which was more pronounced under galactose challenge (Figure S1B) and was often lethal for F11-derived fibroblasts. Consistent with the frequently observed effect of mitochondrial defects on cellular functions and growth, cell proliferation was retarded in F10 and more drastically in F5 fibroblasts. The synthetic KHE nigericin restored mitochondrial morphological aberrations and membrane depolarization, coupling mitochondrial dysfunctions and impaired $K^+$ homeostasis.

### Functional compensation analysis in yeast

Considering that LETM1 controls mitochondrial volume by regulating KHE, we ectopically expressed *LETM1* variants or wild-type in the *S. cerevisiae letm1*Δ strain to explore the functional impact of *LETM1* variants on mitochondrial KHE activity. All *LETM1* variants listed in the supplemental data were included in this analysis. The loss of KHE activity in yeast *letm1* deletion mutants, the complementation by re-expression of the homologous human *LETM1* WT, and the absence of a $Ca^{2+}$ transport system in *S. cerevisiae* mitochondria make the system ideally suited for functional complementation analysis of *LETM1* variants and determination of their pathogenicity with respect to KHE defects.

Light scattering recording of KOAc-induced swelling is a well-established method to measure the mitochondrial electroneutral exchange of $K^+$ for $H^+$.[48] Exposure of de-energized mitochondria to hypotonic KOAc buffer elicits the rapid uptake of protonated acetic acid, acidification of mitochondrial matrix, and thereby activation of KHE, which results in mitochondrial $K^+$ influx and water uptake and thus swelling.[45] Isolated mitochondria from *S. cerevisiae LETM1* wild-type cells and *S. cerevisiae letm1*Δ cells overexpressing human *LETM1* WT or variants or the empty control vector were subjected to KOAc-induced swelling experiments. Recording KHE activity by measuring the decrease in optical density (OD) using light scattering techniques allows discrimination of its main determinants: initial OD, indicating the osmotic state of mitochondria before KOAc addition, and KHE exchange rate per second, indicated by the amplitude from initial to final OD as a function of the time required to achieve it. As shown in Figure 5A, KOAc-induced swelling was sensitive to the KHE inhibitor quinine, confirming the correlation of optical density with KHE activity. Knockout of *S. cerevisiae LETM1* (*S. cerevisiae letm1*Δ) entirely abolished KHE activity, as illustrated by low initial OD and swelling amplitude, which were restored by expression of *LETM1* WT. The non-pathogenic variants (p.Ile305Leu and p.Lys587Arg) performed as well as *LETM1 WT* for the initial OD, and almost as well for the kinetics values. LETM1 with the variant p.Val691fs4* (F10) almost restored the initial OD, and so did *LETM1* p.Lys587Arg (F9) and p.Arg393His (F6) but their swelling amplitudes were very low. Expression of *LETM1* variants c.754–756del (p.Lys252del) (F3), c.878T>A (p.Ile293Asn) (F1:M), or c.2220G>C (p.*740Tyrext26) (F2, F7) marginally compensated $K^+$ fluxes with extremely slow swelling kinetics; swelling traces for *S. cerevisiae letm1*Δ transformed with *LETM1* c.881G>A (p.Arg294Gln) (F4, F8) or *LETM1* c.2094del (p.Asp699Metfs*13) (F1:F) suggested uncontrolled cation leakage (Figure 5A). Overexpression of *LETM1* c.1072G>A (p.Asp358Asn) did not rescue KHE. Taken together, these results suggest that mitochondrial reduced $K^+$ flux dynamics and swollen matrix are indicative of the functional impact of disease-associated *LETM1* variants.

LETM1 protein levels associated with *LETM1* variants were examined using total cell lysates and isolated mitochondria. In comparison to the mitochondrial loading control (Porin, Por1p), LETM1 total protein levels from ectopic *LETM1* WT or variant expression were similar, except those from F1:S1-S2 (*LETM1* c.[878T>A;

(D and E) Western blot analysis of subunits of the OXPHOS complexes, citrate synthase, and GAPDH of the muscle of F5 and control subjects. Total lysates of muscle samples were analyzed by immunoblotting using the indicated antibodies; VDAC, GAPDH, and CS served as loading controls. C6, healthy donor; F5, c.1072G>A (p.Asp358Asn) (D). Quantitative graphs representing the protein levels percentage relative to controls (normalized to GAPDH). n $\geq$ 3 independent experiments.

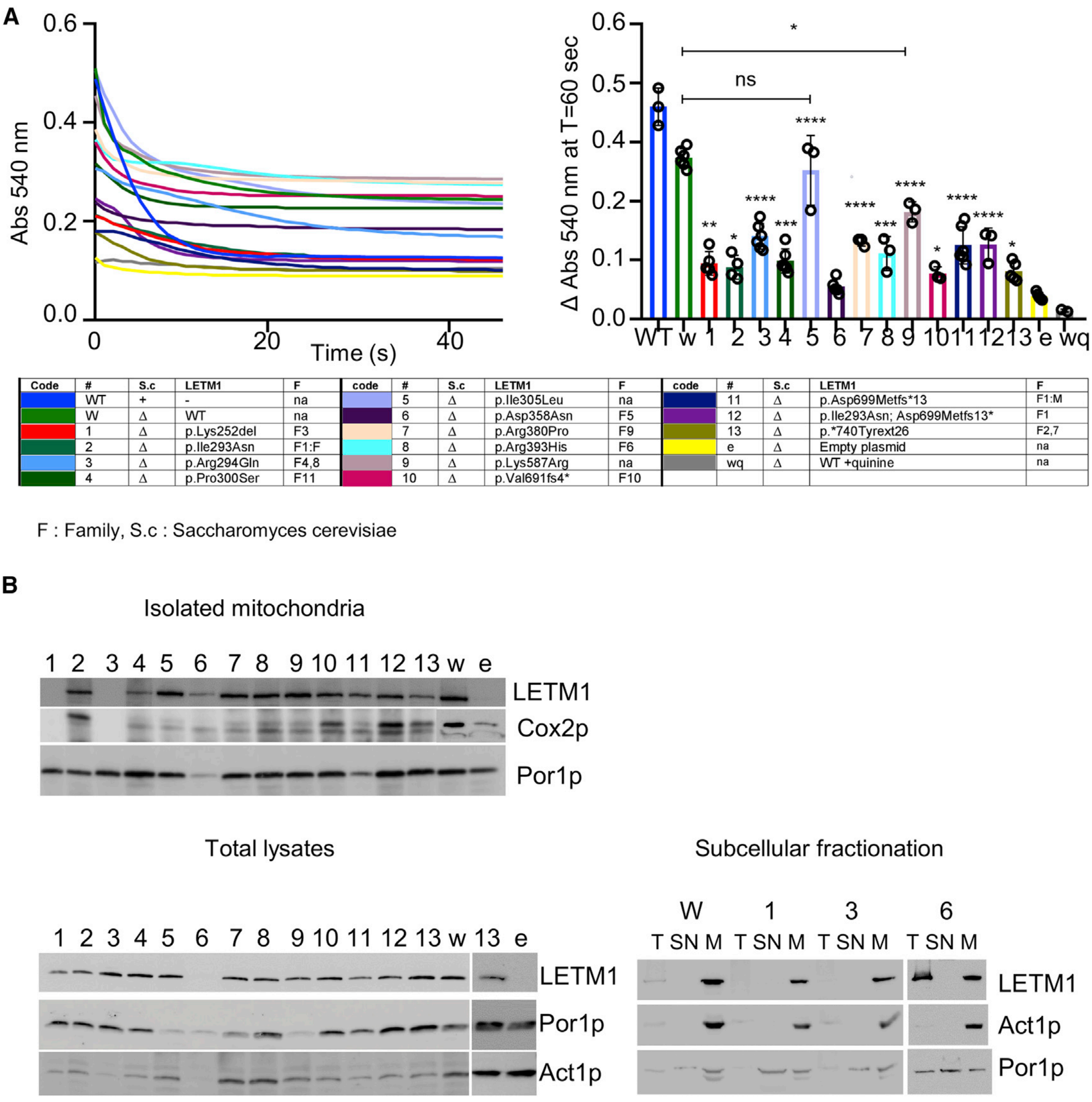


**Figure 5. Functional implication of *LETM1* variants on yeast mitochondria**

(A) *LETM1* variants fail to restore KHE activity of yeast *letm1*Δ. Isolated and de-energized mitochondria were subjected to KOAc and changes of optical density at $OD_{540}$ immediately measured. Left upper panel: representative traces of KOAc-induced swelling in *S. cerevisiae LETM1* WT mitochondria (WT, blue) or *S. cerevisiae letm1*Δ mitochondria overexpressing the empty plasmid (e, yellow) or the plasmid carrying human LETM1 WT untreated (w, green) or treated (wq, gray) with quinine or the human *LETM1* variants; color code as indicated in the inserted table: c.754–756del (p.Lys252del) (1, red), c.878T>A (p.Ile293Asn) (2, bottle green), c.881G>A (p.Arg294Gln) (3, aqua), c.898C>T (p.Pro300Ser) (4, dark green), c.913A>C (p.Ile305Leu) (5, lavender), c.1072G>A (p.Asp358Asn) (6, violet), c.1139G>C (p.Arg380Pro) (7, beige), c.1178G>A (p.Arg393His) (8, turquois), c.1760A>G (p.Lys587Arg) (9, mauve), c.2071−9C>G (p.Val691fs4*) (10, purple), c.2094del (p.Asp699Metfs*13) (11, dark blue), compound (12, lilac), c.2220G>C (p.*740Tyrext26) (13, olive). Right upper panel: quantified rates of KOAc-induced swelling amplitudes (t = 60 s) from 3 independent experiments. An overview of the swelling rate is given in Figure S5. One-way ANOVA with Dunnett's multiple comparisons test performed against *S. cerevisiae letm1*Δ transformed with empty pVT-103U plasmid *p = 0.0426, **p = 0.0026, ***p = 0.0006, ****p < 0.0001. And for p.Ile305Leu and p.Lys587Arg relatively to *S. cerevisiae letm1*Δ transformed with WT, ns > 0.05, *p = 0.0169.

(B) Ectopic expression of *LETM1* variants in *S. cerevisiae letm1* Δ. Isolated mitochondria (upper panel) and total protein lysates (left lower panel) from the same strains as in (A). Subcellular fractions (T, total; SN, post-mitochondrial supernatant; M, mitochondria) (right lower panel) were immunoblotted using the indicated antibodies; Por1p and Act1p served as mitochondrial and total (and SN) loading control, respectively.

2094del], p.[Ile293Asn; Asp699Metfs13*]), F2 and F7 (both *LETM1* c.2220G>C [p.*740Tyrext26]), and F11 (*LETM1* c.898C>T [p.Pro300Ser]), which showed reduced LETM1 levels in mitochondria (Figure 5B upper panel). The levels of LETM1 from the F5 *LETM1* variant (c.1072G>A [p.Asp358Asn]) were also low, but not when normalized to Por1p, which was similarly decreased (Figure 5B). LETM1 levels from the variants identified in F3 (c.754–756del [p.Lys252del]) and in F4 and F8 (both c.881G>A [p.Arg294Gln]) were detectable in total lysates and mitochondria prepared from a large-scale intracellular fractionation (Figure 5B right lower panel) but were also

reduced. None of the ectopic expression of *LETM1* variants, however, affected the mitochondrial subcellular localization.

As previously noticed[17,49,50] and shown here (Figure 5B), Cox2p (subunit of CIV) is reduced in *S. cerevisiae letm1Δ* strains. Cox2p levels were restored upon ectopic expression of human *LETM1* WT or *LETM1* c.878T>A (p.Ile293Asn) (F1:M), *LETM1* c.2071−9C>G (p.Val691fs4*) (F10), or *LETM1* c.[878T>A; 2094del], p.[Ile293Asn; Asp699 Metfs13*] (F1:S1, F1:S2), but remained absent upon expression of *LETM1* c.754–756del (p.Lys252del) (F3), *LETM1* c.881G>A (p.Arg294Gln) (F4, F8), or *LETM1* c.1072G>A (p.Asp358Asn) (F5) (Figure 5B).

*S. cerevisiae letm1Δ* shows poor growth on non-fermentable (YPG) substrate.[42] To determine the significance of the *LETM1* variants in rescuing the growth defects of *S. cerevisiae letm1Δ* compared to human *LETM1* WT, serial dilutions of *S. cerevisiae letm1Δ* strains overexpressing an empty plasmid or *LETM1* variants or WT were spotted onto fermentable (YPD) and non-fermentable (YPG) plates and grown at 30°C or 37°C (Figure S4). We found a detrimental effect of the mutant phenotype by ectopic expression of *LETM1* c.1072G>A (p.Asp358Asn) (F5) variant; this strain was able to grow on selective media but showed worsened growth defect on complete media. Growth was also slowed down at 37°C on YPD by *LETM1* c.898C>T (p.Pro300Ser)) (F11). On YPG, a marginal rescue was obtained by ectopic expression of *LETM1* c.881G>A (p.Arg294Gln) (F4, F8), *LETM1* c.2071−9C>G (p.Val691fs4*) (F10), or LETM1 c.[878T>A; 2094del], p.[Ile293Asn; Asp699Metfs13*] (F1:S1, F1:S2) variants.

In summary, ectopic expression in *S. cerevisiae letm1Δ* of human *LETM1* variants associated with clinical presentations phenocopied *S. cerevisiae letm1* loss of function, whereas expression of wild-type LETM1 restored the yeast deletion defects in non-fermentable growth and mitochondrial KHE exchange.

## Discussion

LETM1 function is required for the maintenance of mitochondrial cationic and osmotic balance, and swelling of the matrix due to impaired LETM1 has far-reaching consequences. Matrix swelling is supported by the unfolding of inner membranes and loss of cristae invaginations and results in dilution of metabolic substrates. Here, we found that bi-allelic *LETM1* variants identified in the affected individuals with severe clinical features differently affected the LETM1 levels and led to the typical aberrant mitochondrial morphology previously described for LETM1-deficient cells. Several OXPHOS subunits were downregulated in fibroblasts or muscle tissue, enzymatic activities were reduced, and mtDNA copy number increased. The fact that nigericin, the synthetic KHE, restored morphological aberrations interconnects these phenotypes to impaired $K^+$ homeostasis. Decreased membrane potential or increased sensitivity to valinomycin and normalization of this sensitivity by nigericin supports the presence of a defect in $K^+/H^+$ exchange. Moreover, it is tempting to speculate that OXPHOS decreases proportionally to cristae loss. The finding that loss of KHE activity in *LETM1*-defective yeast was restored by ectopic expression of wild-type *LETM1* but not *LETM1* variants strongly support the notion of deregulated mitochondrial $K^+$ homeostasis caused by the *LETM1* variants. Whether and how $Ca^{2+}$ handling is also perturbed will need to be determined in future studies. Together with the fibroblasts, muscle biopsy, and yeast analyses, and with the prior knowledge that the mitochondrial phenotypes in cells match those caused by LETM1 haploinsufficiency, knockdown, or deletion in other eukaryotic species, the present findings amount to compelling evidence that the bi-allelic *LETM1* variants are the cause of the disease in the pedigrees reported in this study.

### Diseases of mitochondrial morphology and dynamics

Defects in non-OXPHOS genes responsible for mitochondrial homeostasis including mitochondrial fission and fusion have been suggested to cause primary MD (PMD).[51] Primarily targeting the non-bioenergetic capabilities of the mitochondria, non-OXPHOS gene defects could indirectly affect the OXPHOS system,[52] leading to a phenotype mimicking the inactivation of RCE.[12]

While there is a plethora of non-OXPHOS genes accounting for PMD,[51,53,54] the examples relevant to the context of the present study are the genes regulating mitochondrial shape and interorganellar communication. They regulate mitochondrial dynamics through fusion and fission processes. Defects in these genes have been emerging as a cause of a novel class of inherited neurodegenerative disorders with variable onset ranging from infancy to adulthood.[53,54] Residing in the outer and inner mitochondrial membranes or the cytosol, upon misregulation, they cause altered mitochondrial morphology including matrix swelling, fragmentation, elongation, and abnormal cristae structure, similar to what has been observed in abnormal LETM1 function.[53–56] Reviews of the disease-causing genes responsible for mitochondrial dynamics are provided in Burté et al.[53] and Navaratnarajah et al.[54] To date, affected individuals diagnosed with diseases of mitochondrial dynamics present first and foremost with neurological symptoms.[53,54] Being essential for the survival of all organisms tested so far and having important control over the mitochondrial osmotic balance, morphology, and dynamics, before now, bi-allelic variants in *LETM1* have not been associated with any Mendelian disorder in humans.

### Bi-allelic *LETM1* variants present with a phenotypic spectrum of MD largely involving the CNS

Here we report on the association of bi-allelic *LETM1* variants with a spectrum of predominantly infantile-onset neurological, metabolic, dysmorphic, and multiple organ

dysfunction syndromes in a cohort of 18 affected individuals from 11 unrelated families. Overall, the disease had a progressive course, though with variable rates of deterioration. Hence, the disease progression varied from rapid (as in families 3, 4, and 9–11) to a slow deterioration (as in the oldest persons from families 1, 2, and 8). Similar to the clinical presentation of the defective mitochondrial dynamics genes, bi-allelic *LETM1* variants were associated with an infantile-onset neurodegenerative disorder with a complex phenotype as described for *DNM1L*/*DRP1* (Dynamin 1 like [MIM: 603850]), *OPA1* (OPA1 mitochondrial dynamin-like GTPase [MIM: 605290]), *OPA3* (Outer mitochondrial membrane lipid metabolism regulator OPA3 [MIM: 606580]), *MFF* (Mitochondrial fission factor [MIM: 614785]), and *MSTO1* (Misato mitochondrial distribution and morphology regulator 1 [MIM: 617619]).[53–56] The shared phenotype mainly included global developmental delay, regression, and neurosensory impairment combined with neuromuscular symptoms, cerebellar ataxia, seizures, and early mortality. Akin to defects in *OPA3*, 3-methylglutaconic aciduria was a frequent finding in the subjects with bi-allelic *LETM1* variants.[57] Bilateral cataracts and facial dysmorphism observed in the present *LETM1* cohort have also been reported in individuals with defective *OPA3* and *MSTO1*, respectively.[55,56]

All persons with RCE analysis results in the present study showed defects in the OXPHOS system suggesting that *LETM1* defects can affect the mitochondrial ability to generate ATP. This in turn might have mimicked the clinical presentation of OXPHOS MD. Therefore, distinguishing the *LETM1* phenotype from OXPHOS MD or the aforementioned diseases of mitochondrial dynamics can be challenging without the help of genetic testing, particularly in affected individuals with a rapidly progressive disease course.

## The phenotype of defective *LETM1* and WHS

Monoallelic *LETM1* deletion has been suggested to be responsible for epilepsy and neuromuscular features in WHS.[5,19,21,58] Indeed, the current *LETM1* cohort presented with hypotonia and epilepsy. Additionally, though, persons with bi-allelic *LETM1* variants showed a milder spectrum of WHS signs that has not been previously ascribed to the *LETM1* deletion. These included thin habitus, low set ears, microcephaly, micrognathia, and low body weight.[59,60] It has been previously speculated that the most probable cause of growth deficiency, microcephaly, and the characteristic facial features in WHS is due to haploinsufficiency of *WHSC1*, a region located far from *LETM1*.[61] The expression of mild non-neurological symptoms of WHS in our cohort could be due to either putative interaction between *LETM1* and *WHSC1* or other undiscovered mechanisms, including those intrinsically caused by *LETM1* deficiencies.

We have observed some degree of clinical overlap between the presentation of defective *LETM1* and small interstitial deletions in WHS presenting with a milder phenotype. The latter presents with a variable degree of growth and neurodevelopmental delay, microcephaly, thin faces with dysmorphic features, intellectual disability, language impairment, and seizures.[62–64] Interestingly, persons with small 4p16.3 deletions encompassing *LETM1* suggested that *LETM1* might not be responsible for seizures in WHS as some individuals with *LETM1* deletion did not have seizures by the age of 4 and 9 years, whereas persons with preserved WSHCR-2 including *LETM1* developed seizures.[64] Previous retrospective analysis suggests that several other genes in the terminal 4p region might potentially be involved in seizures in WHS.[5]

Clinical features including lactic acidosis, diabetes, cataract, neuropathy, and proximal myopathy combined with cerebellar ataxia, progressive spastic-ataxic gait, hyperkinetic movement disorders, and pontine/cerebellar atrophy were among the signs of the defective *LETM1* phenotype that are not typical of WHS; instead, they are more typical of archetypal mitochondrial disorders.

Although there have been a handful of reports on microdeletions in WHS describing genotype-phenotype correlations, the association between the specific symptoms of WHS and *LETM1* remains to be fully determined. To understand the full contribution of *LETM1* in WHS cases, further studies would be needed to investigate which phenotypes of WHS can be restored by the re-expression of *LETM1*. Apart from this, the identification of phenotypes that were consistent with both *LETM1* haploinsufficiency in WHS and *LETM1* bi-allelic variants will advance our understanding of the contribution of *LETM1* in WHS.

## Genotype-phenotype correlation of bi-allelic *LETM1* variants

The general distribution of the missense and frameshift variants to the highly conserved LETM domain and the C-terminal coiled coils, together with their comparable deleterious effects on mitochondrial morphology and KHE function, support the correlation of mitochondrial morphologic defects and imbalanced cation homeostasis. A previous variant analysis of the LETM domain found that Asp359 or the triple combination of Arg382, Gly383, and Met384 is necessary for the organization of cristae structure and growth complementation of *S. cerevisiae letm1Δ* strains.[65] The missense variant c.1072G>A (p.Asp358Asn) identified here in family 5, which impaired mitochondrial morphology and KHE activity, is adjacent to Asp359. Based on cell-free data showing that the reconstituted LETM domain was sufficient to induce cristae invagination, Nakamura et al.[65] concluded that cristae disorganization due to the single or triple variant occurred independently of ion homeostasis. Our findings are not in contradiction but propose that a regulatory contribution to cristae architecture by the LETM domain may depend on the swelling state of mitochondria in the cellular context.

Given the growing consensus that the hallmark of LETM1 deficiency is mitochondrial cation imbalance, we

used yeast as a model organism to analyze mitochondrial KHE activity of *LETM1* variants from affected individuals and *LETM1* variants not associated with the disease. Based on the results, we propose that light-scattering experiments that capture mitochondrial volume status and kinetics of $K^+/H^+$ exchange are useful to predict the pathogenic potential of *LETM1* variants (Figure S5).

Linking clinical features with *in vitro* data, we found that fibroblasts expressing *LETM1* variants c.[878T>A; 2094del], p.[Ile293Asn; Asp699Metfs13*], which were identified in the individuals F1:S1 and F1:S2 affected with epilepsy, neurosensory deficiencies, and diabetes, displayed mitochondria with disturbed morphology and membrane potential, reduced LETM1 levels, and a severe decrease in respiratory proteins of CI and CIV. Ectopic expression of the variants in yeast marginally rescued mitochondrial KHE activity. Persons harboring the variant c.2071−9C>G (p.Val691fs4*) (F10) showed rapid clinical progression and died before reaching 1 year of age. Fibroblasts from this person displayed high LETM1 protein levels, indicating that the pathogenic variant and not the lack of protein was associated with the severe phenotypes. Ectopic expression of this variant failed to rescue wild-type KHE activity. The abundance of this non-functional *LETM1* variant suggests that it likely escaped the nonsense-mediated decay as the gained stop codon falls into the last exon.[66] The variant c.898C>T (p.Pro300Ser) was identified in family 11 leading to a severe early infantile disease in the homozygous state. Fibroblasts and muscle lysates from those individuals showed reduced CI and CIV proteins. Drastic growth defects and lack of KHE activity were induced by this variant in yeast, which could somehow explain the severe clinical conditions caused by this variant. *LETM1* c.2220G>C (p.*740Tyrext26) was identified in several subjects from F2 and F7 with developmental delay, walking difficulties, and seizures. Fibroblasts from F2:S1 exhibited swollen and fragmented mitochondria and hardly detectable LETM1 protein levels. Ectopic expression in yeast displayed somewhat reduced LETM1 protein levels and poorly improved KHE activity. Since the KHE uses the proton gradient generated by the respiratory chain to drive $K^+$ flux, and LETM1 is likely involved in the insertion of mitochondrial encoded OXPHOS proteins into the membrane, it is surprising that the reduction of this LETM1 variant did not correlate with decreased OXPHOS components. There are several possible explanations for this. The OXPHOS damages could be secondary to LETM1 deficiency, the OXPHOS components (although not reduced) may not assemble as efficiently, or genetic compensatory mechanisms are involved. The affected individual carrying the homozygous variant *LETM1* c.1072G>A (p.Asp358Asn) (F5) presented defects in neurosensory functions and type 3 diabetes. We found severely impaired proliferation of F5-derived fibroblasts. Similarly, yeast growth was also repressed by this variant, and mitochondrial KHE activity could not be restored. Compared to the other variants, c.1072G>A (p.Asp358Asn) had the most deleterious effects on mitochondrial morphology, cell proliferation, and KHE activity, predicting this variant to have the most severe consequences. However, the viable state of the affected individual also here raises the possibility of a potential genetic compensatory background. In this respect, increased mtDNA copy number—often considered as an efficient way to overcome OXPHOS deficiencies in diseases and aging[67]—or elevated citrate synthase activity found in muscle specimens may indicate such a compensatory pathway (Figure 4C). Further examination will be required to clarify molecular compensatory mechanisms.

Other *LETM1* variants were analyzed in yeast, as fibroblasts from affected individuals were not available. Yeast data revealed poor complementation of *S. cerevisiae letm1*Δ by human *LETM1* c.754–756del (p.Lys252del), a variant identified in affected individuals with a neurological, neuromuscular, and craniofacial presentation, rapid progression, and eventually death (F3). Ectopic expression of *LETM1* c.881G>A (p.Arg294Gln), identified in persons with variable disease progression (F4, F8) but similar neuromuscular deficiencies, was not able to restore the activity of the mitochondrial KHE, since the swelling traces revealed continuous but very slow kinetics indicating minimal KHE activity per time unit, thus suggesting leaky mitochondrial membranes. Yeast growth was also impaired by overexpression of this variant. Phenotypic data were rather consistent with severe clinical presentation and early demise in F4.

The affected individual from family 9 was homozygous for the *LETM1* variant c.1139G>C (p.Arg380Pro) and presented respiratory insufficiency, epileptic encephalopathy, neuromuscular disorder, and rapid disease progression. The missense variant is located in the middle of the LETM domain, in proximity to the three highly conserved amino acid residues (Arg382, Gly383, Met384) described in Nakamura et al.[65] (Figure 2B), supporting an essential functional role of the LETM stretch between residues 380 and 384.

### LETM1 role in cation homeostasis and neurodegenerative phenotype of the cohort

Among the mitochondrial EF-hand-containing proteins, LETM1 has been identified as essential across several cell lines in genome-wide essentiality screens.[68,69] Functionally, LETM1 is required for maintaining mitochondrial homeostasis of $K^+$ and was considered an essential component of the KHE. After LETM1 was identified in a genomic *Drosophila* RNAi screen for mitochondrial $Ca^+/H^+$ exchanger (CHX), it has been suggested to catalyze the exchange of $Ca^{2+}$ against $H^+$ in both directions in a ruthenium red-sensitive pattern,[70] which is difficult to reconcile with the CHX, and has been implicated in the pathogenesis of Parkinson's disease through interaction with *PINK1* (PTEN-induced kinase 1 [MIM: 608309]).[71] The mitochondrial CHX is part of the mitochondrial $Ca^{2+}$ release system, which compensates for electrophoretic mitochondrial $Ca^{2+}$ uptake mainly through $H^+$- or $Na^+$-dependent $Ca^{2+}$ extrusion. While

the role of LETM1 as a mitochondrial KHE or CHX has remained controversial, deregulation of the mitochondrial KHE has been shown to affect mitochondrial $Ca^{2+}$ buffering by impacting the $Na^{+}$-dependent $Ca^{2+}$ release pathway.[72] Proper maintenance of mitochondrial $Ca^{2+}$ levels is critical to neurons, synaptic function, and neurodevelopment with mishandled mitochondrial $Ca^{2+}$ levels posing a risk of synaptopathies. In turn, synaptopathies may be a harbinger of neurodegenerative disorders.[73] The neurodegenerative phenotype observed in the present *LETM1* cohort could partially be explained by impaired mitochondrial $Ca^{2+}$ buffering and ensuing glutamate excitotoxicity, generation of reactive oxygen species, and apoptosis.[74] Consistent with previous studies,[13,14,50,75] exposure to nigericin or ketone bodies improved the mitochondrial morphological phenotypes of fibroblasts from affected individuals, supporting the link between *LETM1* variant and impaired cation homeostasis. While nigericin enables $K^{+}$-$H^{+}$ exchange and prevents accumulation of matrix $K^{+}$, ketone bodies may bypass the deficient $Ca^{2+}$-dependent catalytic function of the pyruvate dehydrogenase.

Unlike in yeast, LETM1 orthologs of more complex organisms possess EF-hands, which may implicate LETM1 in $Ca^{2+}$ sensing or regulation.[76] Focusing on $K^{+}$ analysis using yeast, we did not investigate the impact of the reported bi-allelic *LETM1* variants on the mitochondrial $Ca^{2+}$ homeostasis. This would need to be investigated in further studies as it might have future therapeutic implications.[73]

Collectively, our results demonstrate that bi-allelic pathogenic *LETM1* variants are associated with defective mitochondrial $K^{+}$ efflux, swollen mitochondrial matrix structures, and a reduction in proteins levels and activity of the electron transfer chain. The former highlights the implication of perturbed mitochondrial osmoregulation caused by bi-allelic *LETM1* variants in neurological and mitochondrial pathologies. Data showing that mitochondrial KHE activity is maintained above a functional threshold in non-pathogenic variants suggest that such functional yeast assays could be implemented to routinely determine the pathogenicity of a variant. While the beneficial effect of nigericin strengthened the link to KHE defects, that of ketone bodies, consistent with Durigon et al.,[14] supports the promising therapeutic role of ketogenic-based diets.

## Data and code availability

The accession numbers for the genetic variants reported in this paper are ClinVar: SCV001981656, SCV001981657, SCV001981658, SCV001981659, SCV001981660, SCV001981661, SCV001981662, SCV001981663, SCV001981664, and SCV001981665.

## Supplemental information

Supplemental information can be found online at https://doi.org/10.1016/j.ajhg.2022.07.007.


## Acknowledgments

The authors would like to thank the affected individuals and their families for their support of this study. We thank Dr. Franz Klein and his lab for support in yeast transformation and generous supply of yeast media and Ronald Mekis for help with statistics. This research was supported using resources of the Core Facility Cell Imaging and Ultrastructure Research, University of Vienna, a member of the Vienna Life-Science Instruments (VLSI) and the VetCore Facility (Imaging) of the University of Veterinary Medicine Vienna. We acknowledge International Centre for Genomic Medicine in Neuromuscular Diseases. This research was funded in part, by the Wellcome Trust (WT093205MA, WT104033AIA, and the Synaptopathies Strategic Award, 165908). This study was funded by the Medical Research Council (MR/S01165X/1, MR/S005021/1, G0601943), The National Institute for Health Research University College London Hospitals Biomedical Research Centre, Rosetrees Trust, Ataxia UK, Multiple System Atrophy Trust, Brain Research United Kingdom, Sparks Great Ormond Street Hospital Charity, Muscular Dystrophy United Kingdom (MDUK), Muscular Dystrophy Association (MDA USA) and Senior Non-Clinical Fellowship to A. Spinazzola, (MC_PC_13029). K.N. and S.E.M.M. were supported by the Austrian Science Funds FWF-P29077 and P31471. A. Spinazzola receives support also from The Lily Foundation and Brain Research UK. R.K. was supported by European Academy of Neurology Research Training Fellowship and Rosetrees Trust PhD Plus award (PhD2022\100042). For the purpose of Open Access, the author has applied a CC BY public copyright license to any Author Accepted Manuscript version arising from this submission. Further acknowledgments are available in the supplemental information.


## Declaration of interests

The authors declare no competing interests.


Received: March 27, 2022
Accepted: July 1, 2022
Published: September 1, 2022


## Web resources

Brain RNA-seq, http://www.brainrnaseq.org/
ClinVar, https://www.ncbi.nlm.nih.gov/clinvar/
ClinVar (summary of submissions), https://www.ncbi.nlm.nih.gov/clinvar/submitters/26957
Database of Genome Variants, http://dgv.tcag.ca/
Ensemble, https://www.ensembl.org/index.html
FlyBase, http://flybase.org/
GeneMatcher, https://genematcher.org/
gnomAD, https://gnomad.broadinstitute.org/
Iranome, http://www.iranome.ir/
OMIM, https://www.omim.org/
TOPMed, https://bravo.sph.umich.edu/freeze8/hg38/gene/snv/LETM1
UKBB, https://www.ukbiobank.ac.uk/
Uniprot, https://www.uniprot.org/
Varnomen, http://varnomen.hgvs.org/
Varsome, https://varsome.com/

## References


1. Gyimesi, G., and Hediger, M.A. (2020). Sequence features of mitochondrial transporter protein families. Biomolecules *10*, 1611.
2. Nowikovsky, K., Pozzan, T., Rizzuto, R., Scorrano, L., and Bernardi, P. (2012). Perspectives on: SGP symposium on mitochondrial physiology and medicine: the pathophysiology of LETM1. J. Gen. Physiol. *139*, 445–454.
3. Hajnóczky, G., Booth, D., Csordás, G., Debattisti, V., Golenár, T., Naghdi, S., Niknejad, N., Paillard, M., Seifert, E.L., and Weaver, D. (2014). Reliance of ER-mitochondrial calcium signaling on mitochondrial EF-hand Ca2+ binding proteins: Miros, MICUs, LETM1 and solute carriers. Curr. Opin. Cell Biol. *29*, 133–141.
4. Endele, S., Fuhry, M., Pak, S.J., Zabel, B.U., and Winterpacht, A. (1999). LETM1, a novel gene encoding a putative EF-hand Ca(2+ )-binding protein, flanks the Wolf-Hirschhorn syndrome (WHS) critical region and is deleted in most WHS patients. Genomics *60*, 218–225.
5. Austin, S., and Nowikovsky, K. (2019). LETM1: essential for mitochondrial biology and cation homeostasis? Trends Biochem. Sci. *44*, 648–658.
6. Lin, Q.T., and Stathopulos, P.B. (2019). Molecular mechanisms of leucine zipper EF-hand containing transmembrane protein-1 function in health and disease. Int. J. Mol. Sci. *20*, E286.
7. Waldeck-Weiermair, M., Jean-Quartier, C., Rost, R., Khan, M.J., Vishnu, N., Bondarenko, A.I., Imamura, H., Malli, R., and Graier, W.F. (2011). Leucine zipper EF hand-containing transmembrane protein 1 (Letm1) and uncoupling proteins 2 and 3 (UCP2/3) contribute to two distinct mitochondrial Ca 2+ uptake pathways. J. Biol. Chem. *286*, 28444–28455.
8. Froschauer, E., Nowikovsky, K., and Schweyen, R.J. (2005). Electroneutral K+/H+ exchange in mitochondrial membrane vesicles involves Yol027/Letm1 proteins. Biochim. Biophys. Acta *1711*, 41–48.
9. Jiang, D., Zhao, L., Clish, C.B., and Clapham, D.E. (2013). Letm1, the mitochondrial Ca 2+/H + antiporter, is essential for normal glucose metabolism and alters brain function in Wolf-Hirschhorn syndrome. Proc. Natl. Acad. Sci. USA *110*, E2249–E2254.
10. Shao, J., Fu, Z., Ji, Y., Guan, X., Guo, S., Ding, Z., Yang, X., Cong, Y., and Shen, Y. (2016). Leucine zipper-EF-hand containing transmembrane protein 1 (LETM1) forms a Ca2+/H+ antiporter. Sci. Rep. *6*, 34174.
11. Hasegawa, A., and van der Bliek, A.M. (2007). Inverse correlation between expression of the Wolfs Hirschhorn candidate gene Letm1 and mitochondrial volume in C. elegans and in mamma- lian cells. Hum. Mol. Genet. *16*, 2061–2071.
12. McQuibban, A.G., Joza, N., Megighian, A., Scorzeto, M., Zanini, D., Reipert, S., Richter, C., Schweyen, R.J., and Nowikovsky, K. (2010). A Drosophila mutant of LETM1, a candidate gene for seizures in Wolf-Hirschhorn syndrome. Hum. Mol. Genet. *19*, 987–1000.
13. Dimmer, K.S., Navoni, F., Casarin, A., Trevisson, E., Endele, S., Winterpacht, A., Salviati, L., and Scorrano, L. (2008). LETM1, deleted in Wolf-Hirschhorn syndrome is required for normal mitochondrial morphology and cellular viability. Hum. Mol. Genet. *17*, 201–214.
14. Durigon, R., Mitchell, A.L., Jones, A.W., Manole, A., Mennuni, M., Hirst, E.M., Houlden, H., Maragni, G., Lattante, S., Doronzio, P.N., et al. (2018). .LETM 1 couples mitochondrial DNA metabolism and nutrient preference. EMBO Mol. Med. *10*, 1–20.
15. Piao, L., Li, Y., Kim, S.J., Byun, H.S., Huang, S.M., Hwang, S.K., Yang, K.J., Park, K.A., Won, M., Hong, J., et al. (2009). Association of LETM1 and mrpl36 contributes to the regulation of mitochondrial ATP production and necrotic cell death. Cancer Res. *69*, 3397–3404.
16. Kuum, M., Veksler, V., Liiv, J., Ventura-Clapier, R., and Kaasik, A. (2012). Endoplasmic reticulum potassium-hydrogen exchanger and small conductance calcium-activated potassium channel activities are essential for ER calcium uptake in neurons and cardiomyocytes. J. Cell Sci. *125*, 625–633.
17. Lupo, D., Vollmer, C., Deckers, M., Mick, D.U., Tews, I., Sinning, I., and Rehling, P. (2011). Mdm38 is a 14-3-3-like receptor and associates with the protein synthesis machinery at the inner mitochondrial membrane. Traffic *12*, 1457–1466.
18. Li, Y., Tran, Q., Shrestha, R., Piao, L., Park, S., Park, J., and Park, J. (2019). LETM1 is required for mitochondrial homeostasis and cellular viability (Review). Mol. Med. Rep. *19*, 3367–3375.
19. Schlickum, S., Moghekar, A., Simpson, J.C., Steglich, C., O'Brien, R.J., Winterpacht, A., and Endele, S.U. (2004). LETM1, a gene deleted in Wolf-Hirschhorn syndrome, encodes an evolutionarily conserved mitochondrial protein. Genomics *83*, 254–261.
20. Hart, L., Rauch, A., Carr, A.M., Vermeesch, J.R., and O'Driscoll, M. (2014). LETM1 haploinsufficiency causes mitochondrial defects in cells from humans with Wolf-Hirschhorn syndrome: implications for dissecting the underlying pathomechanisms in this condition. Dis. Model. Mech. *7*, 535–545.
21. Zhang, X., Chen, G., Lu, Y., Liu, J., Fang, M., Luo, J., Cao, Q., and Wang, X. (2014). Association of mitochondrial letm1 with epileptic seizures. Cereb. Cortex *24*, 2533–2540.
22. Park, J., Li, Y., Kim, S.H., Yang, K.J., Kong, G., Shrestha, R., Tran, Q., Park, K.A., Jeon, J., Hur, G.M., et al. (2014). New players in high fat diet-induced obesity: LETM1 and CTMP. Metabolism *63*, 318–327.
23. Sobreira, N., Schiettecatte, F., Valle, D., and Hamosh, A. (2015). GeneMatcher: a matching tool for connecting investigators with an interest in the same gene. Hum. Mutat. *36*, 928–930.
24. Catania, A., Legati, A., Peverelli, L., Nanetti, L., Marchet, S., Zanetti, N., Lamperti, C., and Ghezzi, D. (2019). Homozygous variant in OTX2 and possible genetic modifiers identified in a patient with combined pituitary hormone deficiency, ocular involvement, myopathy, ataxia, and mitochondrial impairment. Am. J. Med. Genet. *179*, 827–831.
25. Horga, H.A. (2019). Clinical and Genetic Investigations of Inherited Neuropathies and Mitochondrial Disease. PhD thesis (University College London).
26. Makrythanasis, P., Maroofian, R., Stray-Pedersen, A., Musaev, D., Zaki, M.S., Mahmoud, I.G., Selim, L., Elbadawy, A., Jhangiani, S.N., Coban Akdemir, Z.H., et al. (2018). Biallelic variants in KIF14 cause intellectual disability with microcephaly. Eur. J. Hum. Genet. *26*, 330–339.
27. Poole, O.V., Pizzamiglio, C., Murphy, D., Falabella, M., Macken, W.L., Bugiardini, E., Woodward, C.E., Labrum, R., Efthymiou, S., Salpietro, V., et al. (2021). Mitochondrial DNA analysis from exome sequencing data improves diagnostic yield in neurological diseases. Ann. Neurol. *89*, 1240–1247.
28. Barington, M., Risom, L., Ek, J., Uldall, P., and Ostergaard, E. (2018). A recurrent de novo CUX2 missense variant associated

with intellectual disability, seizures, and autism spectrum disorder. Eur. J. Hum. Genet. *26*, 1388–1391.
29. Van Bergen, N.J., Guo, Y., Rankin, J., Paczia, N., Becker-Kettern, J., Kremer, L.S., Pyle, A., Conrotte, J.F., Ellaway, C., Procopis, P., et al. (2019). NAD(P)HX dehydratase (NAXD) deficiency: a novel neurodegenerative disorder exacerbated by febrile illnesses. Brain *142*, 50–58.
30. Froukh, T., Nafie, O., Al Hait, S.A.S., Laugwitz, L., Sommerfeld, J., Sturm, M., Baraghiti, A., Issa, T., Al-Nazer, A., Koch, P.A., et al. (2020). Genetic basis of neurodevelopmental disorders in 103 Jordanian families. Clin. Genet. *97*, 621–627.
31. Retterer, K., Juusola, J., Cho, M.T., Vitazka, P., Millan, F., Gibellini, F., Vertino-Bell, A., Smaoui, N., Neidich, J., Monaghan, K.G., et al. (2016). Clinical application of whole-exome sequencing across clinical indications. Genet. Med. *18*, 696–704.
32. Bai, R., Cui, H., Devaney, J.M., Allis, K.M., Balog, A.M., Liu, X., Schnur, R.E., Shapiro, F.L., Brautbar, A., Estrada-Veras, J.I., et al. (2021). Interference of nuclear mitochondrial DNA segments in mitochondrial DNA testing resembles biparental transmission of mitochondrial DNA in humans. Genet. Med. *23*, 1514–1521.
33. Richards, S., Aziz, N., Bale, S., Bick, D., Das, S., Gastier-Foster, J., Grody, W.W., Hegde, M., Lyon, E., Spector, E., et al. (2015). ACMG laboratory quality assurance committee. Standards and guidelines for the interpretation of sequence variants: a joint consensus recommendation of the American College of medical genetics and genomics and the association for molecular pathology. Genet. Med. *17*, 405–424.
34. Williamson, S.L., Rasanayagam, C.N., Glover, K.J., Baptista, J., Naik, S., Satodia, P., and Gowda, H. (2021). Rapid exome sequencing: revolutionises the management of acutely unwell neonates. Eur. J. Pediatr. *180*, 3587–3591.
35. Kremer, L.S., Bader, D.M., Mertes, C., Kopajtich, R., Pichler, G., Iuso, A., Haack, T.B., Graf, E., Schwarzmayr, T., Terrile, C., et al. (2017). Genetic diagnosis of Mendelian disorders via RNA sequencing. Nat. Commun. *8*, 15824.
36. Calvo, S.E., Compton, A.G., Hershman, S.G., Lim, S.C., Lieber, D.S., Tucker, E.J., Laskowski, A., Garone, C., Liu, S., Jaffe, D.B., et al. (2012). Molecular diagnosis of infantile mitochondrial disease with targeted next-generation sequencing. Sci. Transl. Med. *4*, 118ra10.
37. Legati, A., Reyes, A., Nasca, A., Invernizzi, F., Lamantea, E., Tiranti, V., Garavaglia, B., Lamperti, C., Ardissone, A., Moroni, I., et al. (2016). New genes and pathomechanisms in mitochondrial disorders unraveled by NGS technologies. Biochim. Biophys. Acta *1857*, 1326–1335.
38. Saoura, M., Powell, C.A., Kopajtich, R., Alahmad, A., Al-Balool, H.H., Albash, B., Alfadhel, M., Alston, C.L., Bertini, E., and Bonnen, P.E. (2019). Mutations in ELAC2 associated with hypertrophic cardiomyopathy impair mitochondrial tRNA 3'-end processing. Hum. Mutat. *40*, 1731–1748.
39. Kušíková, K., Feichtinger, R.G., Csillag, B., Kalev, O.K., Weis, S., Duba, H.C., Mayr, J.A., and Weis, D. (2021). Case report and review of the literature: a new and a recurrent variant in the VARS2 gene are associated with isolated lethal hypertrophic cardiomyopathy, hyperlactatemia, and pulmonary hypertension in early infancy. Front. Pediatr. *9*, 660076.
40. Wilfinger, N., Austin, S., Scheiber-Mojdehkar, B., Berger, W., Reipert, S., Praschberger, M., Paur, J., Trondl, R., Keppler, B.K., Zielinski, C.C., and Nowikovsky, K. (2016). Novel p53-dependent anticancer strategy by targeting iron signaling and BNIP3L-induced mitophagy. Oncotarget *7*, 1242–1261.
41. Acham-Roschitz, B., Plecko, B., Lindbichler, F., Bittner, R., Mache, C.J., Sperl, W., and Mayr, J.A. (2009). A novel mutation of the RRM2B gene in an infant with early fatal encephalomyopathy, central hypomyelination, and tubulopathy. Mol. Genet. Metab. *98*, 300–304.
42. Nowikovsky, K., Froschauer, E.M., Zsurka, G., Samaj, J., Reipert, S., Kolisek, M., Wiesenberger, G., and Schweyen, R.J. (2004). The LETM1/YOL027 gene family encodes a factor of the mitochondrial K+ homeostasis with a potential role in the Wolf-Hirschhorn syndrome. J. Biol. Chem. *279*, 30307–30315.
43. Gietz, R.D., and Schiestl, R.H. (2007). High-efficiency yeast transformation using the LiAc/SS carrier DNA/PEG method. Nat. Protoc. *2*, 31–34.
44. Zotova, L., Aleschko, M., Sponder, G., Baumgartner, R., Reipert, S., Prinz, M., Schweyen, R.J., and Nowikovsky, K. (2010). Novel components of an active mitochondrial K(+)/H(+) exchange. J. Biol. Chem. *285*, 14399–14414.
45. Bernardi, P. (1999). Mitochondrial transport of cations: channels, exchangers, and permeability transition. Physiol. Rev. *79*, 1127–1155.
46. Lee, H., and Yoon, Y. (2014). Transient contraction of mitochondria induces depolarization through the inner membrane dynamin OPA1 protein. J. Biol. Chem. *289*, 11862–11872.
47. Bauerschmitt, H., Mick, D.U., Deckers, M., Vollmer, C., Funes, S., Kehrein, K., Ott, M., Rehling, P., and Herrmann, J.M. (2010). Ribosome-binding proteins Mdm38 and Mba1 display overlapping functions for regulation of mitochondrial translation. Mol. Biol. Cell *21*, 1937–1944.
48. Mitchell, P., and Moyle, J. (1969). Translocation of some anions cations and acids in rat liver mitochondria. Eur. J. Biochem. *9*, 149–155.
49. Frazier, A.E., Taylor, R.D., Mick, D.U., Warscheid, B., Stoepel, N., Meyer, H.E., Ryan, M.T., Guiard, B., and Rehling, P. (2006). Mdm38 interacts with ribosomes and is a component of the mitochondrial protein export machinery. J. Cell Biol. *172*, 553–564.
50. Nowikovsky, K., Reipert, S., Devenish, R.J., and Schweyen, R.J. (2007). Mdm38 protein depletion causes loss of mitochondrial K+/H+ exchange activity, osmotic swelling and mitophagy. Cell Death Differ. *14*, 1647–1656.
51. Wortmann, S.B., Mayr, J.A., Nuoffer, J.M., Prokisch, H., and Sperl, W. (2017). A guideline for the diagnosis of pediatric mitochondrial disease: the value of muscle and skin biopsies in the genetics era. Neuropediatrics *48*, 309–314.
52. Niyazov, D.M., Kahler, S.G., and Frye, R.E. (2016). Primary mitochondrial disease and secondary mitochondrial dysfunction: importance of distinction for diagnosis and treatment. Mol. Syndromol., 122–137.
53. Burté, F., Carelli, V., Chinnery, P.F., and Yu-Wai-Man, P. (2015). Disturbed mitochondrial dynamics and neurodegenerative disorders. Nat. Rev. Neurol. *11*, 11–24.
54. Navaratnarajah, T., Anand, R., Reichert, A.S., and Distelmaier, F. (2021). The relevance of mitochondrial morphology for human disease. Int. J. Biochem. Cell Biol. *134*, 105951.
55. Nasca, A., Scotton, C., Zaharieva, I., Neri, M., Selvatici, R., Magnusson, O.T., Gal, A., Weaver, D., Rossi, R., Armaroli, A., et al. (2017). Recessive mutations in MSTO1 cause

mitochondrial dynamics impairment, leading to myopathy and ataxia. Hum. Mutat. *38*, 970–977.
56. Ryu, S.W., Jeong, H.J., Choi, M., Karbowski, M., and Choi, C. (2010). Optic atrophy 3 as a protein of the mitochondrial outer membrane induces mitochondrial fragmentation. Cell. Mol. Life Sci. *67*, 2839–2850.
57. Wortmann, S.B., Duran, M., Anikster, Y., Barth, P.G., Sperl, W., Zschocke, J., Morava, E., and Wevers, R.A. (2013). Inborn errors of metabolism with 3-methylglutaconic aciduria as discriminative feature: proper classification and nomenclature. J. Inherit. Metab. Dis. *36*, 923–928.
58. Zollino, M., Lecce, R., Fischetto, R., Murdolo, M., Faravelli, F., Selicorni, A., Buttè, C., Memo, L., Capovilla, G., and Neri, G. (2003). Mapping the Wolf-Hirschhorn syndrome phenotype outside the currently accepted WHS critical region and defining a new critical region, WHSCR-2. Am. J. Hum. Genet. *72*, 590–597.
59. Wilson, M.G., Towner, J.W., Coffin, G.S., Ebbin, A.J., Siris, E., and Brager, P. (1981). Genetic and clinical studies in 13 patients with the Wolf-Hirschhorn syndrome [del(4p)]. Hum. Genet. *59*, 297–307.
60. Yamamoto-Shimojima, K., Kouwaki, M., Kawashima, Y., Itomi, K., Momosaki, K., Ozasa, S., Okamoto, N., Yokochi, K., and Yamamoto, T. (2019). Natural histories of patients with Wolf-Hirschhorn syndrome derived from variable chromosomal abnormalities. Congenit. Anom. (Kyoto) *59*, 169–173.
61. Zollino, M., and Doronzio, P.N. (2018). Dissecting the Wolf-Hirschhorn syndrome phenotype: WHSC1 is a neurodevelopmental gene contributing to growth delay, intellectual disability, and to the facial dysmorphism. J. Hum. Genet. *63*, 859–861.
62. Van Buggenhout, G., Melotte, C., Dutta, B., Froyen, G., Van Hummelen, P., Marynen, P., Matthijs, G., de Ravel, T., Devriendt, K., Fryns, J.P., et al. (2004). Mild Wolf-Hirschhorn syndrome: micro-array CGH analysis of atypical 4p16.3 deletions enables refinement of the genotype-phenotype map. J. Med. Genet. *41*, 691–698.
63. Okamoto, N., Ohmachi, K., Shimada, S., Shimojima, K., and Yamamoto, T. (2013). 109 kb deletion of chromosome 4p16.3 in a patient with mild phenotype of Wolf-Hirschhorn syndrome. Am. J. Med. Genet. *161A*, 1465–1469.
64. Zollino, M., Orteschi, D., Ruiter, M., Pfundt, R., Steindl, K., Cafiero, C., Ricciardi, S., Contaldo, I., Chieffo, D., Ranalli, D., et al. (2014). Unusual 4p16.3 deletions suggest an additional chromosome region for the Wolf-Hirschhorn syndrome-Associated seizures disorder. Epilepsia *55*, 849–857.
65. Nakamura, S., Matsui, A., Akabane, S., Tamura, Y., Hatano, A., Miyano, Y., Omote, H., Kajikawa, M., Maenaka, K., Moriyama, Y., et al. (2020). The mitochondrial inner membrane protein LETM1 modulates cristae organization through its LETM domain. Commun. Biol. *3*, 99.
66. Maquat, L.E. (1995). When cells stop making sense: effects of nonsense codons on RNA metabolism in vertebrate cells. RNA *1*, 453–465.
67. Filograna, R., Mennuni, M., Alsina, D., and Larsson, N.G. (2021). Mitochondrial DNA copy number in human disease: the more the better? FEBS Lett. *595*, 976–1002.
68. Blomen, V.A., Májek, P., Jae, L.T., Bigenzahn, J.W., Nieuwenhuis, J., Staring, J., Sacco, R., van Diemen, F.R., Olk, N., Stukalov, A., et al. (2015). Gene essentiality and synthetic lethality in haploid human cells. Science *350*, 1092–1096.
69. Wang, T., Birsoy, K., Hughes, N.W., Krupczak, K.M., Post, Y., Wei, J.J., Lander, E.S., and Sabatini, D.M. (2015). Identification and characterization of essential genes in the human genome. Science *350*, 1096–1101.
70. Jiang, D., Zhao, L., and Clapham, D.E. (2009). Genome-wide RNAi screen identifies Letm1 as a mitochondrial Ca2+/H+ antiporter. Science *326*, 144–147.
71. Huang, E., Qu, D., Huang, T., Rizzi, N., Boonying, W., Krolak, D., Ciana, P., Woulfe, J., Klein, C., Slack, R.S., et al. (2017). PINK1-mediated phosphorylation of LETM1 regulates mitochondrial calcium transport and protects neurons against mitochondrial stress. Nat. Commun. *8*, 1399.
72. Austin, S., Tavakoli, M., Pfeiffer, C., Seifert, J., Mattarei, A., De Stefani, D., Zoratti, M., and Nowikovsky, K. (2017). LETM1-mediated K+ and Na+ homeostasis regulates mitochondrial Ca2+ efflux. Front. Physiol. *8*, 839.
73. Datta, S., and Jaiswal, M. (2021). Mitochondrial calcium at the synapse. Mitochondrion *59*, 135–153.
74. Doonan, P.J., Chandramoorthy, H.C., Hoffman, N.E., Zhang, X., Cárdenas, C., Shanmughapriya, S., Rajan, S., Vallem, S., Chen, X., Foskett, J.K., et al. (2014). LETM1-dependent mitochondrial Ca2+ flux modulates cellular bioenergetics and proliferation. FASEB J. *28*, 4936–4949.
75. Hashimi, H., McDonald, L., Stríbrná, E., and Lukeš, J. (2013). Trypanosome Letm1 protein is essential for mitochondrial potassium homeostasis. J. Biol. Chem. *288*, 26914–26925.
76. Natarajan, G.K., Mishra, J., Camara, A.K.S., and Kwok, W.M. (2021). LETM1: a single entity with diverse impact on mitochondrial metabolism and cellular signaling. Front. Physiol. *12*, 637852.

The American Journal of Human Genetics, Volume *109*

## Supplemental information

# Bi-allelic *LETM1* variants perturb mitochondrial ion homeostasis leading to a clinical spectrum with predominant nervous system involvement

**Rauan Kaiyrzhanov, Sami E.M. Mohammed, Reza Maroofian, Ralf A. Husain, Alessia Catania, Alessandra Torraco, Ahmad Alahmad, Marina Dutra-Clarke, Sabine Grønborg, Annapurna Sudarsanam, Julie Vogt, Filippo Arrigoni, Julia Baptista, Shahzad Haider, René G. Feichtinger, Paolo Bernardi, Alessandra Zulian, Mirjana Gusic, Stephanie Efthymiou, Renkui Bai, Farah Bibi, Alejandro Horga, Julian A. Martinez-Agosto, Amanda Lam, Andreea Manole, Diego-Perez Rodriguez, Romina Durigon, Angela Pyle, Buthaina Albash, Carlo Dionisi-Vici, David Murphy, Diego Martinelli, Enrico Bugiardini, Katrina Allis, Costanza Lamperti, Siegfried Reipert, Lotte Risom, Lucia Laugwitz, Michela Di Nottia, Robert McFarland, Laura Vilarinho, Michael Hanna, Holger Prokisch, Johannes A. Mayr, Enrico Silvio Bertini, Daniele Ghezzi, Elsebet Østergaard, Saskia B. Wortmann, Rosalba Carrozzo, Tobias B. Haack, Robert W. Taylor, Antonella Spinazzola, Karin Nowikovsky, and Henry Houlden**

**Supplemental data**

**Supplemental note: case reports**

***Family 1***

This family presents with two affected siblings. The index case (F1:S1) is a 35-year-old female born full-term following uneventful pregnancy and delivery to non-consanguineous healthy British parents.Her neonatal period was unremarkable with normal birth weight, height, and occipitofrontal circumference. The parents have started expressing concerns from about one year of age. She has never crawled and always been unsteady when standing. She has never acquired speech and always had difficulty swallowing. She walked at around 17 months of age, and although she was delayed in walking, she did learn to run and did ballet until she was around the age of 6 or 7 years. From that time, there seemed to be a deterioration in her balance overall, although she did fluctuate from time to time. Treatment with acetazolamide did seem to help with less period of fluctuation. Continuous deterioration in ataxia started from age 7 years and had led to the loss of independent ambulation by age 13 years. Hearing loss was confirmed at the age of 2 years but in retrospect, her parents feel that she probably never heard. Visual problems were noted at around the age of 5 years, and she is now registered with partially sighted. Learning difficulties became apparent when she started at a school with a unit for hearing impairment and around the same age, intellectual disability was diagnosed. At 14 years old generalized tonic-clonic seizures started with the maximum frequency of one per two years. Seizures were well-controlled by carbamazepine. At age 18 years, she developed insulin-dependent diabetes mellitus. No behavioral symptoms and autism were reported. She did not develop cardiomyopathy and her electrocardiography together with echocardiography were normal. Her disease course has been slowly progressive.

With suspicion of mitochondrial disease as a cause of her symptoms, she underwent various investigations. Visual evoked potentials showed post retinal dysfunction and electroretinogram was normal. Nerve conduction studies showed signs of axonal sensory-motor neuropathy and electromyography was revealing for denervation and re-innervation processes. Muscle biopsy, white cell enzymes, very-long-chain fatty acids, and CSF lactate were normal. Mitochondrial respiratory chain enzymes showed borderline-low activity of complex II+III and IV. Co-Q levels were slightly low (127, normal value 140-580) and alanine was slightly raised at 38 in CSF. Genetic tests for *SCA1, SCA2, SCA3, SCA6, SCA7, FRDA*, EA2, common *POLG* mutations, mtDNA rearrangements, common point mutations of mtDNA were negative. Plasma amino acids analysis showed increased alanine, glycine, and serine.

Upon recent examination at age 35 years, she had short stature (height 153 cm) and a small head circumference (50 cm). Her face was dysmorphic including a long thin face with a prominent nose, low sitting ears, and teeth abnormalities. Joint contractures at knees, elbows, and hands together with kyphoscoliosis were present. She was alert but non-verbal and could make only unintelligible sounds. The cranial nerves examination revealed bilateral optic atrophy, mild weakness of facial muscles, and severe bilateral sensorineural deafness. Her arms and legs were slim, and her feet and hands were cold with wasted muscles. In combination with reduced pinprick sensation, these findings suggested peripheral neuropathy. There was significant spasticity in the upper and lower limbs. She could make several steps with support and her gait was spastic-ataxic. Myoclonic jerks in limbs and facial muscles were observed. Muscle jerks precipitated by movement. Her movements generally were slow. A tremor in fingers when arms outstretched was also noticeable. Deep tendon reflexes were brisk at the knees and ankles with feet clonus and upgoing plantars bilaterally. Biceps tendon reflexes were present on the right side and reduced on the left. Supinator reflexes were also reduced bilaterally. Brain MRI showed severe pontine and cerebellar atrophy.

Affected brother (F1:S2)

This is a 25-year-old male born full-term following a pregnancy complicated by one kidney infection in his mother. He was born by normal vaginal delivery and his birth weight and length were normal. There were no concerns initially but, again, he was a late walker at 17 months. He did however crawl at around 9 months of age and did learn a few words. His parents feel that he was always different from his sister. Brain stem evoked potentials at six weeks of age were reported to be normal. His parents had concerns regarding his balance and felt that when he did learn to walk it was “like a little old man”. When it became apparent that he was unstable on his feet, further investigations confirmed that he had a bilateral hearing loss at age 18 months. He has been wearing hearing aids since age 5 years. An audiogram at 5 years old reported a profound high-frequency loss and a moderate-to-severe low-frequency loss. He had attended a school with a hearing unite there had been some improvement in speech. By age 5 years he was found to have impaired vision. The disease has had a slowly progressive course and he lost autonomous ambulation by age 6-7 years. Nevertheless, his symptoms have always been less severe than that of his sister. From the age of 9 years, he developed generalized tonic-clonic seizures that used to come in clusters 2-3 times every 2-3 months. The duration of seizures was around 2-3 minutes and they were well-controlled by antiepileptic medication. He did not express cardiac complications but recently diabetes was diagnosed. Muscle biopsy, EMG, NCS conducted at

age 5-6 were reported to be normal. Plasma amino acids analysis showed Increased serine, glycine, and homocysteine. Plasma FGF21 levels were within normal limits (740pg/ml).

Upon examination at age 25 years, he presented with an almost similar phenotype as his sister but at the milder end of the spectrum. He had a short stature with a small head circumference (height 152 cm, head circumference 52 cm). Mild dysmorphic facial features and joint contractures at knees, elbows, and hands were also present. He was intellectual disability, right sided esotropia, and bilaterally pale optic discs. He could say some words with dysarthric speech and some of his words were comprehensible. The rest of the neurological examination was similar to his sister's findings.

***Family 2***

The index case (F2:S1) is a 24-year-old man born full-term after uneventful pregnancy and delivery to consanguineous healthy parents of Pakistani origin. He has two unaffected sisters. His birth weight was 3.35 kg and no neonatal abnormalities were found. Normal development was reported until age 2½ years when he gradually lost language skills. After initial normal motor development, his motor function has regressed from 4 years of age along with his cognition. His disease slowly progressed and by age 5 years he manifested epileptic seizures that have been well controlled. The type of seizures included focal, generalized, and episodes of absences. Their frequency was 1-3 per year over the recent years, and they typically last for one minute. EEG at 18 years showed 3-4 Hz activity in the left fronto-pretemporal region as single potentials and short trains, with amplitude up to 150 microvolts, and a single 3-4 Hz irregular spike/wave paroxysm lasting 1 second. At age 6 years he was diagnosed with bilateral sensorineural deafness and hearing aids were fitted. He has gradually developed severe spasticity requiring bilateral Achilles' tendon surgery (extension) at age 12 years and 18 years. There were no signs of cardiomyopathy, and an ECG was normal at age 20 years. At age 15, his weight was 33 kg and his height was 170 cm.

As an adult, he has severe psychomotor retardation, optic atrophy (6/38 Cardiff at age 21 years) with nystagmus, bilateral cataracts combined with spasticity, hand tremor, and atrophy of small hand muscles. He does not speak and uses a wheelchair. He did not have dysmorphic features, scoliosis, diabetes, or behavioral problems. No cerebellar ataxia, hypotonia, myopathy, or neuropathy were reported. Screening of urine showed excretion of methylglutaconic acid. Brain MRI at age 4 was reported to be normal.

***Family 3***

This family had 3 affected siblings born to consanguineous Kuwaiti parents.

Index case (F3:S1)

The index case was a female born at term with a birth weight of 2.9 kg and her mother had gestational diabetes. Her Apgar scores were 8-1 and 9-5 and she was admitted to SCBU for overall assessment due to a family history of previous infant deaths. Neonatal echocardiography revealed mild ventricular hypertrophy, a small atrial septal defect (ASD), and a patent foramen ovale (PFO). Serum lactate (3.56 mmol/L) and CK-MB (9.7 ng/ml) were elevated. Repeat echocardiography showed 2 small ASDs, left ventricle hypertrophy that evolved to biventricular hypertrophy with a mild left ventricular outflow tract obstruction (LVOTO). At 2 months old, her blood tests showed normal readings for complete blood count, amino acids, ions, metabolites, carnitine, and acylcarnitine, while her serum lactate level was reduced (2.56mmol/L). At 4 months old, she was noted to have sweats during feeds. Her length and head circumference were above the 10th centile, she had nystagmus, blue sclera, and abnormal hearing but no ptosis was observed. Significant shoulder girdle and limb hypotonia were noted with no brisk deep tendon reflexes (DTRs). At 7 months old, echocardiography revealed pericardial effusion for which she was hospitalized. Her weight was on the 3rd centile and her head circumference and height were on the 10th centile. Hypotonia persisted but brisk DTRs were noted. At 9 months old, an MRI brain scan showed no significant findings other than a mild ventriculomegaly. At 1 year old, her mother noticed a reduced amount of urine and she became lethargic, drowsy, pale, with poor feeding, which later progressed to cyanosis and tachypnoea. She had tachycardia (210 beats per minute) with no measurable blood pressure for which she was put on a mechanical ventilator. At the same time, she was treated for metabolic acidosis. Echocardiography showed a hypertrophic dilated left ventricle with poor systolic function. She passed away 2 days later due to refractory cardiogenic shock and disseminated intravascular coagulation.

Affected older brother (F3:S2)

The proband's older affected brother was born at term with no neonatal problems. At 6 months old, his parents noticed him becoming floppy with difficulty feeding and failure to thrive. A gastrostomy was performed, and a feeding tube was fitted. By 7 months old, his development stopped, and he presented with hearing problems and nystagmus. Dysmorphic features and facies myopathica were noted along with reduced visual acuity. Lactate levels were repeatedly elevated, EMG reported myopathic changes, and a brain MRI scan showed non-specific changes. Muscle biopsy analyses reported histopathological changes associated with mitochondrial disorders, electron microscopy

reported normal mitochondrial structure, and respiratory chain enzyme activity was reduced in OXPHOS complexes I, II, III and IV. mtDNA depletion was suspected but DNA quantification analysis reported no mtDNA depletion.

He later presented with cyanosis, respiratory distress, tachycardia, and desaturation, which was suggestive of pneumonia, and was connected to a mechanical ventilator. Arterial blood gases showed respiratory acidosis with elevated serum lactate at 12.65 mmol/L. He had non-generalized edema with cold extremities, distended abdomen with abdominal wall edema. He was tested and was diagnosed with septic shock due to a Pseudomonas infection with CMV infection. This resulted in multiorgan failure including liver and renal failure. He became hypotensive and echocardiography showed left ventricular hypertrophy and ejection fraction was 50%. He became bradycardic and was resuscitated numerous times until he passed away soon after.

Affected older brother (F3:S3)

The proband's younger affected brother was a product of 36 weeks gestation with a birth weight of 2.7 kg. His Apgar scores were 7-1 and 8-5. His mother had gestational diabetes and was only placed on a diet. He was admitted to the Special care baby unit due to tachypnoea, grunting, and missed heartbeats and so was at the hospital for the first 2 weeks of his life. A systolic murmur was audible, but the heart ultrasound showed a thick myocardium with a first-degree heart block. Echocardiography showed hypertrophied left ventricle and repeat echocardiography showed the same presentation with good ventricular function. Repeat lactate level readings showed a sharp elevation (11 mmol/L) that gradually normalized (2.4 mmol/L). An elevation in pyruvate levels was also measured. At 50 days of life, he had a 2-day history of cough, poor feeding, vomiting, and inactivity. Lactate was elevated (4.1 mmol/L) but reduced upon repeating the read. At 4 months old, he developed respiratory distress, fever, a runny nose, and a cough. A throat swab identified a Candida infection. Lactate levels were elevated (3.57 mmol/L) and he was treated with IV fluids, nebulized salbutamol, ipratropium bromide, cefotaxime, and erythromycin. He was later noted to be developmentally delayed with his head circumference, length, and weight all measuring below the 3rd centile. He was alert with a pale complexion and had an extended posture in both his lower limbs. He was hypotonic with normal DTRs. At 5 months old, he presented with tachypnoea and cardiomegaly due to pericardial effusion identified by echocardiography. The effusion resolved after he was put on protein-based powder milk. A metabolic blood workup showed elevated creatine phosphokinase (251 U/L), elevated lactate (6.54 mmol/L), elevated lactate/pyruvate ratio (105.6), and elevated total carnitine (105 μmol/L). A follow-up test 2 weeks later showed reduced lactate levels (2.66 mmol/L)

with an elevated lactate/pyruvate ratio (111), elevated alkaline transferase (75 U/L), elevated CK-MB (10.5 µg/L), and elevated CPK (82 U/L).

At the age of 1 year, he developed progressive respiratory distress and was admitted to a hospital. Arterial blood gases showed metabolic acidosis and echocardiography showed a pericardial effusion which was resolved by pericardiocentesis. He again developed respiratory distress and metabolic acidosis and was intubated and managed for 3 days before being shifted to pressure-controlled ventilation mechanical ventilators for further management. His lactate level was 6.4 mmol/L and his chest X-ray showed bilateral haziness and cardiomegaly. Tracheal aspirate showed growth of Pseudomonas aeruginosa and Klebsiella pneumonia. A repeat tracheal aspirate 2 weeks later showed the presence of the Pseudomonas aeurginosa and Klebsiella pneumonia. ECG showed sinus bradycardia and he required a blood transfusion. On discharge, his lactate level was 2.9 mmol/L. The younger brother passed away at the age of 1 year and 3 months with no report on the cause of death.

***Family 4***

The index case (F4:S1) is an Egyptian boy born full-term to consanguineous first cousin parents after a pregnancy complicated with hypertension. His birth weight was 2.7 kg and the neonatal period was unremarkable. He manifested at 4 months of age with nystagmus, hypotonia, and myopia. His disease had moderate rates of progression. He sat autonomously at 9 months of age, started walking independently at 20 months, and his first words started at the age of 16 months. The onset of progressive bilateral deafness was noticed from age 18 months and hearing aids were fitted from age 22 months. At 18 months of age, he had one attack of febrile convulsions and it was repeated only once at age 8 years. By the age of 2.5 years, he was noticed to lose independent ambulation due. Around the age of 3 years old, he developed significant impairment of vision and bilateral cataracts. First echocardiography done at age of 3 years showed mild ventricular hypertrophy and minimal mid cavity obstruction with pericardial effusion. There was one episode of haematemesis. He used to be admitted to hospitals due to hypoglycemia and metabolic acidosis. On examination at age 3 years, he presented with myopia, nystagmus, bilateral sensorineural hearing loss, and had midfacial hypoplasia. The speech was delayed as he used only sounds. He was hypotonic with myopathy. He was not reported to have ataxia spasticity, or neuropathy, feeding difficulties, muscular atrophy, encephalopathy, and hyperreflexia were present. Intellectual disability, behavioral abnormalities, and autism were not documented as well. He did not have kyphoscoliosis and no signs of retinitis pigmentosa were found. A brain MRI scan showed a mild reduction in brain volume. Lactic acidosis was reported but only with attacks of hypoglycemia. His renal function tests, plasma, and urine amino

acids were found to be normal. There was a significant elevation of adipic acid in the urine organic acids test. He died at age 8 years and the cause of death was unavailable.

***Family 5***

The proband (F5:S1) is an 11-year-old male, born by cesarean section to healthy non-consanguineous parents from Chechnya after an uneventful twin pregnancy with a birth weight of 2.4 kg. At the age of about 7 months, unilateral nystagmus of the left eye was noticed, at about 12 months also of the right eye. Eventually, atrophy of the optic nerve was diagnosed with pronounced visual impairment. A hearing loss was noticed since the 5th year of life. No other significant developmental problems were noted in early childhood. He was a poor eater and noted to be nervous and at times aggressive.At first presentation with 8 years, he showed a failure to thrive (BMI 12.7; -2.5 z) and slight microcephaly (-2.5 z), furthermore hirsutism, nystagmus, visual and hearing impairment, reduced muscle mass, externally rotated feet, and motor coordination deficits. Brain MRI showed optic nerve and chiasm atrophy. At the age of 9 years, he was admitted to a local hospital because of exercise intolerance which had been noticed at running, cycling, and swimming, maximum walking distance of1 km. A treadmill ergometry (600 m, 6 km/h, slope 10 %) led to a heart rate increase from 105 to 180/min, breathing rate 58/min, drop of pH from 7.44 to 7.06, lactate increase from 3.1 to 20 mmol/l.Other metabolic laboratory investigations and cardiology examinations were unremarkable. A thorough investigation revealed the following results: electroencephalography with background slowing, learning disability, mildly increased lactate and alanine in blood and CSF, known visual and hearing impairment, no further organ involvement. A muscle biopsy was unremarkable histologically.Western blot analysis of mitochondrial markers in muscle showed a global reduction for complexes I-IV, whereas complex V was slightly increased. Measurement of respiratory chain enzymes displayed a combined defect of complexes I, III, and IV in muscle. Medication with ubiquinone was started. At 10 years of age, a routine laboratory control showed lactate 5.1 mmol/l, pH 7.32, and glucose 8.0 mmol/l. An increased HbA1C level and pathological oral glucose tolerance test led to the diagnosis of type 3 diabetes and eventually treatment with repaglinide was started. At the current age of 11 years, the patient is underweight and slightly microcephalic, has visual and hearing impairment, a mild intellectual disability, muscular atrophy, otherwise no further neurological issues. He uses glasses, a reading device, and hearing aids and attends a special needs school. His FGF21 was 861 pg/ml (normal value <200).

He has three healthy siblings including his twin brother. Another 6-year-old sister has a history of unexplained acute symptoms in early infancy, according to the parents she had to be resuscitated. In

the further course febrile convulsions, night spasms, reduced muscle mass, externally rotated feet, rectal bleeding leading to diagnosis and removal of tubular adenoma of the rectum, hirsutism, mild hepatomegaly, and iron deficiency anemia occurred. Currently, there is no obvious developmental delay. Blood gases, lactate, and alanine were unremarkable. Sanger sequencing confirmed the familiar LETM1 variant in a heterozygous state. The family history is otherwise unremarkable.

***Family 6***

The index case (F6:S1) is a 17 month old female born to non-consanguineous Mexican parents. She has one male sibling with a possible autism spectrum disorder. Her prenatal period was remarkable with maternal polyhydramnios, 3rd-trimester spotting, and cervical incompetence. She was born full-term with an occipitofrontal circumference of 36 cm, birth weight of 3.1kg, and birth length of 48cm with Apgar scores of 8 and 9. Her neonatal period was significant for apnea, poor feeding requiring G-tube, laryngomalacia, and two ventricular septal defects, one of which was spontaneously closed later. The disease manifested from birth with poor feeding. She had acquired motor milestones at a normal age: sat at 6 months old, independent walking started at the age of 16 months, and the first words have started from the age of 8 months. Although, some delay in intellectual functioning was reported. Her feeding difficulties have gradually resolved, and the disease course was reported to be static. Upon her examination at the age of 2 months, her head circumference was below 3rd centile, and shehad bitemporal narrowing, micrognathia, high arched palate, overriding posterior sutures, and Anterior Fontanelle Open and Flat. Her weight and height were above the 50th percentile. She had normal vision and hearing with no nystagmus, ptosis, or ophthalmoparesis. On neuromuscular examination, she displayed normal muscle tone with no signs of muscular atrophy or hyperkinetic movement disorders. She did not display clinical seizures, but her EEG was abnormal due to excessive sharp transients with no epileptiform activity. No signs of diabetes and kyphoscoliosis were found upon her examination at 2 months old. From laboratory investigations only plasma amino acids wereavailable with normal results. Brain MRI conducted at age 1 week showed mild vermian hypoplasia.

***Family 7***

This family presents with 2 affected male siblings (F7:S1 and F7:S2) both born full-term after unremarkable pregnancies to healthy consanguineous Pakistani parents. The older affected sibling is currently aged 15 years old. The disease manifested at 1.5 years old with muscular weakness and difficulty standing up. He had a mild developmental delay with the acquisition of gait and first words

by the age of 2 years. At this time parents noticed an unsteady gait. His disease has had moderate rates of progression leading to the loss of ambulation by the age of 5 years and regression of speech. From the age of 5 years, he has started experiencing one per day generalized tonic-clonic seizures. The seizures were well controlled. By 11 years old he displayed impaired vision. He has shown no signsof metabolic acidosis or respiratory distress. Upon examination, at age 15 years his was significantly underweight (below 3rd centile) with small head circumference (below 3rd centile), kyphoscoliosis, and no clear features of dysmorphism. He is non-verbal with intellectual disability, communication, and behavioral issues. On neurological examination, he had appendicular spasticity with brisk tendon reflexes, upgoing plantars, and muscle atrophy. Examination of the sensory organs was remarkable only for impaired vision with normal hearing. He had chronic constipation and sleep disturbances, which was one of the main medical issues reported by the parents. His liver and renal function tests were normal and neurophysiology studies showed mil neuropathic changes.

The phenotype of the younger affected brother, currently aged 8 years, is similar to the older brother. He manifested at age 2 years with difficulty standing and walking with consequent ataxic gait and later motor regression with loss of ambulation by age 5 years. His brain MRI showed T2WI/FLAIR T2WS/FLAIR hyperintensities in periventricular white matter of bilateral parietooccipital lobes on both sides. Severe optic nerve and chiasm atrophy.

***Family 8***

The index subject (F8:S1) is a 37-year-old Italian patient born from first-degree cousins. She presented at birth with dysmorphic features, namely micrognathia and low set ears, divergent strabismus, and bilateral ptosis. She acquired normal gait at 10 months of age; early during childhood she manifested with growth and psychomotor delay, speech disturbances associated with severe sensorineural hypoacusis and bilateral cataracts. She also presented progressive gait ataxia and limb incoordination, diffuse skeletal muscle hypotrophy and facial hyposthenia. She was diagnosed with combined pituitary hormone deficiency during later development to early menopause and consequently leadingto severe osteoporosis. First brain MRI at the age of 8 demonstrated cerebral cortical atrophy, predominantly vermian cerebellar hypoplasia, abnormal optic tracts small pituitary gland. Severe bilateral brainstem dysfunction was evident at auditory evoked potentials, while electromyography displayed myopathic features without neuropathy. A muscle biopsy was performed when she was 11years old. Histology and histoenzymatic analysis were consistent with neurogenic muscle atrophy and mitochondrial dysfunction as several ragged-red fibers and COX-negative fibers. Biochemistry revealed reduced activity of mitochondrial respiratory complexes apart from complex II.

Last examination available, at 37 years of age, displayed a severely worsened ataxic gait, dysarthria, incoordination, oculomotor abnormalities and nystagmus; low body mass index (BMI), associated with advanced generalized amyotrophy and reduced deep tendon reflexes was also documented. A complete ophthalmologic evaluation was remarkable with tight bilateral myosis and disclosed an eso-hypertropia and signs of bullous keratopathy. Basic neuropsychological tests revealed behavioural disturbances with emotional lability and panic attacks reported by parents, marked intellectual disability with executive dysfunctions and defective verbal denomination. Repeated panic attacks were also reported by parents. Her plasma FGF21 levels were significantly elevated 2554 (normal value 0 - 153 pg/ml). The gas-chromatographic plot, relative to the urine sample, showed an increasein the excretion of some intermediates of the cycle of tricarboxylic acids (succinic, fumaric, 2-ketoglutaric and aconitic) associated with an increase in acid excretion 3-methylglutaconic and 3- methylglutaric. In addition, an increased excretion of pyruvic acid was found together with a modest increase in 3-hydroxybutyric acid (both in the -iso and in the -n forms). Urine amino acid analysis revealed increased levels of aspartic, serine, and glycine.

Her father developed ataxic wide-based gait during his 50s with evidence of mild dysarthria, limb incoordination and mild dysdiadochokinesia and bradykinesia during a follow up period of 9 years. Anamnestic recollection revealed a history of anxiety, panic attacks and phobic behaviour treated with serotonin reuptake inhibitors and benzodiazepines. Last clinical examination was performed when he was 79 years old: gait had worsened over time requiring the use of a walking aid for short-medium distance walking; neurological examination also showed moderate limb apraxia and signs of mild neuropathy, with absent tendon reflexes and impaired vibratory sensation on lower limbs Defective attentive and executive functions and weakened visual memory were documented by cognitive tests. Muscle strength was normal. Brain MRI displayed a predominantly vermian moderate cerebellar atrophy and mild brain atrophy.

The mother and the older sister of the proband were healthy; no health problems in the paternal grandparents were reported.

***Family 9***

The affected child (F9:S1) is a 1 year and 2 months old girl born full-term after uneventful pregnancy to consanguineous Pakistani parents. She was noted to have a weak cry, stridulous breathing, and recurrent apneas starting a few hours after birth with an initially normal neurological examination and Apgar scores of 9-9-9. Starting from the first days after birth and progressing over the first 4 months

of life, she developed progressive central and peripheral hypotonia with a paucity of spontaneous movements with poor respiratory effort. She spent ~ 4 months in PICU with ventilator support. She failed extubations on multiple occasions and finally had a tracheostomy at ~ 3 months of life with long-term ventilatory support. She had a cardiac arrest secondary to an apneic episode with downtime of 4 min during a brief period of trial of extubation at ~ 2 months of age. Subsequent MRI did not show any hypoxic-ischemic element. She failed the newborn hearing test; subsequent repeat hearing tests were technically difficult as she was ventilated in PICU. These were suggestive of sensorineural hearing loss. There was mild left ventricular hypertension felt secondary to hypertension but no cardiac dysfunction. Refractory infantile spasms and myoclonic jerks started at around 7 months of age. There were hourly clusters of spasms at peak. Spasms reduced and did not completely settle with adrenocorticotropic hormone. EEG did not show any improvement with steroid therapy and continues to be abnormal in keeping with epileptic encephalopathy. The EEG conducted at 4 months old shows evidence of mild maturational lag expressed by an asymmetrical increase in posterior slow. The repeat EEG at 5 months old has significantly deteriorated compared to the previous recording. The occasional sharp transients seen previously are now replaced by frequent bilateral and independent epileptiform discharges. She was initially bottle-fed but later nasogastric tube-fed after the need for ventilator support. Currently, she has poor suck and swallows. The main medical problems reported by parents were hypotonia, poor respiratory effort, and epileptic encephalopathy.

At her last follow-up examination at age 1-year-old, she had a global developmental delay with some responsiveness to surroundings- touch, sounds, and visual stimuli. There was a full range of ocular movements with perioral myokymia and intermittent nystagmus. She had a normal ophthalmologic assessment with no evidence for optic atrophy. However, she did not reliably fix or follow objects. There was peripheral and central hypotonia with mild flexion contracture at wrists and knees. There were no clinical or EMG signs of peripheral neuropathy.

Regarding investigations, basic metabolic workup on serum and urine was unrevealing. She had transient mildly elevated CSF lactate with normal plasma lactate. Abdominal ultrasound showed a slightly enlarged liver with echogenic parenchyma and normal kidneys. There was borderline reduced complex IV and muscle biopsy was suggestive of neurogenic but also had quite a lot of glycogen. Brain MRI scans from days 11 and 15 of life were reported to be normal. CGH microarray, tests for congenital myasthenic syndrome, common mitochondrial mutations, and SMA gene were negative.

***Family 10***

This family presents with currently deceased 2 affected siblings (F10:S2 and F10:S3) and affected maternal aunt (F10:S1) all born to consanguineous Portuguese parents. Due to early infantile death, their clinical details were limited. Their available medical records reported early onset rapidly progressive disease with hypotonia, spasticity, seizures present in all affected, and additional deafness, elements of cerebellar ataxia, and poor feeding present in the affected aunt. The three cases also had raised urinary 3-MGA (F10:S1 268, F10:S2 “detectable, not quantified” and F10:S3 1363 mol/mmol creatinine, reference range < 20). Both the affected aunt and the younger sibling had Complex I deficiency in muscle (37 and 38%, respectively).

***Family 11***

This family had 2 affected siblings (F11:S1, F11:S2). Subject 1 (female) was the first child of non-consanguineous healthy parents (Figure 2). She was born at 40 weeks from spontaneous delivery after an uneventful pregnancy. She presented at birth with cataracts and muscular hypotonia and was admitted to the Bambino Gesù Children’s Hospital at the age of 8 months for apneic spells and cyanosis during crying. Clinical examination at admission documented severe muscular hypotonia, nystagmus and convergent strabismus, bilateral cataracts and retinal hyperpigmentation, systolic murmur, hepatomegaly with lower liver margin at 2 cm from the costal arch. Biochemical investigations documented metabolic acidosis, hyperlactacidemia (6.5 µmol/L, nv<2.1), low levels of free carnitine (15 µmol/L; normal value >26) and increased esterified/free carnitine ratio (3; normal value <0.3), hypertransaminasemia (GOT/GPT 88/135 UI/L; normal value <40), with normal blood glucose, LDH and CPK levels. Urinary organic acids profile showed increased excretion of Krebs cycle metabolites. EKG and echocardiogram documented the presence of hypertrophic cardiomyopathy. Brain CT scan and MRI were negative. Severe sensorineural deafness was detected by brainstem auditory evoked potentials and audiometry. Visual evoked potentials documented an increased latency. Electroretinogram was severely abnormal for increased latency and reduced amplitude. The electrophysiological study of the peripheral nervous system disclosed an axonal-type motor-sensory polyneuropathy. Muscular biopsy documented the presence of a type I fiber predominance, with mild glycogen storage. No typical RRFs were detected, but histochemical staining for SDH showed a widespread markedly increase signal due to mitochondrial proliferation, more evident in some fibers, while reaction for Cytochrome C oxidase (COX). Biochemical assays of MRC activities documented a defect of COX (10.92 nmol/min/mg protein; nv:17.87+/-3.99) and NADH cytochrome c reductase (796.74 nmol/min/mg protein; 1396.91+/-376.87). Molecular genetic studies performed on mtDNA extracted from muscle tissue ruled out mtDNA deletions. The patient was dismissed at home under

carnitine therapy and with external acoustic prostheses. Two additional brain MRIs performed during follow up at 13 and 31 months documented brainstem and cerebellar vermis hypoplasia, ventriculomegaly and delayed myelination. At the last examination, at 5 years, the patient showed severe moderate psychomotor delay with visual defect and bilateral nystagmus. The child died at 6 years of heart failure.

Subject 2 (male), the younger brother of subject 1, was the fourth child of the couple. He was born at42 weeks from spontaneous delivery after a pregnancy complicated by oligohydramnios. Weight at birth was 2.95kg (<3rdpercentile). He presented at birth with hypotonia and respiratory distress and was treated with oxygen therapy. At age 6 days a megacolon was suspected. Rectal biopsy however was negative. He also suffered from sepsis, requiring antibiotics, and necrotic enterocolitis. He was then transferred to Bambino Gesù Children's Hospital at the age of 2 months. Clinical examination atthe admission documented muscular hypotonia and severe failure to thrive. Routine biochemical testsshowed metabolic acidosis (bicarbonates 17 meq/l, base excess -10), responsive to oral NAHCO3 supplementation, hyperlactacidemia (up to 9.6 µmol/l, normal value <2.1), mild increase of transaminases (GPT/GOT 162/139 UI/L.; normal value <40), with normal glucose, ALP, LDH and CPK levels. Metabolic investigations revealed mild generalized increase of plasma aminoacids and significant excretion of lactic and pyruvic acids, Krebs cycle metabolites (2-ketoglutaric, 2-etil-3- hydroxypropionic, and fumaric acid) and tiglylglycine at urinary organic acids profile. EKG was normal,but an echocardiogram documented the presence of mild left ventricular hypertrophic cardiomyopathy. Based on the clinical phenotype and the family history, a mitochondrial disease wassuspected, and skin and muscle biopsies were performed at 3.5 months. Despite bicarbonate therapy,his clinical conditions and acidosis progressively worsened, and he died at 4.5 months of age of heartfailure.

Sanger sequencing in the following genes *SCO1, SCO2, COX15, EFG1, EFG2, EFG3, SUCLA2; MTO1*, *DNAJC1,* did not display any mutations. Moreover, based on the clinical association of cataracts, mitochondrial myopathy and cardiomyopathy, Sengers syndrome was hypothesized, however, sequencing of coding regions of AGK revealed no pathogenic changes. The possibility of MEDGEL syndrome was excluded as well by normal molecular testing of SERAC1 gene. A targeted resequencing for 1381 genes encoding for mitochondrial proteins ("Mitoexome") was performed.

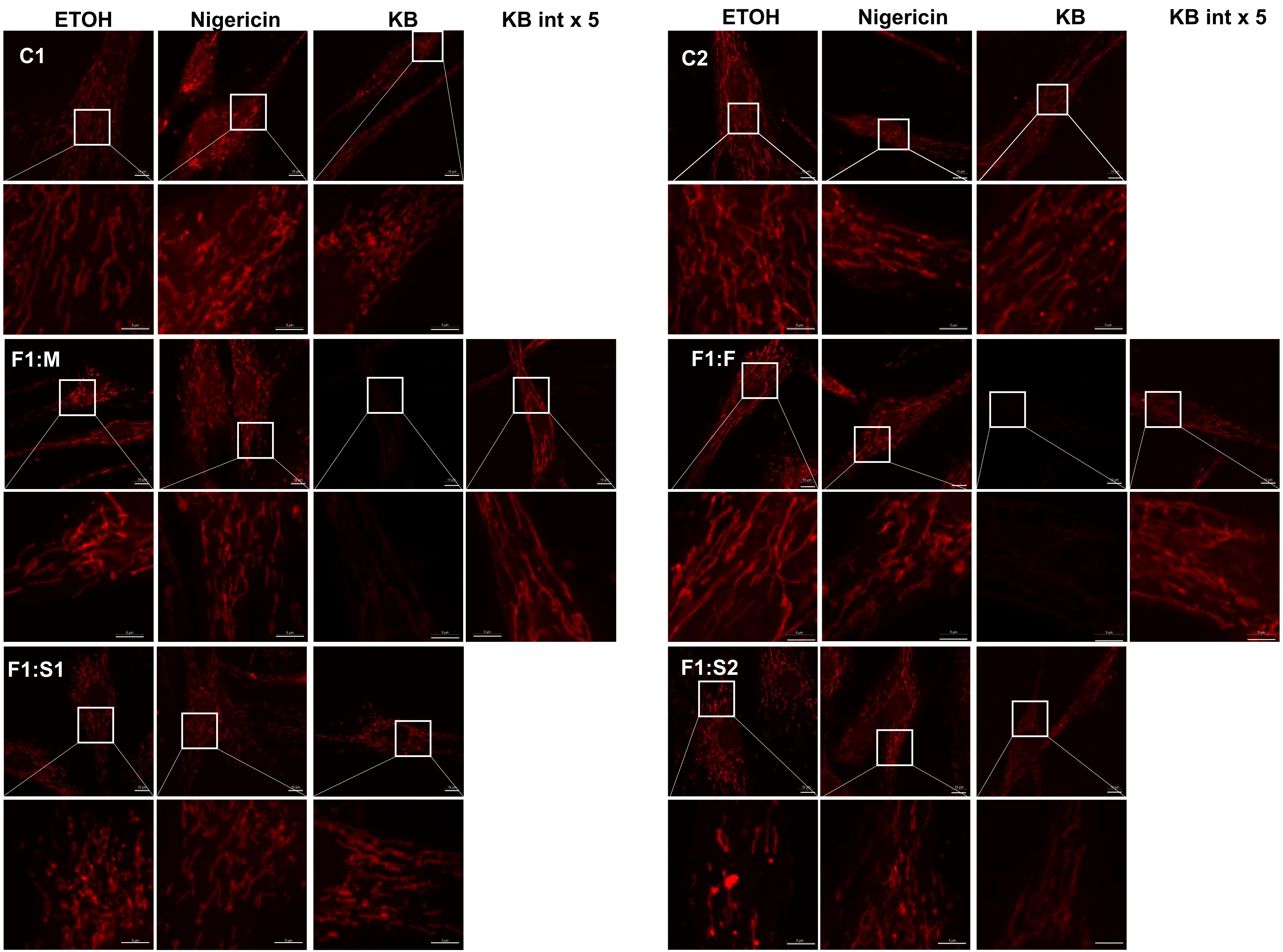


Figure S1A

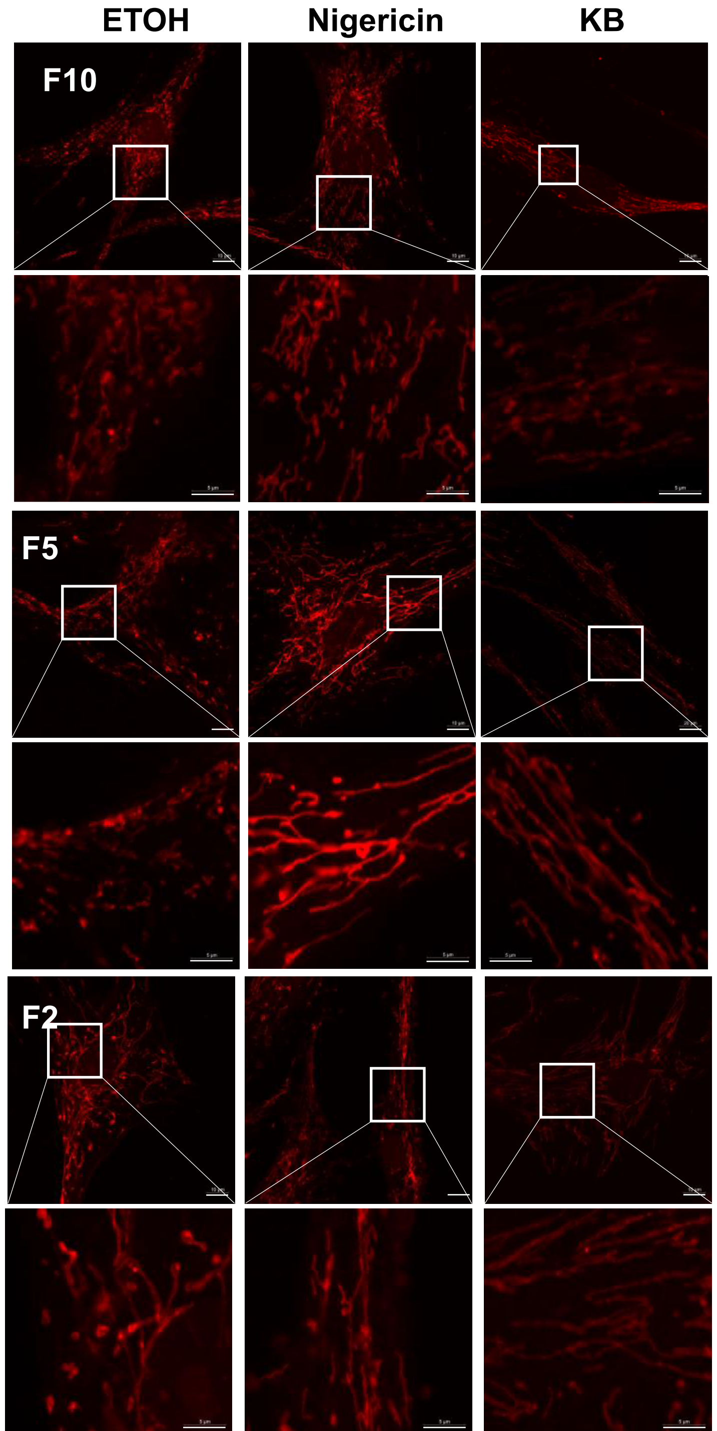


**Figure S1A**

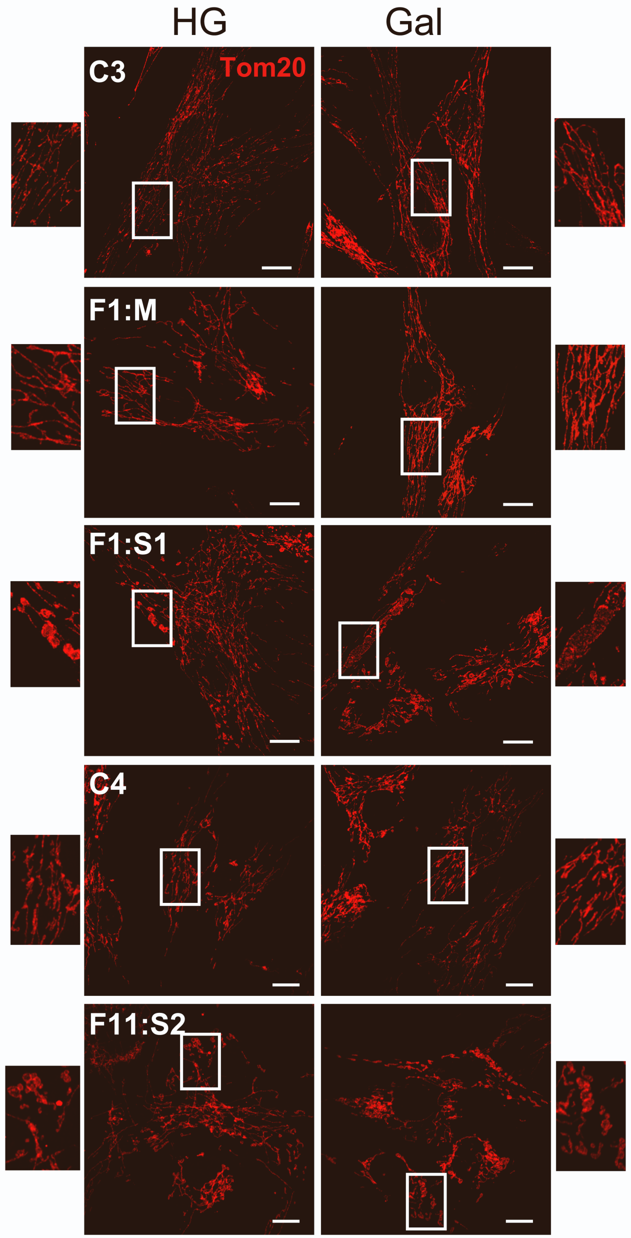


**Figure S1B**

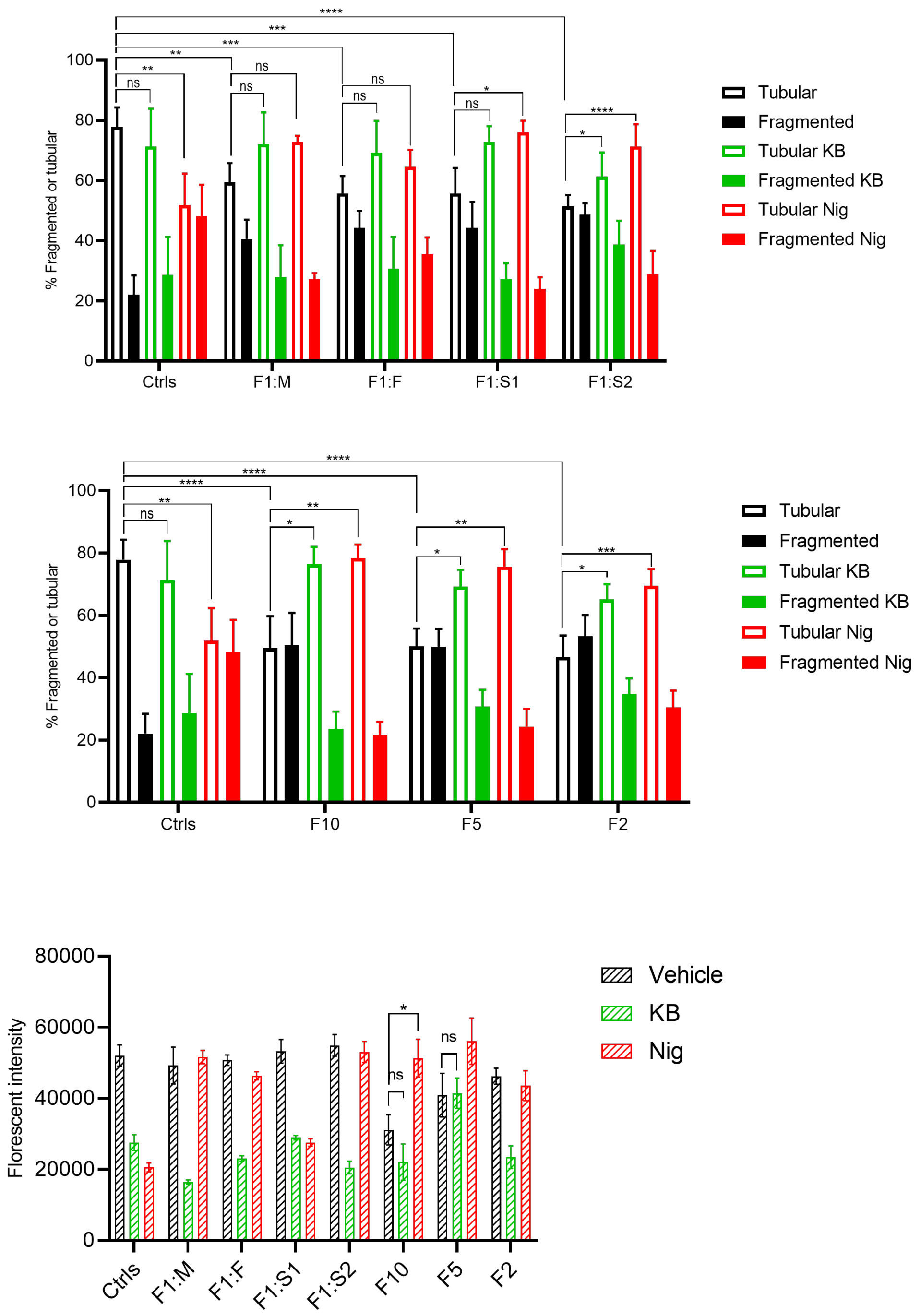

% Fragmented or tubular
100
80
60
40
20
0
Ctrls
F1:M
F1:F
F1:S1
F1:S2
ns
**
***
****
*
Tubular
Fragmented
Tubular KB
Fragmented KB
Tubular Nig
Fragmented Nig
Ctrls
F10
F5
F2
80000
60000
40000
20000
Florescent intensity
Vehicle
KB
Nig


**Figure S1C**

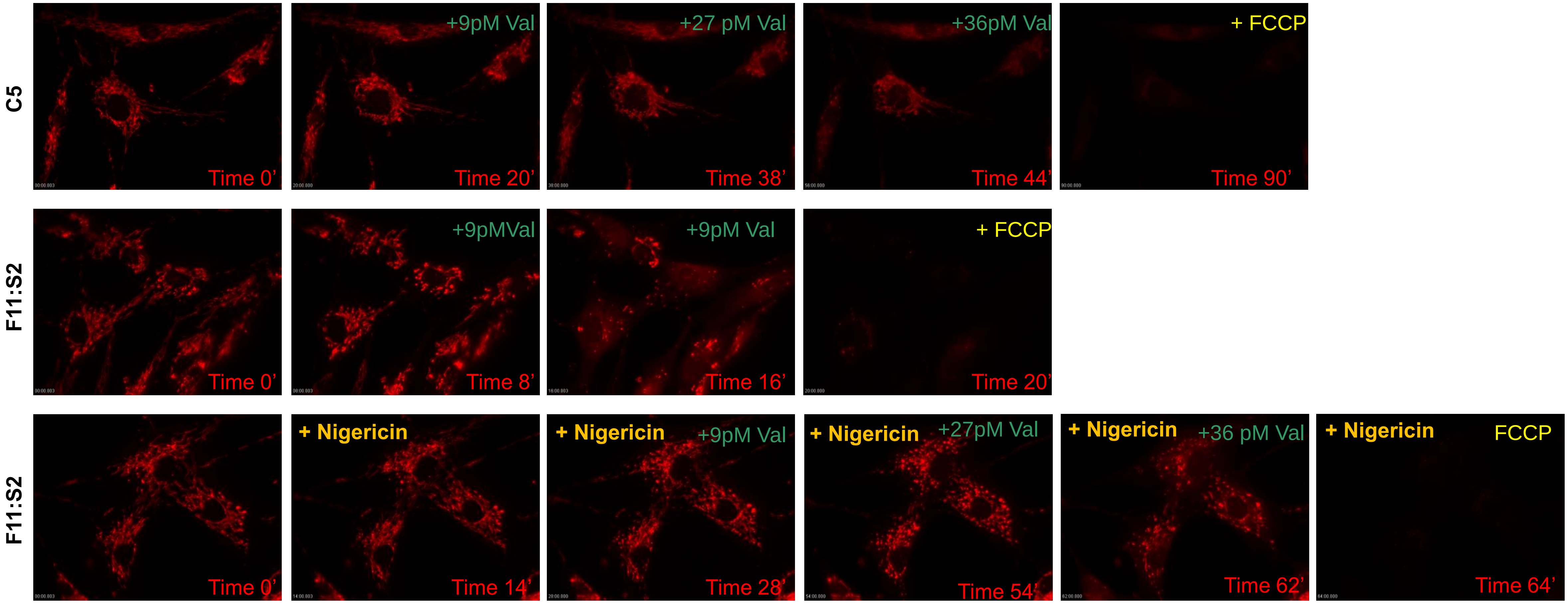
C5
Time 0'
+9pM Val
Time 20'
+27 pM Val
Time 38'
+36pM Val
Time 44'
+ FCCP
Time 90'
F11:S2
Time 0'
+9pMVal
Time 8'
+9pM Val
Time 16'
+ FCCP
Time 20'
F11:S2
Time 0'
+ Nigericin
Time 14'
+ Nigericin
+9pM Val
Time 28'
+ Nigericin
+27pM Val
Time 54'
+ Nigericin
+36 pM Val
Time 62'
+ Nigericin
FCCP
Time 64'

Figure S1D

**Figure S1. Mitochondrial morphology in function of *LETM1* variants.**

**A)** Rescue experiments with nigericin and ketone bodies

Summary of confocal images of fibroblasts exposed to vehicle (ETOH); nigericin or ketone bodies (KB)as indicated. Shown are representative images all taken at the laser intensity set at the same level forall samples, except at a 5 fold increase when indicated (KB intensity x 5) for F1:M and F1:F. Scale bars:overview images 10 μm, details: 5 μm

**B)** Detrimental effect of galactose on patient fibroblasts

Mitochondria of fibroblasts from control C3, C4, F1:M, F1:S1 and F11:S2 grown in medium supplemented with 25 mM glucose (HG) or 5 mM galactose (Gal) for at least 48 hours and immunolabeled with anti-TOM20 (red) antibody. Scale bar: 10 μm.

**C)** Quantitative comparison of mitochondrial morphotypes and membrane potential.

Upper and middle panels show the quantitative comparison between tubular versus fragmented shaped mitochondria given in percentage per cell under vehicle, ketone body and nigericin conditions, (Ctrls: mean of C1 and C2; the same control values were used in the upper and middle panels). Bottom panel shows the quantification of the membrane potential as a mean grey value per cell. N= 3 independent experiments, a minimum of 10 cells (with an average of 200 mitochondria per cells being analysed) is pooled per experiment. Vehicle: ethanol, Nig: nigericin, KB: ketone body, Statistics for morphotypes: mean ±SEM, non-parametric Kruskal-Wallis multiple comparisons test was performed of patient against healthy donor, *p<0.04, **p<0.004, ***p<0.0008, ****p<0.0001, non-significant ns>0.05. Statistics for lower panel vehicle F10 vs Ctrls ***p=0.0006; F10 vehicle vs nigericin F10 *p=0.0236, F1:S1 vehicle vs nigericin, and vehicle vs ketone body ***p<0.0002; F1:S2 vehicle vs ketone body ****p<0.0001; F2 vehicle vs ketone body ***p=0.0001, others n.s, non-parametric Kruskal-Wallis multiple comparisons test was performed.

**D)** Real time study of mitochondria from F11:S2

Time lapse recording of control (C5) and F11:S1 fibroblasts stained with TMRM (10 nM) and challengedwith increasing concentration of valinomycin in the absence or presence of nigericin over time. Complete depolarisation was achieved with FCCP (4 μM) at the end of the time course.

# Figure S2

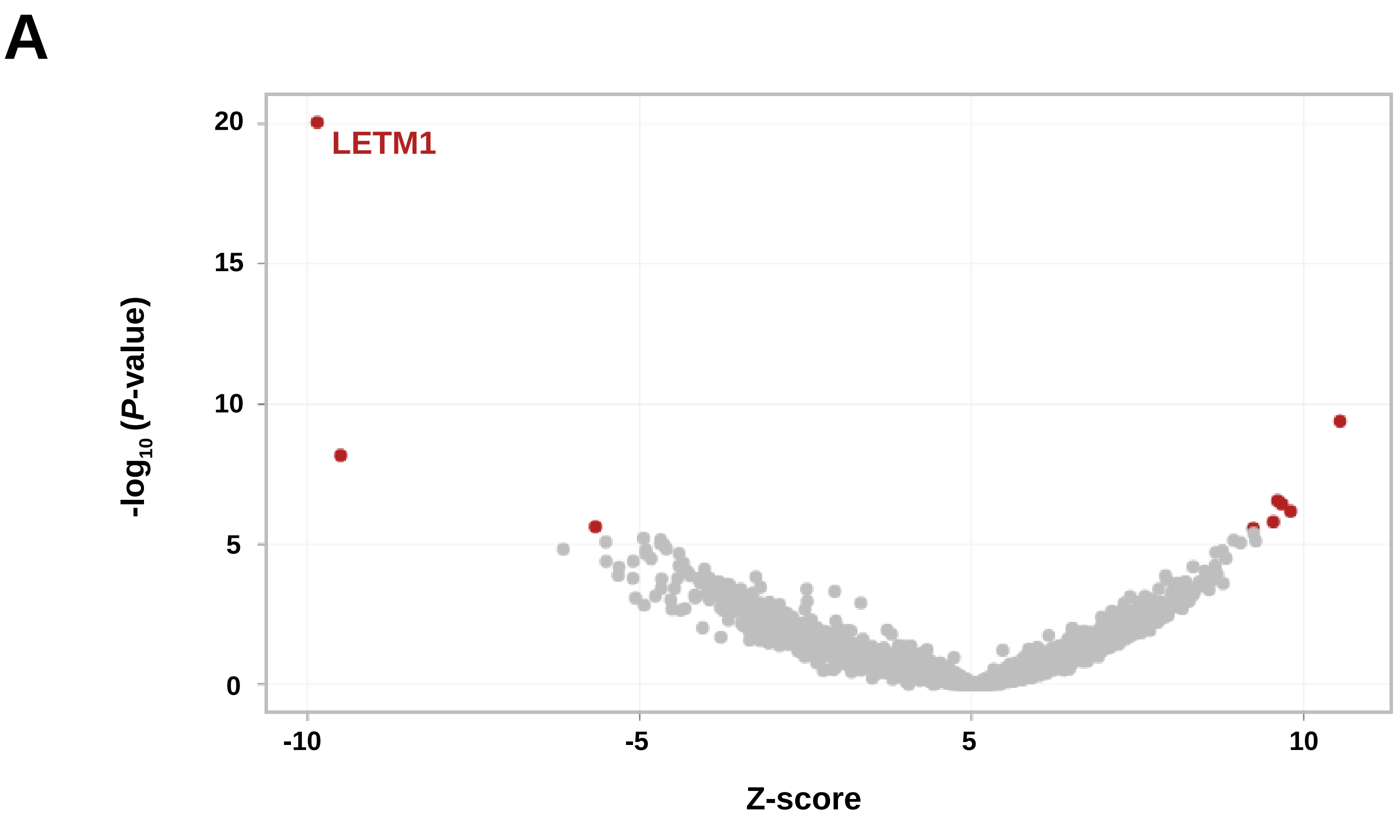
A
LETM1
-log10 (P-value)
20
15
10
5
0
-10
-5
5
10
Z-score

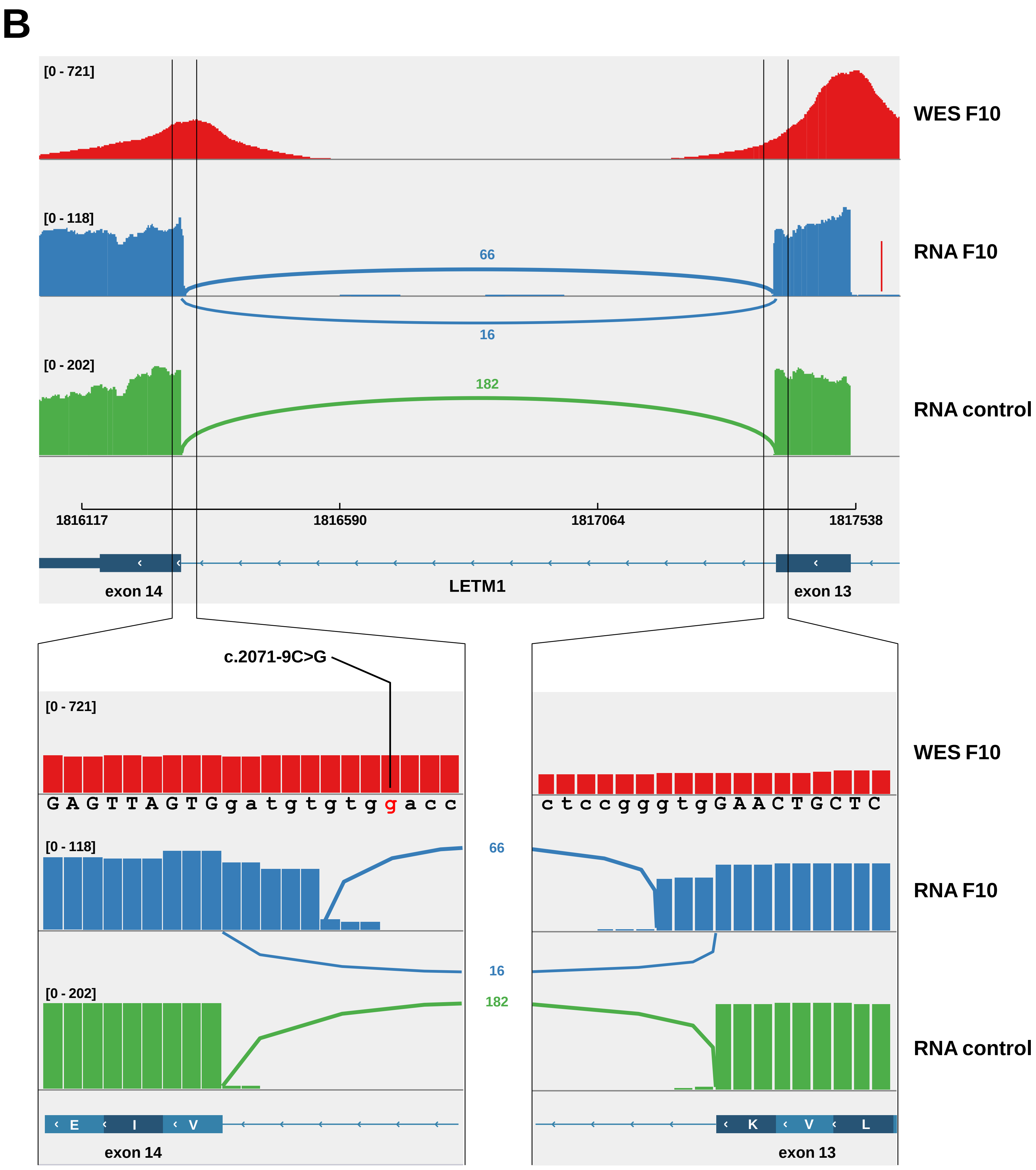
B
[0 - 721]
WES F10
[0 - 118]
66
16
RNA F10
[0 - 202]
182
RNA control
1816117
1816590
1817064
1817538
exon 14
LETM1
exon 13
c.2071-9C>G
[0 - 721]
G A G T T A G T G g a t g t g t g g a c c
c t c c g g g t g G A A C T G C T C
WES F10
[0 - 118]
66
16
RNA F10
[0 - 202]
182
RNA control
E
I
V
K
V
L
exon 14
exon 13

**Figure S2. RNA-seq reveals aberrant expression and splicing of *LETM1* in F10**

A. Volcano plot of F10 gene expression depicting the gene-level significance (-log10(P), y-axis) versus Z-score, with *LETM1* labeled among the expression outliers (red dots). B. *LETM1* Sashimi plot showing ES and RNA-seq of F10 (WES F10 in red and RNA F10 in blue, respectively) compared to the control RNA-seq (RNA control, in green), and the gene model of the RefSeq annotation below, with highlighted partial intron 13 retention caused by homozygous splice region variant c-2071-9C>G (C>G annotation based on the antisense strand) in F10, leading to a frameshift p.Val691*fs*Ter4*and nonsense-mediated decay.

Figure S3

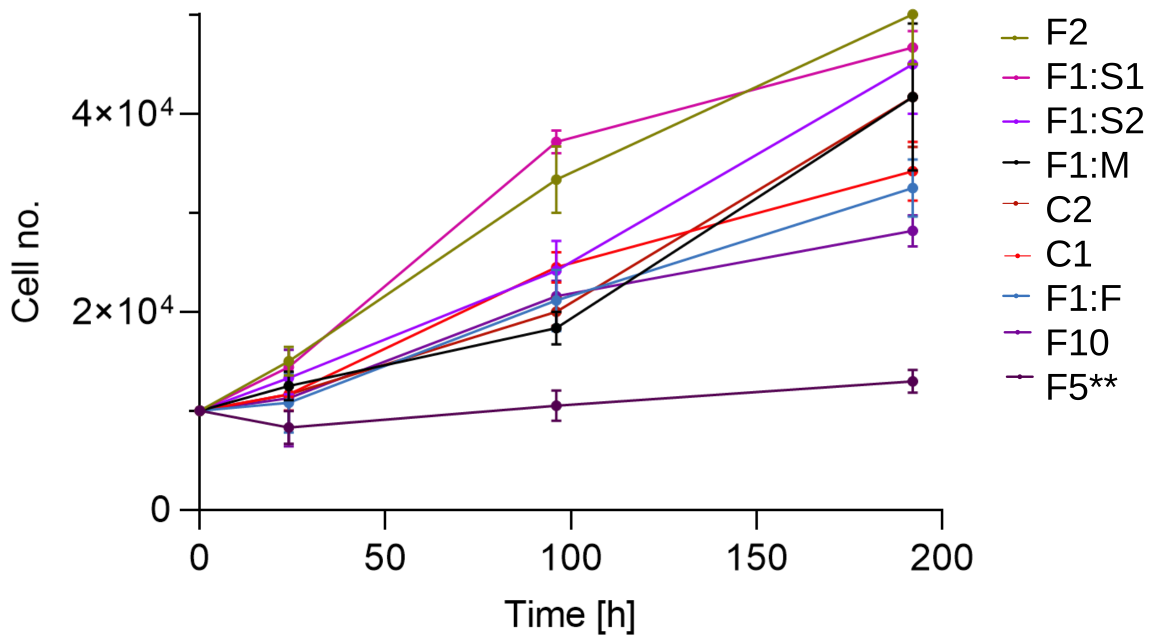

Cell no.
4×10⁴
2×10⁴
0
0
50
100
150
200
Time [h]
F2
F1:S1
F1:S2
F1:M
C2
C1
F1:F
F10
F5**

**Figure S3. Different effects of *LETM1* variants on fibroblast proliferation**

Overview of the proliferation of fibroblasts from controls (C1 and C2) and case (F1, F2, F5, F10). Cells were seeded at equal cell number and grown for 8 days, counted every second day as indicated, n=3 independent experiments. Statistics: ns>0,05, **p=0,007 using non-parametric Kruskal-Wallis multiple comparisons test comparing each of the cases against the controls.

## Figure S4

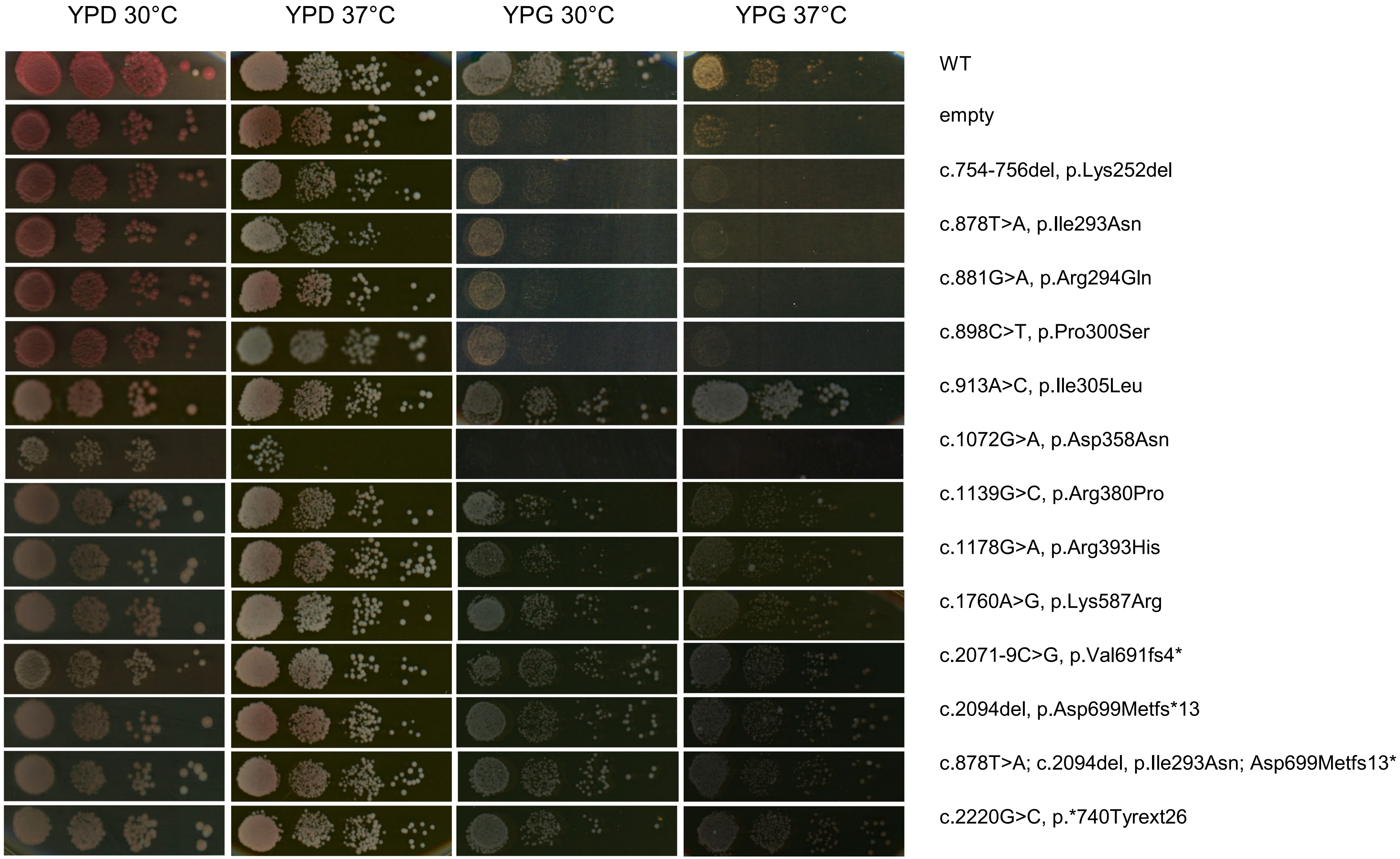

YPD 30°C
YPD 37°C
YPG 30°C
YPG 37°C
WT
empty
c.754-756del, p.Lys252del
c.878T>A, p.Ile293Asn
c.881G>A, p.Arg294Gln
c.898C>T, p.Pro300Ser
c.913A>C, p.Ile305Leu
c.1072G>A, p.Asp358Asn
c.1139G>C, p.Arg380Pro
c.1178G>A, p.Arg393His
c.1760A>G, p.Lys587Arg
c.2071-9C>G, p.Val691fs4*
c.2094del, p.Asp699Metfs*13
c.878T>A; c.2094del, p.Ile293Asn; Asp699Metfs13*
c.2220G>C, p.*740Tyrext26

**Figure S4. Growth phenotype of *S. cerevisiae letm1Δ* expressing or not human *LETM1* wild-type or variants.**

Serial dilutions of *S. cerevisiae letm1Δ* strains expressing pVT-103U empty (e) or carrying LETM1 WT, or LETM1 with the indicated variants were spotted onto fermentable (YPD) and non-fermentable (YPG) media and grown at 30°C or 37°C.

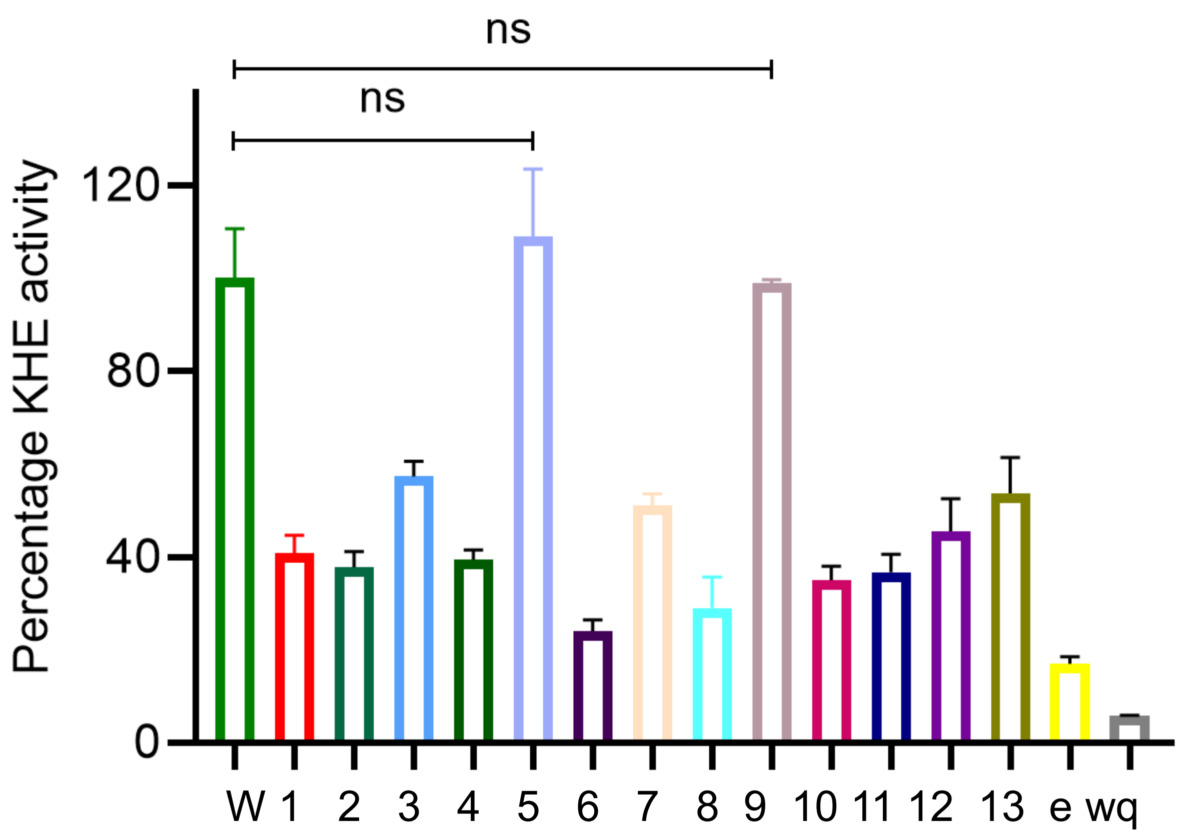
ns
ns
Percentage KHE activity
120
80
40
0
W
1
2
3
4
5
6
7
8
9
10
11
12
13
e
wq

**Figure S5**

**Figure S5. Evaluation of the relative KHE activity**

Results shown in Figure 5 were evaluated in function of the swelling amplitudes, the swelling time between t=0 and t= 18 sec and the initial OD and are represented relatively to *S. cerevisiae ΔLETM1* expressing human *LETM1* wildtype. Statistics: non-pathogenic variants: n.s, all other ****p>0.0001 by one way ANOVA Dunnett´s multiple comparison test, means are ± SEM.

**Table S1. Extended version of Table 2.**

Available as a separate excel file.

**Table S2. Enzyme investigations in muscle sample of Family 5**

| | **Patient** | **Normal range** |
|---|---|---|
| mtDNA/nDNA copy number | 4367 | 887-2066 |
| **Enzyme activities [nmol/min/mg protein]** | | |
| Citrate synthase (CS) | 349 | 134-260 |
| Complex I | 16 | 18-59 |
| Complex I+III | 23 | 18-45 |
| Complex II | 147 | 28-69 |
| Complex II+III | 71 | 16-43 |
| Complex III | 203 | 149-480 |
| Cytochrome c oxidase | 197 | 148-392 |
| Complex V | 230 | 60-223 |
| Protein concentration [mg/ml] | 3,02 | |
| **Relative enzyme activities** | | |
| Complex I/CS | 0,05 | 0,11-0,26 |
| Complex I+III/CS | 0,07 | 0,11-0,24 |
| Complex II/CS | 0,42 | 0,14-0,43 |
| Complex II+III/CS | 0,20 | 0,10-0,29 |
| Complex III/CS | 0,58 | 0,75-2,32 |
| Cytochrome c oxidase/CS | 0,56 | 0,83-2,40 |
| Complex V/CS | 0,66 | 0,30-0,75 |

**Supplemental Material and Methods**

**Skin and muscle biopsy and primary fibroblasts culture**

Affected individuals F1:S1, F1:S2 and both parents (F1: Father (F1:F) and F1: Mother (F1:M)), F2:S1, F5:S1, F10:S1, and F11:S2 provided skin biopsies. Skin fibroblast cell lines were established in RPMI medium supplemented with 20% fetal bovine serum (FBS) and 1% penicillin/streptomycin (P/S) (Thermo Fisher Scientific) at 37° C in a humidified atmosphere with 5% $CO_2$. Cell lines were maintained in an incubator set to 37º C and 5% $CO_2$ and cultured in Dulbecco's modified Eagle medium DMEM with high glucose, GlutaMAX™, and pyruvate (ThermoFisher, cat# 31966-021), supplemented with 10% fetal bovine serum (FBS) and uridine 50 μg/ml (Sigma), or DMEM with glutamine, w/o glucose and w/o pyruvate (ThermoFisher, cat# 11966-025) supplemented with 0.3 mM β-hydroxybutyrate (ketone bodies (KB)) (Cayman Chemical, cat# 14148), 10% FBS, and 1% P/S for 24 hours. In some experiments, fibroblasts of F1 and F11 were grown in absence of uridine. The galactose regime employed glucose-free DMEM medium (Life Technologies) with the addition 5 mM of galactose and 10% dialyzed FBS. Cells were regularly screened for and confirmed free mycoplasma (MycoAlert Lonza kit cat# LT07-418 or Look Out Mycoplasma PCR Detection Kit (Sigma).

Muscle biopsies were provided by affected individuals F11:S1, F11:S2 and F5:S1. For F5, 50-100 mg tissue were transferred into sterile SIMPORT tubes (T405-2A) and 10-20-fold volume of SEKT buffer (250 mM saccharose, 2 mM EGTA, 40 mM KCl, 20 mM Tris; pH 7.4) was added. Samples were homogenized with an Ultra-Turrax at 20.500 U/min and the homogenate transferred to the glass Potter S- Homogenisator for 10 – 12 times homogenisation strokes on ice. The homogenate was transferred into a SIMPORT tube and centrifuged at 600 x g for 10 min at 4 °C. Aliquots were stored at -70°C. For F11 30 mg of muscle samples were homogenized in RIPA buffer (SIGMA) in the presence of 1X protease and phosphatase inhibitor cocktail (Thermo scientific). Subsequently, samples were centrifuged at 12000 rpm for 20 min at 4°C and supernatant was collected. Protein concentration was evaluated using BCA protein assay kit (Thermo scientific) and samples were read at λ 562 nm using an Agilent 8453 spectrophotometer.

**Immunoblotting analysis**

Total lysates of fibroblast cultures from F2, F10 and C2 were lysed in RIPA buffer (50 mM Tris-HCl pH 8.0, 150 mM NaCl, NP40 1%, 0.5% Sodium deoxycholate, 0.1% SDS, 1 mM EDTA with proteinase inhibitors (Sigma Aldrich, St. Louis, MS, USA). Protein lysates (15 μg/lane), as well as TCA-precipitated proteins from yeast total cell extracts (50 μg/lane) or cytoplasmic fractions, or isolated crude mitochondria (25 μg/lane) were separated by 12 or 15% SDS PAGE after determination of protein

concentration by BCA assay. After the transfer of proteins onto nitrocellulose membrane, membranes were blocked in 2% BSA-TBS-T and probed with the indicated antibodies (Table S2). Reactive bands were detected using SuperSignal West pico Chemiluminescent Substrate (Thermoscientific). Fibroblasts from F1, F11, C3, C4 were lysed in 0.1% n-dodecyl-D-maltoside (DDM, Sigma), 1% SDS, 50 U benzonase (Novagen), 1:50 (v/v) protease inhibitor cocktail (Roche), and 1:100 (v/v) phosphatase inhibitor (Cell Signaling). Fibroblasts lysates or 30 µg of muscle mitochondria were separated by 12% SDS PAGE, proteins were transferred onto polyvinylidene difluoride (PVDF) membrane. After blocking in 5% non-fat dry milk in PBS-T and probing with indicated antibodies (and Table S2), reactive bands were detected using Lite Ablot Extend Long Lasting Chemiluminescent Substrate (Euroclone, Pero (Mi), Italy). For mitochondria isolated by differential centrifugation and resuspended in SEKT buffer from F5 and C5 muscle, proteins were separated on 10% SDS PAGE and transferred onto nitrocellulose membrane, blocking reagent was from Roche in TBS-T, antibodies were as indicated in Table S2, and bands were detected using Lumi-Light PLUSPOD substrate (Roche).

Densitometry analysis was performed using Quantity One software (BioRad, Hercules, CA, USA) or Image Lab 6.1.0 software (Bio-Rad Laboratories, Inc.).

**List of Antibodies used for immunoblotting:**

| **Antibody** | **Company** |
|---|---|
| LETM1 1: 1000 | Abnova #H00003954-M03 |
| LETM1 1: 1000 | Aviva #OAAB12878 |
| LETM1 1: 1000 | Santa Cruz #sc-163013 |
| LETM1 1:1000 | Abnova H00003954-PW1 |
| Porin 1: 1000 | Invitrogen #459500 |
| Cox2 1: 1000 | Invitrogen #459150 |
| Actin 1: 1000 | Invitrogen #MA5-11869 |
| Total OXPHOS cocktail 1:1000 | Abcam #ab110411 |
| COX4 1: 1000 | Cell signaling #4850 |
| NDUFA9 1:1000 | Abcam #ab14713 |
| NDUFB8 1:2000 | Abcam #ab110242 |
| MT-CO2 1:1000 | Abcam #ab110258 |
| SDHB 1:200 | Abcam ab #14714 |
| UQCRC2 1:1000 | Abcam #ab14745 |
| COXIV 1:1000 | Abcam # ab14744 |
| TOM20 1:600 | Abcam #ab186735 |
| DNA 1:250 | Progen #AC-30-1 |
| VINCULIN 1:5000 | Abcam #ab14714 |
| NDUFS4 1:1000 | Sigma#WH0004734M1 |
| SDHA 1:2000 | Abcam ab #14715 |
| UQCRC2 1:1500 | Abcam #14745 |
| MT-CO2 1:1000 | Abcam #ab79393 |
| ATP5F1A 1:2000 | Abcam #ab14748 |
| VDAC1 1:2000, or 1:3000 | Abcam #ab14734, Abcam #ab15895 |

| | |
|---|---|
| CS 1:3000 | THP #NBP2-43648 |
| GAPDH 1:5000 | Trevigen #2275-PC-100 |
| NDUFB8 rabbit polyclonal, 1:500 | Abcam #ab192878 |
| MT-CO1 mouse monoclonal, 1:1000 | Abcam #ab14705 |
| Peroxidase Mouse IgG | Thermo Fisher #31430 |
| Peroxidase Rabbit IgG | Cell Signaling #7074p2 |
| Peroxidase Goat IgG | Jackson ImmunoResearch Laboratories #305-035-003 |
| Alexa Fluor 647Mouse IgG2a | Jackson ImmunoResearch, Laboratories # 115-607-186 |
| DyLight 488 1:1000 | Vector Laboratories #DI-2488 |
| horseradish peroxidase-(HRP) 1:100) | EnVisionkit, Dako |

**Immunohistochemical staining**

FFPE muscle tissue was cut with a microtome in 4 µm slides. The slides were heated for 1 h at 60°C. After rehydration (3 x 4 min xylol, 3 x 3 min isopropanol) slides were washed 3 x 3 min in ddH2O. Heat-induced antigen retrieval was performed in 1 mM EDTA, 0.05% Tween-20, pH 8 for 40 min at 95°C. Slides were allowed to cool down to room temperature. After washing 3 x 3 min in ddH2O and 3 x 3 min in PBS-T endogenous peroxidase activity was blocked with peroxidase block (DAKO envision kit) for 5 min. Slides were washed 3 x 3 min in PBS-T. Antibodies are listed in Table S2. All antibodies were diluted in Dako antibody diluent with background-reducing components (Dako, Glostrup, Denmark). Thereafter, the slides with incubated with the respective DAKO polymer (rabbit or mouse) for 1 h at RT. After washing 3 x 3 min in PBS-T DAB development was performed. Samples were rinsed 5 min in tap water to stop the reaction. Nuclei were stained with hemalaun for 3-5 min, briefly rinsed in 3% HCl-EtOH and blueing was done for 10 min in running tap water. After dehydration in isopropanol and xylol mounting was done in Histokit.

**Spectrophotometric determination of the OXPHOS enzyme activity**

Muscle 600 × g homogenates or isolated fibroblast mitochondria were used for determination of enzymatic activities of the OXPHOS complexes. Enzyme activities of the OXPHOS complexes were determined as previously described.[1] Rotenone-sensitive complex I activity was measured spectrophotometrically as NADH/decylubiquinone oxidoreductase at 340 nm. The activities of citrate synthase, complex IV (ferrocytochromec/oxygen oxidoreductase), and oligomycin-sensitive ATP synthase activity of the F1FO ATP synthase (complex V) were determined as previously described.[2] The reaction mixture for the ATPase activity measurement was treated for 10 s with an ultra-sonifier (Bio cell disruptor 250, Branson, Vienna, Austria). The reaction mixture for the measurement of

complex III activity contained 50 mM potassium phosphate buffer pH 7.8, 2 mM EDTA, 0.3 mM KCN, 100 µM cytochrome c, 200 µM reduced decyl-ubiquinol. The reaction was started by addition of the 600 x g homogenate. After 3–4 min the reaction was inhibited with 1 µM antimycin A. All spectrophotometric measurements (Uvicon 922, Kontron, Milan, Italy) were performed at 37°C.

**Cell imaging**

Fibroblasts from F1, F2, F5, F10, C1 and C2 were seeded onto 8 well dishes (Ibidi, cat#80826), stained with 50 nM Mitotracker Red CMXRos. Live staining was either fixed or followed by immunostaining (as described in Wilfinger et al.)[3] Image acquisition was done using a Zeiss laser confocal microscope LSM 880 and RFP channel, Plan-Apochromat 40x or 63x/1.40 Oil DIC M27 lens and pinhole 65 µm. In other cases, fibroblasts from F1 and F11 were grown on coverslips and immunostained as described in.[4] Samples were imaged either on a SP5 TCS Inverted Confocal Microscope (Leica Biosystem) using an immersion objective with a numerical aperture of 63x or 100x/1.4 Oil or on Nikon Ti Inverted Confocal Microscope using 60x immersion Oil objective. Z stack of red, green, and blue images using a step size of either 0.3 or 0.125 lm was acquired sequentially and merged using ImageJ. Laser power, gain and offset parameters were kept constant for each experiment. Any adjustments to brightness and contrast were applied linearly to all images in a comparison. For single immunostaining of the mitochondrial network with TOM20, samples from F11 were fixed with methanol:acetone (2:1) for 10 min in 5% BSA-PBS, visualized using the Alexa Fluor 647 secondary antibody, and images were acquired with a fluorescence-inverted microscope (Leica DMi8). An average of eight image planes wasobtained along the z-axis at 0.2 µm increments using the LASX 3.0.4 (Leica) software.

For transmission electron microscopy, fibroblasts were grown to semi-confluency on Aclar coverslips (Science Services; Munich). Primary fixation was done with 2.5% glutaraldehyde in 0.1 M sodium cacodylate buffer, pH 7.3 and postfixation with 1% $OsO_4$, dehydration in a series of ethanol, followed by sample infiltration in mixtures of acetone and Agar 100 resin. For final infiltration with pure resin, the Aclar coverslips were placed on glass slides with the cell layers facing up. After infiltration of the droplets of resin, Eppendorf tubes with their bottoms and lids cut off were placed above the samples. After an initial heat polymerization at 60°C, the Eppendorf tubes were filled with resin, and polymerization continued for two days. The Aclar coverslips were removed from the resin blocks. Thin sections (70-80 nm) oriented in parallel to the contact surface with the substratum were cut with an ultramicrotome Ultracut S (LEICA Microsystems, Vienna, Austria), mounted on 200 mesh copper grids, counterstained with neodymium(III)-acetate[5] for 50 min followed by lead citrate for 8 min and examined at 120 kV in a ZEISS Libra 120 transmission electron microscope. Images were acquired using

a bottom stage digital camera, TRS (4 megapixels), and ImageSp-professional software (Tröndle, Moorenweis, Germany).

**RNA sequencing**

Primary skin fibroblasts from patient S3 from Family 10 were used for RNA-sequencing as descirbed in Yepez et al.[6] In short, RNA was isolated from the cells using the RNeasy mini kit (Qiagen, Hilden, Germany) according to the manufacturer's protocol. RNA integrity number (RIN) was subsequently measured with the Agilent 2100 BioAnalyzer (RNA 6000 Nano Kit, Agilent Technologies, Santa Clara, CA, USA). Library preparation for the strand-specific RNA-sequencing was done according to the TruSeq Stranded mRNA Sample Prep LS Protocol (Illumina, San Diego, CA, USA). Library´s quality and quantity were determined with the Agilent 2100 BioAnalyzer and the Quant-iT PicoGreen dsDNA Assay Kit (Life Technologies, Carlsbad, CA, USA). Library was sequenced as 100 bp paired-end runs on Illumina HiSeq4000 platform. Reads from RNA-seq were demultiplexed and mapped with STAR v2.7.0a to the hg19 genome assembly.[7] Upon alignment, data were analyzed using the computational workflow DROP.[6]

**Yeast serial dilution**

All strains were grown overnight in selective media (SD-URA, synthetic dextrose media lacking uracil media following the protocol of Nowikovsky et al.[8] and diluted to final OD600 of 1; transferred 1:1, 1:10, 1:100 and 1:1000 to a microtiter plate, stamped onto YPD (2% glucose) and YPG (3% glycerol) plates and incubated at 30 or 37°C. YPD plates were incubated 2-4 days, YPG 30°C 4-6 days, and YPG 37°C 7-9 days.

**Primers used for site-directed mutagenesis.**

| # | **Variant** | **Forward primer 5´-3´** | **Reverse primer 5´-3´** |
|---|---|---|---|
| 1 | c.754-756del | GAGCTTCGGGTCAAGCTG | CTTCAGCCTCTCCTCCTTG |
| 2 | c.878T>A | TTCCAGAAGAACCGGGAAACAG | AAACACAGAGAAGTCTTTGG |
| 3 | c.881G>A | CAGAAGATCCAGGAAACAGGG | GAAAAACACAGAGAAGTCTTTG |
| 4 | c.898C>T | AGGGGAGAGGTCCAGCAATGA | GTTTCCCGGATCTTCTGGAAAAAC |
| 5 | c.913A>C | CAATGAGGAACTCATGCGTTTTTCCAAATTATTTG | CTGGGCCTCTCCCCTGTT |
| 6 | c.1072G>A | CATAAAGGCAAACGACAAGCTGATTGC | GAGCGCAGCCGCATGGTA |

| | | | |
|---|---|---|---|
| 7 | c.1139G>C | GCAGCGTGTCCGGCACGAGGC | CTGCAGCTCCTTGACATTCAGG |
| 8 | c.1178G>A | ACGGAAGACCACCTGAGGGGTCAGC | GACGCCCAGGGCCCGCAT |
| 9 | c.1760A>G | CAGGAGATCAGGAAGGAACTTTC | CAAGTCCTCGCTGTAGTC |
| 10 | c.2071-9C>G(1) | GTAGGTGATTGAGCTGGTGGAC | ACACCTTGACGAGGTCGTCGAT |
| 11 | c.2094del(2) | GATGTTCACATCTCCACC | TCTTTGTCCACCAGCTCA |
| 12 | c.878T>A in LETM1-11 (3) | TTCCAGAAGAACCGGGAAACAG | AAACACAGAGAAGTCTTTGG |
| 13 | c.2220G>C(4) | GTCACCCTGGCAAGGGCCGTGAGGGCGATTGC TTTGTGGTTTACCTTTTACCCATACGATG | GGCACAGCAGGAGGACAGGTGCCC AGGCCAGTGGTTGTAGCTCTTCACCT CTGCGAC |

(1) c.2071-9C>G leads to splice site defect characterized by retention of the first 8 nucleotides upstream of exon 14, the set of primers is used to insert the nucleotides. (2) c.2094del results in a frameshift and a premature introduction of a stop codon (at position 710). (3) To generatethe compound variant, LETM1 #2 was used as a template for introducing the second point variant (2094del). (4) c.2220G>C results in a longer protein of 765 aa.

**Statistical analysis**

All statistical analyses were done for independent experimental replicates using GraphPad (La Jolla, CA) Prism v8.0.1. for Windows. Tests and individual p values are indicated in the figure legends. Data are presented as mean ± SD unless other specified.

**Supplemental acknowledgements**

The families were collected as part of the SYNaPS Study Group collaboration funded by The Wellcome Trust and strategic award (Synaptopathies) funding (WT093205 MA and WT104033AIA). This research was conducted as part of the Queen Square Genomics group at University College London, supported by the National Institute for Health Research University College London Hospitals Biomedical Research Centre. We acknowledge Exeter Genomics Laboratory for providing the exome testing.

This study was supported by the Italian Ministry of Health (Ricerca Corrente, and Ricerca Finalizzata RF-2016-02361241), the Pierfranco e Luisa Mariani Foundation (CM23). T.B.H. was supported by the Deutsche Forschungsgemeinschaft (DFG, German Research Foundation) – 418081722, 433158657. RM and RWT are supported by the Wellcome Centre for Mitochondrial Research (203105/Z/16/Z), the Medical Research Council (MRC) International Centre for Genomic Medicine in Neuromuscular Disease (MR/S005021/1), the Mitochondrial Disease Patient Cohort (UK) (G0800674), the UK NIHR Biomedical Research Centre for Ageing and Age-related disease award to the Newcastle upon Tyne Foundation Hospitals NHS Trust, the Lily Foundation and the UK NHS Specialist Commissioners which funds the "Rare Mitochondrial Disorders of Adults and Children" Diagnostic Service in Newcastle upon Tyne. RWT also receives financial support from the Pathological Society. AA was supported by a PhD studentship funded by the Kuwait Civil Services Commission. JM are support by the German Federal Ministry of Education and Research (BMBF, Bonn, Germany) and Horizon2020 through the EJP RD project GENOMIT (01GM1920A, genomit.eu). CL, DG, and SBW are funded by the BMBF through ERA PERMED2019-310 – Personalized Mitochondrial Medicine (PerMiM): Optimizing diagnostics and treatment for patients with mitochondrial diseases and by the E-Rare project GENOMIT. HP was supported by the BMBF and Horizon2020 through the EJP RD project GENOMIT (01GM1920A), the ERA PerMed project PerMiM (01KU2016A) and mitoNET (01GM1906B). JAM was supported by the ERA-Net E-Rare project GENOMIT Austrian Science Fund (FWF) I4704-B. CL, AC, and DG are members of the European Reference Network for Rare Neuromuscular Diseases (ERN EURO-NMD). CL and AC were supported by the project GENOMIT (J42F19000030006-RE17) and the Italian Ministry of Health RF-2006-02361495.

**Supplemental references**

1. Kusikova, K., Feichtinger, R.G., Csillag, B., Kalev, O.K., Weis, S., Duba, H.C., Mayr, J.A.,

Weis. D. (2021). Case Report and Review of the Literature: A New and a Recurrent Variant in the VARS2 Gene Are Associated With Isolated Lethal Hypertrophic Cardiomyopathy, Hyperlactatemia, and Pulmonary Hypertension in Early Infancy. Front Pediatr. *9,* 660076.

2. Rustin, P., Chretien, D., Bourgeron, T., Gérard, B., Rötig, A., Saudubray, J.M., and Munnich, A. (1994). Biochemical and molecular investigations in respiratory chain deficiencies. Clin ChimActa. *228* :35-51
3. Wilfinger, N., Austin, S., Scheiber-Mojdekhar, B., Berger, W., Reipert, S., Praschberger, M., Paur, J.,Trondl, R., Keppler, B. K., Zielinski, C. C., and Nowikovsky, K. (2016). Novel p53-dependent anticancer strategy by targeting iron signaling and BNIP3L-induced mitophagy. Oncotarget. *7*, 1242–1261.
4. Durigon, R., Mitchell, A.L., Jones, A.W., Manole, A., Mennuni, M., Hirst, E.M., Houlden, H., Maragni, G., Lattante, S., Doronzio, P.N., et al. (2018).LETM 1 Couples Mitochondrial DNAMetabolism and Nutrient Preference . EMBO Molecular Medicine. *10*, 1–20.
5. Kuipers, J., and Giepmans, B.N.G. (2020). Neodymium as an alternative contrast for uranium inelectron microscopy, Hitochem. Cell. Biol. 153, 271-277.
6. Yepez, V.A., Mertes, C., Muller, M.F., Klaproth-Andrade, D., Wachutka, L., Fresard, L., Gusic, M.,Scheller, I.F., Goldberg, P.F., Prokisch, H., et al. (2021). Detection of aberrant gene expression events in RNA sequencing data. Nature protocols 16, 1276-1296.
7. Dobin A, Davis CA, Schlesinger F, Drenkow J, Zaleski C, Jha S, et al. STAR: ultrafast universal RNA-seq aligner. Bioinformatics. 2013 Jan;29(1):15–21.
8. Nowikovsky, K., Froschauer, E.M., Zsurka, G., Samaj, J., Reipert, S., Kolisek, M., Wiesenberger, G., Schweyen, R.J. (2004). The LETM1/YOL027 gene family encodes a factor of the mitochondrialK+ homeostasis with a potential role in the Wolf-Hirschhorn syndrome. J Biol Chem. *279*, 30307-30315.
